# Functionality and Robustness of Injured Connectomic Dynamics in *C. elegans*: Linking Behavioral Deficits to Neural Circuit Damage

**DOI:** 10.1371/journal.pcbi.1005261

**Published:** 2017-01-05

**Authors:** James M. Kunert, Pedro D. Maia, J. Nathan Kutz

**Affiliations:** 1 Department of Physics, University of Washington, Seattle, Washington, United States of America; 2 Department of Applied Mathematics, University of Washington, Seattle, Washington, United States of America; Oxford University, UNITED KINGDOM

## Abstract

Using a model for the dynamics of the full somatic nervous system of the nematode *C. elegans*, we address how biological network architectures and their functionality are degraded in the presence of focal axonal swellings (FAS) arising from neurodegenerative disease and/or traumatic brain injury. Using biophysically measured FAS distributions and swelling sizes, we are able to simulate the effects of injuries on the neural dynamics of *C. elegans*, showing how damaging the network degrades its low-dimensional dynamical responses. We visualize these injured neural dynamics by mapping them onto the worm’s low-dimensional postures, i.e. eigenworm modes. We show that a diversity of functional deficits arise from the same level of injury on a connectomic network. Functional deficits are quantified using a statistical shape analysis, a procrustes analysis, for deformations of the limit cycles that characterize key behaviors such as forward crawling. This procrustes metric carries information on the functional outcome of injuries in the model. Furthermore, we apply classification trees to relate injury structure to the behavioral outcome. This makes testable predictions for the structure of an injury given a defined functional deficit. More critically, this study demonstrates the potential role of computational simulation studies in understanding how neuronal networks process biological signals, and how this processing is impacted by network injury.

## Author Summary

Neurodegenerative diseases such as Alzheimer’s disease, Creutzfeldt-Jakob’s disease, HIV dementia, Multiple Sclerosis and Parkinson’s disease are leading causes of cognitive impairment and death worldwide. Similarly, traumatic brain injury, the signature injury of the Iraq and Afghanistan wars, affects an estimated 57 million people. All of these conditions are characterized by the presence of focal axonal swellings (FAS) throughout the brain. On a network level, however, the effects of FAS remain unexplored. With the emergence of models which simulate an organism’s full neuronal network, we are poised to address how neuronal network performance is degraded by FAS-related damage. Using a model for the full-brain dynamics of the nematode *Caenorhabditis elegans*, we are able to explore the loss of network functionality as a function of increased neuronal swelling. The relatively small neuronal network generates a limited and tractable set of functional behaviors, and we develop metrics which characterize how these behaviors are impaired by network injuries. These metrics quantify the severity of TBI and/or neurodegenerative disease, and could potentially be used to construct diagnostic tools capable of identifying various cognitive deficits. Additionally, we apply classification trees to our results to make predictions about the structure of an injury from specific cognitive deficits.

## Introduction

Understanding networked and dynamic systems is of growing importance across the engineering, physical and biological sciences. Such systems are often composed of a diverse set of dynamic elements whose connectivity are prescribed by sparse and/or dense connections that are local and/or long-range in nature. Indeed, for many systems of interest, the diversity in connectivity and dynamics make it extremely challenging to characterize dynamics on a macroscopic network level.

Of great interest in biological settings is the fact that such complex networks often produce robust and low-dimensional functional responses to dynamic inputs. Indeed, the structure of their large connectivity graph can determine how the system operates as a whole [1, 2]. Neuronal networks, in particular, may encode key behavioral responses with low-dimensional patterns of activity, or population codes, as they generate functionality [3–8].

Unfortunately, all biological networks are susceptible to pathological and/or traumatic events that might compromise their performance. In neuronal settings, this may be induced by neurodegenerative diseases [9–11], concussions, traumatic brain injuries (TBI) [12–14] or aging. In this work, we extend a computational model to investigate behavioral impairments in the nematode *C. elegans* when the underlying neuronal network is damaged. Specifically, we consider how the low-dimensional population codes are compromised under the impact of an injury. Characterizing the resulting cognitive and behavioral deficits is a critical step in understanding the role of network architecture in producing robust functionality.

A hallmark feature of damaged neuronal networks is the extensive presence of Focal Axonal Swellings (FAS). FAS has been implicated in cognitive deficits arising from TBI and a variety of leading neurological disorders and neurodegenerative diseases. For instance, FAS is extensively observed in Alzheimer’s disease [10, 11], Creutzfeldt-Jakob’s disease [15], HIV dementia [16], Multiple Sclerosis [17, 18] and Parkinson’s disease [19]. Most concussions and traumatic brain injuries also lead to FAS or other morphological changes in axons [20–25]. Such dramatic changes in axon geometry may disrupt axonal transport [26, 27], and can potentially hinder the information encoded in neural spike train activity [28–30]. Injured axons thus provide an important diagnostic marker for the overwhelming variety of cognitive and behavioral deficits [9, 28, 31], in animals and humans [23, 32–34].

The massive size of human neuronal networks and their complex activity patterns make it difficult to directly relate neuronal network damage to specific behavioral deficits. *C. elegans*, in contrast, has only 302 neurons, and its stereotyped connectivity (i.e. the worm’s “Connectome”) is known [35]. This relatively small neuronal network generates a limited and tractable set of functional behaviors (see Table 1 of [36]), with much of its locomotion/crawling behavior approximately confined to five observable motor states related to forward and backward crawling, omega turns, head sweeps and brief pause states. Furthermore, these behaviors are well described as a superposition of only a few principal component body-shape modes [37]. The combination of a fully-resolved neuronal network and a tractable low-dimensional output space makes *C. elegans* an ideal model organism for studying the impact of network damage on behavioral deficits. Indeed, it is the only such neuronal network model currently available allowing for such a direct translational study of network damage (injury) to behavioral responses.

More precisely, computational models of *C. elegans* nervous system dynamics for the full or partial connectome successfully generate motorneuron outputs that can be related to behavior [38], allowing for interpretable outputs even without accounting for muscular, mechanical or environmental factors, e.g. [39]. We consider the model in [39], which applies a single-compartment membrane model to the full somatic connectome; neurons are approximated as passive linear units connected by linear gap junctions and nonlinear chemical synapses. Synaptic activation depends sigmoidally upon pre-synaptic voltage in equilibrium, and approaches this equilibrium value linearly in time. All neurons are approximated as identical, with order-of-magnitude parameter assignments, except for their connectivity data.

**Fig 1:**
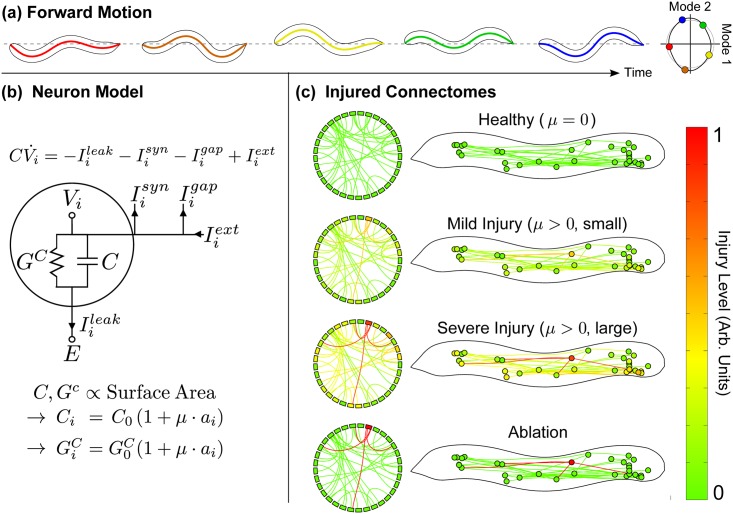
**(a)** In our model, stimulating PLM neurons drives two-mode motorneuron oscillations. We project all dynamics onto these modes. We map these projected dynamics onto the behavioral modes from [37] to reconstruct theoretical body motion. **(b)** We model neuron injury by scaling membrane capacitance and conductance with surface area. The relative swelling of neuron *i* is set by pulling its individual swelling level *a*_*i*_ randomly from a distribution, and scaling all swellings by an overall “injury amplitude” *μ*. **(c)** We refer to a particular set of *a*_*i*_ values as the same “injury”. Here we illustrate the same injury in three different regimes of *μ*. Compare the common experimental case of ablation, in which only one neuron is injured very severely (as opposed to our distributed injuries).

Fig 1(a) demonstrates a simulation of the putative forward crawling behavior identified in [39] within this model of *C. elegans* neural dynamics along with its projection onto principal component body-shape modes [37]. In this perspective, we understand forward crawling as corresponding to a limit cycle (i.e. a closed periodic trajectory) in the principal component space of simulated neural recordings. Extending this framework to *damaged* networks as in Fig 1(c) allow us to explore how axonal pathologies lead to impaired functionality and behavioral deficits. Even in our idealized injury simulations, the network’s impaired activity displayed significant variability. This highlights one of the most challenging aspects of the field: the need for effective *metrics* to distinguish different types of behavioral deficits. We propose such a criterium by using techniques borrowed from statistical shape analysis to quantify distortions in the main features of dynamical activity. This metric is shown to be related to the functional outcome of an injury. We further apply classification trees to our results to relate functional deficits to specific patterns of FAS. This leads to experimentally-testable predictions about the effects of neuronal network-damage to the crawling motion of *C. elegans* and potentially new avenues for clinical diagnostics. Indeed, our studies show that network damage leads to a diversity of dynamical/behavioral deficits.

## Results

### Low-Dimensional Signatures for Crawling Behavior

We investigate how network distributed FAS as illustrated in Fig 1(c) may affect its ability to generate desired responses to an input. Network features associated with behavioral outcomes are best understood in model organisms such as the *C. elegans* since it has a limited repertoire of functional responses that include forward and backward crawling, omega turns, head sweeps and brief pause states. Our focus in these studies will be on the behavior of forward crawling since a variety of experimental ablation studies have identified key neurons associated this functionality. For instance, stimulation of PLM neurons excites densely-connected interneurons, which in turn, activate motorneurons responsible for forward body motion [40]. Experimentally, optogenetic stimulation of the PLM neurons directly induces a forward motion response [41, 42].

Details of the underlying neurocircuitry were found by a series of ablation studies, where the functional role of a neuron is evaluated by disconnecting it from the network and observing behavioral deficits [39, 43]. The coordinated body motion of a crawling worm is well documented in videos and its postural dynamics were revealed by principal component analysis to consist of only a few dominant modes [37]. Specifically, the sinusoidal body-shape undulations which describe the worm’s forward motion is well-described by circular trajectories (limit cycles) on the phase-space of its first two principal components. An analogous mathematical form is present in the collective motorneuron dynamics following PLM stimulation [39].

This commonality suggests that observed behaviors do retain fundamental signatures of the underlying network dynamics. We show such a trajectory for (simulated) motorneuron responses to PLM excitation in Fig 1(a). This low-dimensional representation captures 99.3% of the total energy of the system, and can be artificially mapped to crawling body-shape modes. Although this mapping is still far from a mechanistic description of the worm’s coordinated body movement, we believe it captures important aspects of the crawling behavior. See the Methods section for details. Importantly, functional deficits of the *C. elegans* dynamics are understood as excursions/perturbations from the ideal limit cycle trajectory. Damaged networks will be shown to fail to produce the low-dimensional output codes necessary for generating the optimal forward crawling limit cycle.

### Modeling Injured Connectomic Dynamics

**Fig 2:**
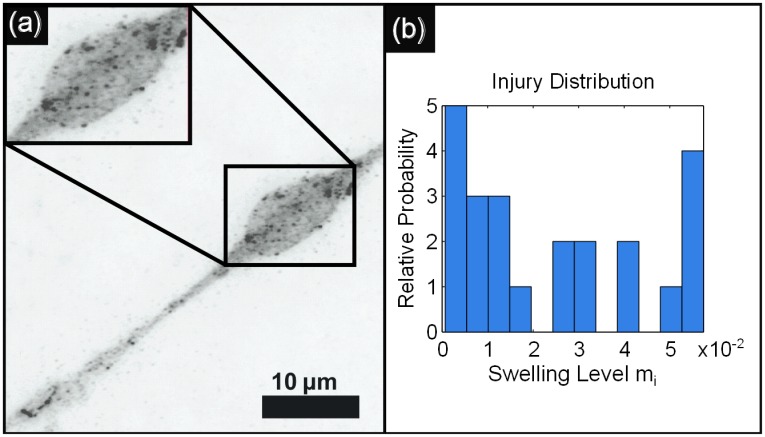
We draw swelling values from previously-measured experimental distributions. **(a)** Immunofluorescent image of an injured and swollen cortical neuron of a rat, from [20] (other examples of experimental neuron injury data can be found in [25, 26]). **(b)** It is equally simple to use any swelling distribution under our approach. Since such data does not yet to our knowledge exist for *C. elegans* specifically, we choose axonal swelling data from the optic nerve of Thy1-YFP-16 mice [25], taken 12hr. post-injury, from which we calculate the above probability distribution for neuron swelling levels *m*_*i*_.

The robustness of the dynamical signatures (population codes) associated with behavior are investigated in injured neuronal networks. Our injury statistics and FAS models are drawn from state-of-the-art biophysical experiments and observations of the distribution and size of FAS. Fig 2 shows prototypical FAS injuries from stretching [26] and TBI in the optic nerve of mice [25]. Fig 2(d) shows a histogram of the probability of injury and size of the FAS. These are used in our computational model [39].

In a simulated injury, we assign to each affected neuron an axonal swelling from the distribution in Fig 1(b). Values are scaled by an (overall) injury intensity parameter *μ*, such that

$$1+\mu \;\propto \;E\left[\frac{\text{swollen}\;\text{axon}\;\text{area}}{\text{healthy}\;\text{axon}\;\text{area}}\right]$$ (1)

**Fig 3:**
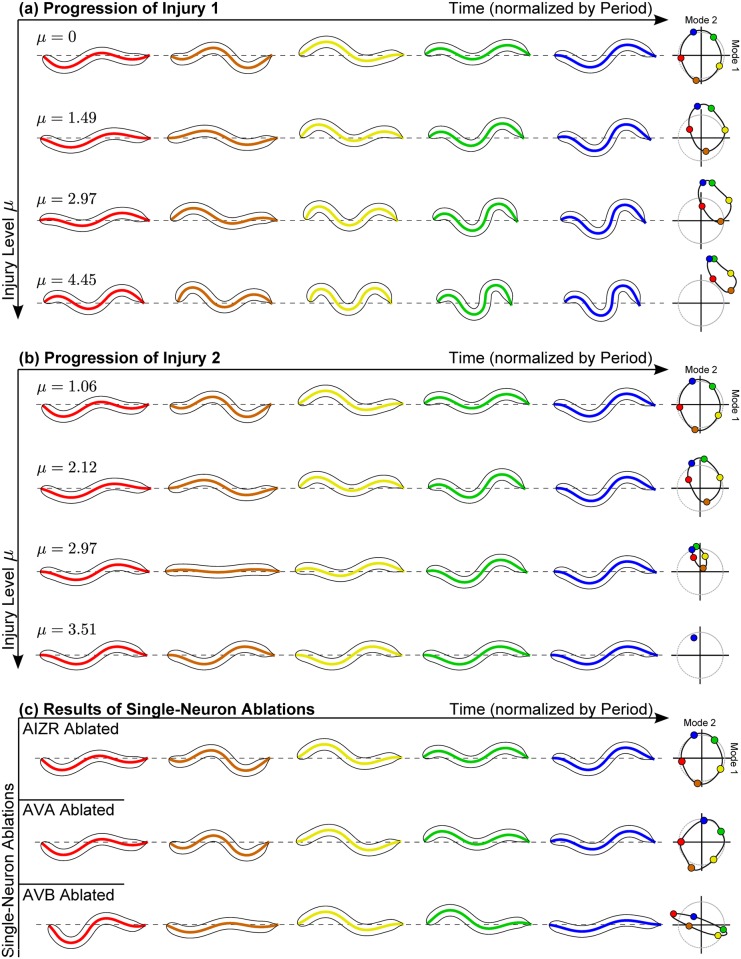
Response to PLM stimulation for two different distributed injuries in **(a)** and **(b)**. Resultant trajectories were mapped onto the two neural modes, which were then mapped onto the two behavioral modes to reconstruct theoretical body dynamics. Within each panel, injury amplitude *μ* is increased in subsequent rows. Different injuries yield qualitatively different injury progressions as *μ* is increased, and sufficiently high *μ* values lead to the cycle collapsing into a fixed point. **(c)** For comparison, consider the all-or-nothing injury effects of isolated single-neuron ablations. Experimentally, ablating AIZR inhibits reversals [44], ablating AVA causes backwards motion to become uncoordinated while preserving forward motion [43], and ablating AVB causes forward motion to become uncoordinated while preserving backwards motion [43]. Consistently, our simulated “forward motion” cycle is severely distorted by simulated AVB ablation but not by AIZR or AVA ablation, as shown.

Fig 1(c) exemplifies different injury settings: *μ* = 0 reproduces the original (uninjured) network, and lower/higher values of *μ* correspond to mild/severe injuries. The presence of axonal swellings ultimately distorts the forward-motion limit cycle dynamics. Fig 3 shows dynamical anomalies for different connectome injuries. Notice how they induce qualitatively different changes to the closed orbit regarding location, size and shape. Fig 3(c) reproduces the specific simulated ablations from [39], leading again to different dynamical effects.

A much larger ensemble of simulations (1,447 randomly-chosen injuries, as well as the code necessary to generate more) and their corresponding effects to fundamental low-dimensional structures are included in the Supporting Materials. Increasing values of *μ* typically shrink and shift the limit cycles within the plane. In all simulations, there was always a sufficiently high injury level in which

$$\mu^{*}=\{\text{injured limit cycle collapses into a stable fixed point}\}$$ (2)

This occurs for instance, in Fig 3(b) when *μ* = 3.80. Recent blast injury studies on *C. elegans* show that many of the nematodes display temporary paralysis before recovering to crawling behaviors [45]. We would suggest that during the peak of the FAS, the injury levels on many of the nematodes are above μ*, thus leading to a collapse of a limit cycle to a fixed point where no motion is possible, i.e. it is in a paralyzed state.

### Distinguishing Signatures of Different Behavioral Deficits

**Fig 4:**
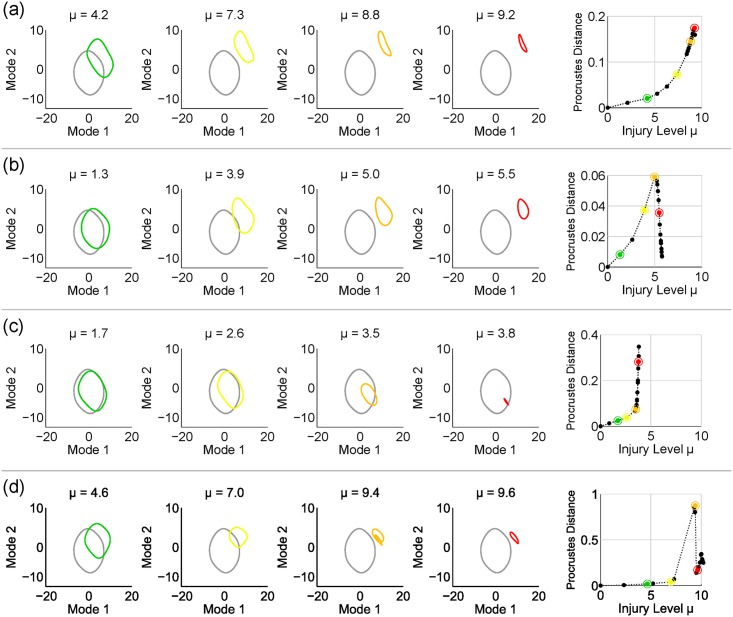
Each row (a),(b),(c) and (d) depicts projected neural responses for different injuries with increasing intensity *μ*. Distortion of each cycle is quantified via the Procrustes Distance (PD), which compares shapes ignoring translation, rotation or uniform scaling. The PD curves terminate when *μ* = μ*, level for which the cycle collapses into a fixed point (i.e. paralysis). Colored dots on the rightmost plots correspond to the same-colored limit cycles on the left plots.

Despite their common statistical distribution, randomly drawn injuries induce qualitatively different changes in the *shape* of the limit cycle. Additional distorted sets are shown in the rows of Fig 4 (along with 1,447 random-injury simulation sets in the Supporting Materials). Thus, random injuries of equitable strength can lead to significantly different behavioral deficits. Importantly, the deformation of the two-dimensional limit cycle can be used to characterize such functional differences. To distinguish dynamical signatures of potentially different functional deficits, we evaluate the *Procrustes Distance* (PD) between healthy and injured limit cycles. The PD is an important tool from statistical shape analysis to measure the similarity between two shapes after discounting effects due to translation, uniform scaling, or rotation. Fig 4 depicts PD values for pairs of healthy/injured limit cycles as a function of injury level *μ*. All curves are plotted until the injured limit cycle collapses into a fixed point (*μ* = μ*), and the colored dots in the rightmost plots correspond to the same-colored limit cycles on the left plots.

### Procrustes Distance Classes and Behavioral Dynamics

Recent experimental work which induced mild TBI in *C. elegans* found that increasing the number of shock waves to which the worm was exposed reduced the worm’s average speed and, in many cases, led to temporary paralysis [45]. The results of our simulations can be compared to these results:

- *Speed Reduction:* In our model, injury tends to slow the oscillation of the limit cycle. Specifically, the temporal frequency of the limit cycle was reduced by an average of 17% in the highly-injured interval (0.9μ*, μ*). However, a slower limit cycle frequency does not necessarily imply slower movement. As the frequency changes, so does the amplitude and shape of the limit cycle, and these will also affect the forward movement speed. Without a coupled mechanical model for the body movement of the worm and for the environment in which it moves, we are unable to calculate how these trajectory distortions affect forward movement speed.
- *Temporary Paralysis:* Simulated neural patterns are static at the fixed point when *μ* > μ*. Neglecting extra-connectomic effects (e.g. electrical coupling within muscles themselves) this may imply that the worm is not moving. The point at which the trajectory ends (the “endpoint”) should correspond to the fixed shape of the worm (note that this depends on the full-dimensional location of the point, not simply our plane projection). Thus the endpoint is posited to carry information about the paralyzed body shape of the worm, and we can investigate the relationship of the endpoint to both the PD curve and to the structure of the injury.

**Fig 5:**
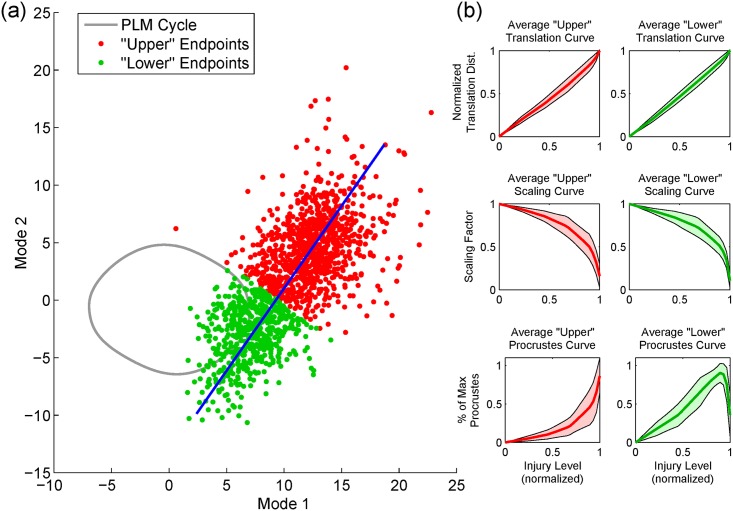
The Procrustes curves carry information about the functional outcome of an injury. **(a)** Plotted on our neural plane are the locations of the fixed points into which injured cycles eventually collapse (the “endpoints”), for sufficiently high *μ*. We classify the injury’s functional outcome by dividing the distribution along its major axis (into “upper” and “lower” endpoints). **(b)** The average Procrustes curve within each class is qualitatively distinct. This indicates that analyzing the shape of the Procrustes curve as *μ* increases may help predict the form of paralysis resulting from that specific injury. In contrast, the translation and scaling of the distorted cycles are monotonic and not distinct between classes.

In Fig 5 we plot the location of the fixed points into which limit cycles collapse (the “endpoints”, occurring at injury level *μ* = μ*). We consider the following question: does the location of this endpoint (and thus the behavioral outcome of the injury) relate to the PD curve, and does it relate to the structure of the injury itself? Towards this end, we construct two simple classes of behavioral outcomes: endpoints which end in either the “upper” or “lower” part of the distribution (for which we label the endpoints as red and green, respectively).

Panel (b) of Fig 5 shows the average PD curve for the two classes. They are qualitatively different: the average PD curve of “upper” endpoints is smoothly rising, whereas the average PD curve of “lower” endpoints has an extended declining region. Shown also are the average scaling factor and translation distance of the distorted cycles. Unlike the average PD curves, these change monotonically and are not distinct between classes. This suggests that the shape of the PD curve carries information about the functional outcome of the injury. We quantify this by fitting a classification tree to predict the endpoint class from the shape of the PD curve: this was found to predict endpoint class with a cross-validation error of 22.0%. By comparison, randomly shuffling the labels leads to nearly double the cross-validation error, with an average of (44.6 ± 1.4)%.

**Fig 6:**
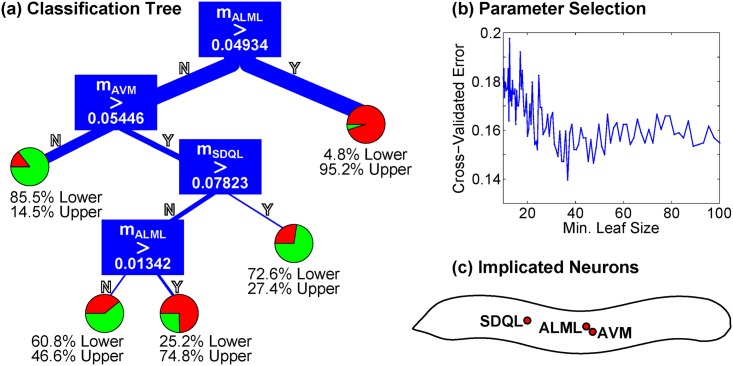
**(a)** Classification tree which predicts, for a given injury vector $\vec{m}$, whether the injured dynamics will fall into a “paralyzed” fixed point in the upper or lower part of the plane (as in Fig 5). The cross-validation accuracy exceeds 85%. **(b)** Cross-validation error as a function of minimum leaf size. We choose a minimum leaf size of 40. **(c)** The neurons implicated as most important for determining functional outcome of the injury, given these behavioral classes. A classification tree and implicated subset of neurons could be generated via this method for any feature of injured dynamics which we wished to explore.

Of even greater interest is any possible relationship between injury structure and behavioral output which could, given a specific pattern of distorted dynamics, make predictions about the class of neural injury. To this end, we fit a classification tree to predict the endpoint class from the injury. Fig 6 shows a classification tree which predicts endpoint class with a cross-validation error of only 14.6%. This is much less than the error from a random class, suggesting that we *can* meaningfully relate the structure of a specific injury to a specific behavioral outcome. Classification trees provide a highly interpretable and predictive method for making this connection, and make specific experimental predictions for the injuries corresponding to functional deficits.

## Methods

### Governing Equations

The dynamic model for the *C. elegans* connectome simulates its neuronal responses to stimuli with a number of simplifications aimed at keeping the number of parameters at a minimum: we use a fairly standard and simple single-compartment membrane equation, and treat all neurons as identical save for their connectivity. Many neurons in the network are nearly isopotential [46, 47], and it is a common and reasonable simplification to model neurons via single-compartment membrane equations, with membrane voltages as the state variables for each neuron. Given this, Wicks et al. constructed a single-compartment membrane model for neuron dynamics [48], which we later extended to incorporate connection data for the full somatic connectome [39]. We assume that the membrane voltage dynamics of neuron *i* is governed by:

$$C\dot{V_{i}}=-G^{c}\left(V_{i}-E_{cell}\right)-I_{i}^{Gap}\left(\vec{V}\right)-I_{i}^{Syn}\left(\vec{V}\right)+I_{i}^{Ext}$$ (3)

The parameter *C* represents the whole-cell membrane capacitance, *G*^*c*^ the membrane leakage conductance and *E*_*cell*_ the leakage potential of neuron *i*. The external input current is given by $I_{i}^{Ext}$. Note that this is, essentially, a fairly standard single-compartment membrane equation [49], and its governing equations are formally identical to that used by Wicks et al. [48] except for our use of the full somatic connectome, our simplifying parameter assumptions, and minor differences in the treatment of synaptic dynamics taken from [50].

In all simulations within this paper, we set $I_{i}^{Ext}$ to be constant for the PLM neuron pair and zero for all other neurons. This assures that densely connected interneurons will stimulate the motorneuron subcircuits responsible for forward crawling behavior. Neural interaction via gap junctions and synapses are modeled by the input currents $I_{i}^{Gap}\left(\vec{V}\right)$ (gap) and $I_{i}^{Syn}\left(\vec{V}\right)$ (synaptic). Their equations are given by:

$$I_{i}^{Gap}=\sum_{j}G_{ij}^{g}\left(V_{i}-V_{j}\right)$$ (4)

$$I_{i}^{Syn}=\sum_{j}G_{ij}^{s}s_{j}\left(V_{i}-E_{j}\right)$$ (5)

We treat gap junctions between neurons *i* and *j* as ohmic resistances with total conductivity $G_{ij}^{g}$. We assume that $I_{i}^{Syn}$ is also modulated by a synaptic activity variable *s*_*i*_, which represents the conductivity of synapses from neuron *i* as a fraction of their maximum conductivity. This is governed by:

$$\dot{s_{i}}=a_{r}\phi \left(v_{i};\beta ,V_{th}\right)\left(1-s_{i}\right)-a_{d}s_{i}$$ (6)

Here *a*_*r*_ and *a*_*d*_ correspond to rise and decay time, and *ϕ* is the sigmoid function *ϕ*(*v*_*i*_; *β*, *V*_*th*_) = 1/(1 + exp(−*β*(*V*_*i*_ − *V*_*th*_))). This form of sigmoidal activation is taken from [50]. Note that it can be shown (by setting $\dot{s}=0$) that, as in [48], the equilibrium value of *s*_*i*_ depends sigmoidally upon *V*_*i*_.

We keep all parameter values from [39] (see Table 1. The Connectome data, consisting of the number of gap junctions $N_{ij}^{g}$ and number of synaptic connections $N_{ij}^{s}$, are taken from Varshney et al. [35] (as available on WormAtlas [51]). As in that study, we consider only the 279 somatic neurons which make synaptic connections (excluding 20 pharyngeal neurons, and 3 neurons which make no synaptic connections).

**Table 1:** Parameter values assigned within the model.

Parameters (from [39])	Value
Uninjured Mem. Conductance *G*^*c*^	10pS
Uninjured Mem. Capacitance *C*_*H*_	1pF
Leakage Potential *E*_*c*_	−35mV
Gap Junction Conductivity *g*	100pS
Synaptic Conductivity *g*	100pS
Reversal Potential *E*_*j*_ (Excitatory)	0mV
Reversal Potential *E*_*j*_ (Inhibitory)	−45mV
Sigmoidal Width *β*	0.125mV^−1^
Synaptic Rise Constant *a*_*r*_	1 s^−1^
Synaptic Decay Constant *a*_*d*_	5 s^−1^

Each individual synapse and gap junction is assigned an equal conductivity of *g* = 100pS (such that $G_{ij}^{g}=g\cdot N_{ij}^{g}$ and $G_{ij}^{s}=g\cdot N_{ij}^{s}$). The values of cell membrane conductance and capacitance are affected by injuries, but in the uninjured case are set as equal for all neurons with values of *G*^*c*^ = 10pS and *C* = 1pF. Note that in uninjured simulations, all neurons are modeled as identical except for their connectivity and the assignment of them as excitatory or inhibitory (where *E*_*j*_ will have one of two values corresponding to these classes).

### Calculation of the PLM Response Plane

The model is valuable because it generates a low-dimensional neural proxy for behavioral responses. Specifically, constant stimulation of the tail-touch mechanosensory pair PLM creates a two-mode oscillatory limit cycle in the forward motion motorneurons [39]. This same dynamical signature was revealed in video analysis of the body-shape of the crawling worm [37]. Thus the model is consistent with the observed biophysics. Specifically, we calculate this plane by first simulating the forward-motion motorneuron membrane voltages (class DB,VB,DD,VD) in response to a PLM Input of *I*_*PLML*_, *I*_*PLMR*_ = 2 × 10^4^ Arb. Units for the uninjured model. We take time snapshots these membrane voltages ${\vec{V}}_{M}\left(t\right)$, collect them into a matrix *V*, and take that matrix’s singular value decomposition. That is:

$$V=\left[{\vec{V}}_{M}\left(t_{0}\right),{\vec{V}}_{M}\left(t_{1}\right)\ldots \right]=P\cdot \Sigma \cdot Q^{T}$$ (7)

where *P* and *Q* are unitary and *Σ* is diagonal. The columns of *P* are the principal orthogonal modes. As in [39], the first two of these modes (the first two columns of *P*) almost entirely capture the dynamics of the system within this subspace under constant PLM stimulation. Projection of the full-system dynamics onto this plane consists of projecting the system’s motorneuron dynamics onto these modes.

### Modeling FAS in Neuronal Network Simulations

Note that the single-compartment model which we employ ignores the spatial extent of neurons and specific location of each connection. Our simplified injury model therefore must treat injury as a whole-cell effect. Focal Axonal Swellings (FAS) increase the volume of an axon, which in turn, should alter the cell’s capacitance and leakage conductance within our model. If we approximate a neuron by a single cable of length *l* and constant cross-section *a*, we may assume that the circuit parameters will scale with the axonal volume, i.e.,

C∝a·l (8a)

$$G^{c}\propto a\cdot l$$ (8b)

When an axon swells, its healthy cross-sectional area *a*_*H*_ will increase to some swollen value *a*_*i*_ > *a*_*H*_. Thus we assume that the healthy values for capacitance *C* and conductance *G*^*c*^ will also change according to

$$C_{i}=C\cdot \left(a_{i}/a_{H}\right)=C\cdot \left(1+\mu \cdot m_{i}\right)\;$$ (9a)

$$G_{i}^{c}=G^{c}\cdot \left(a_{i}/a_{H}\right)=G^{c}\cdot \left(1+\mu \cdot m_{i}\right)\;$$ (9b)

We define the individual damage *m*_*i*_ to neuron *i* as proportional to the relative excess area from swelling, i.e., *m*_*i*_ ∝ (*a*_*i*_ − *a*_*H*_)/*a*_*H*_. Values of *m*_*i*_ are computed from the experimentally derived distributions in Fig 2. Specifically, we construct FAS from the axonal swelling data of Wang et al. [25], which used confocal microscopy to measure injury-induced swellings in the optic nerve of Thy1-YFP-16 mice. Taken together, these define an “injury vector” $\vec{m}$, which we then normalize to $\left|\right|\vec{m}{\left|\right|}_{2}=1$. After normalizing, the injury vector is then scaled by a *global injury intensity* defined as follows:

$$\mu =\frac{\langle a_{i}/a_{H}\rangle -1}{\left\langle m_{i}\right\rangle }$$ (10)

Mild traumatic brain injuries yield small values of *μ* indicating that the average area of swollen axons is small. Severe brain injuries yield high values of *μ*, indicating that large swellings are more common. We leave the PLM pair of neurons receiving input uninjured. All other neurons have their *m*_*i*_ values assigned from the experimental statistical distributions. The governing equation for an injured neuron is now

$$C\dot{V_{i}}=-G^{c}\left(V_{i}-E_{cell}\right)-\left(I_{i}^{Gap}\left(\vec{V}\right)+I_{i}^{Syn}\left(\vec{V}\right)\right)/\left(1+\mu \cdot m_{i}\right)$$ (11)

We can readily interpret the limiting cases: when *μ* ⋅ *m*_*i*_ = 0, the original governing equation is recovered, and thus *μ* = 0 corresponds to the healthy case. When *μ* ⋅ *m*_*i*_ is large, gap junction and synaptic currents have no effect. The neuron’s voltage decays exponentially to its leakage potential, effectively isolating it from the network.

Note that our random assignment of swelling values neglects any spatial structure of the injury. This could be easily modified by using a distribution which depends on the spatial location of the neuron. Furthermore, this is a very simple model for neuronal swelling, in keeping with our simple model for neurons. It necessarily neglects the actual geometry of swelling. The use of a multi-compartment model would enable this in future studies. Ultimately, there is currently limited biophysical evidence for making more sophisticated models. As such, we have tried to capitalize on as many biophysical observations as possible so as to make a model that is consistent with many of the key experimental observations.

### Numerical Simulations and Convergence Criteria

We use MATLAB (version R2013a) to solve the system of neuronal dynamical equations via Euler’s method, using a timestep of 10^−4^*s*. We consider an ensemble of 1,447 different types of injury (set of targeted neurons), for which the global intensity *μ* may vary from 0 (uninjured) to a critical value μ*. When the intensity exceeds μ* (found by a bisection algorithm), the limit cycle collapses to a fixed point. To obtain intermediate values, we perform five simulations linearly spaced throughout (0, 0.9μ*) and ten additional simulations throughout (0.9μ*, μ*).

We classify the resulting injured trajectories as a Fixed Point or a Periodic Orbit according to the following criteria:

1. *Fixed Point:* when the trajectory is always confined to a circular region of radius of 0.01 (about three orders of magnitude below the uninjured radius).
2. *Periodic Orbit:* when the trajectory escapes the circular region but re-enters it periodically.

Note that these criteria classify very small periodic orbits as fixed points, since their behaviors are very similar. The method may also classify sufficiently slow, long-timescale oscillatory transients as periodic. These tests ignore the first 5 seconds of simulation time (50,000 timesteps), chosen heuristically as a typical timescale of transient decay. After this initial wait, we check the criteria at the end of each subsequent 5 seconds of simulation time until convergence is detected. The results were not observed to be sensitive to the length of this interval.

### Artificial Mapping of Dynamical Signatures to Behavioral Modes

Stephens et al. [37] found that the forward crawling motion of *C. elegans* is well described by two principal component body-shape modes called *eigenworm* modes. When moving forward, the modes alternate within its phase space forming a limit cycle. Kunert et al. [39] also found a two-dimensional limit cycle, but for the collective motorneuron activity after PLM stimulation. They interpret this similar dynamical signature as a neuronal analog to the observed behavioral pattern.

To interpret the distorted neural activity caused by our simulated injuries, we construct a map from the neuronal activity plane onto the eigenworm plane. The body-shape modes were extracted from Figure 2(c) of [37]. We first calculate the optimal linear mapping of the healthy trajectory onto a circle (see Fig 3a). We then use this calibration for all other trajectories. This artificially translates anomalous neuronal responses to anomalous body motions. Our procedure has a number of limitations, for which we list a few:

1. The behavioral limit cycle in [37] is approximately circular, but the relative rotation between the two planes is unknown. This parameter could be inferred by observing the motion of injured or ablated worms.
2. It is unclear that the mapping would hold for injured worms, especially without accounting for body-shape modes (eigenworms) from impaired crawling behavior.
3. We consider only the first two (healthy) behavioral modes. Thus, lack of motion within this plane does not necessarily imply that the worm is not moving. The injured body-shape dynamics could evolve along different modes leaving no traces on the original two.

The lack of direct neuronal analogs for injured network modes limits our ability to interpret arbitrary impaired behavioral responses. Further computational work could also find neuronal proxies for additional behavioral modes so as to enable a more complete mapping. Recent work on blast injuries of worms [45] could potentially help extend the analysis by providing injured *eigenworm* mode projections.

### Procrustes Shape Analysis

*Procrustes Distance* (PD) measures the dissimilarity between shapes, and in our context, we wish to compare the *shape* of the trajectories of the healthy neural responses (circular orbits in the phase plane) with the distorted ones produced after simulated injuries. For that, we use the function procrustes.m from MATLAB’s Statistics and Machine Learning Toolbox. We collect *N* data points from each trajectory and annotate their (*x*, *y*) coordinates in a (2 × *N*) shape matrix *S*. The PD between two distinct shapes *S*_*A*_ and *S*_*B*_ is given by

$$PD=\min_{b,R,c}{∥S_{B}-b\cdot S_{A}\cdot R+\vec{c}∥}_{2}$$ (12)

In other words, it finds the optimal (2D) rotation matrix *R*, scaling factor *b* > 0, and translation vector $\vec{c}$ to minimize the sum of the squares of the distances between all points. Intuitively, it compares shapes discounting translation, rotation, or scaling. To calculate the PD curves as in Fig 4, we use the uninjured (*μ* = 0) limit cycle as our first shape *S*_*A*_. The second shape *S*_*B*_ is the limit cycle calculated for each injury at the indicated value of *μ*.

We pre-process the trajectories to extract data points only within a single period. Since injuries usually distort the trajectory length, we use MATLAB’s spline.m function to interpolate them and collect the same number of data points. Both limit cycles must also be phase-aligned, which we achieve by finding the phase that minimizes the Procrustes Distance.

### Classification of Deficient Behavioral Responses

We hypothesize that both the injury itself and the PD curves contain meaningful signatures of behavioral outcomes of a given injury. For example, there is always a critical injury level *μ* = μ* in which the injured response collapses into a fixed point. Our artificial map suggests that this endpoint location corresponds to the shape of a paralyzed worm. We thus wish to relate endpoint location to (1) the shape of the PD curve, and to (2) the injury vector $\vec{m}$.

For these purposes, we classified the endpoints simply by dividing the endpoint distribution along its major axis. Specifically, we take the distribution of endpoints in Fig 5, calculate the leading principal orthogonal mode (via taking the Singular Value Decomposition, as mentioned earlier), and classify the points by the value of their projection onto this mode (where we arbitrarily classify projection values ≥ −0.01 as the “upper plane” and < −0.01 as the “lower” plane). Given this definition, 63.2% of the points lie within the upper plane, and 36.8% lie in the lower plane. Note that all of the forthcoming analysis could be equally well applied to any other output feature, and so we choose this classification for its relative simplicity.

We calculate the average PD curve within each class. Since the PD curves may have a different number of points, we first pre-process them. Specifically, we normalize the maximum *μ* and Procrustes Distance to 1 for all curves, and then interpolate them using MATLAB’s spline.m such that all curves have the same number of points. We then simply take the average and standard deviation to obtain the average curves shown within Fig 5. This figure also plots the average scaling and translation curves as a function of injury level, for each class. Scaling factors (i.e. the factor by which the size of the distorted limit cycle has decreased from the original cycle) are given as an output of MATLAB’s procrustes.m as used above. Translation distance is found by calculating the location of the mean of each distorted cycle, and then calculating the distance by which this mean is displaced from the origin. These curves are then normalized, interpolated and averaged, yielding the average curves in Fig 5. Note that, unlike the PD curves, translation and scaling are monotonic and not distinct between classes, and thus they do not carry the same information about the functional outcome of the injury.

### Classification Trees

We use the ClassificationTree class from MATLAB’s Statistics Toolbox (version R2013a). Fitting and cross-validation are performed using the included methods ClassificationTree.fit and kfoldLoss with default settings (10 folds). The minimum leaf size was set by calculating cross-validation error over a range of minimum leaf sizes (see Fig 6b). For both PD curves and Injuries, cross-validation errors are optimal at a minimum leaf size of around 40. We use this minimum leaf size for all fits.

The classification tree that uses normalized PD Curve Shapes to predict the endpoint class yield a cross-validation error of 22.0%. We can compare this to the random case (i.e. the case where PD Curve Shape has no relationship to the class) by repeating this analysis with randomly shuffled class labels. For 100 trials with randomly-shuffled labels, the observed cross-validation error was 43.8 ± 1.4%. Injury vectors were also used to fit classification trees for predicting endpoint classes (see Fig 6). The cross-validation error of 14.6% was significantly lower in this case, while the randomly-shuffled labels analysis returned a error of 44.6 ± 1.3% (consistent with the random error above). In both cases we observe that the cross-validation error is far below what we would expect if the data had no relation to the classes.

Thus we can predict (with cross-validated accuracy exceeding 85%) the region into which the endpoint will fall given a specific injury. Moreover, the classification tree in Fig 6 is very simple to interpret and depends on only three neurons: ALML, AVM and SDQL. As per WormAtlas [51], all three of these neurons have sensory functions (ALML and AVM are mechanosensory; SDQL is an interneuron but is oxygen-sensing).

## Discussion

This study introduces a tractable framework for analyzing how biophysically-inspired injuries distributed across a physical neuronal network induce behavioral deficits. The specific injuries we consider arise from FAS which has been implicated in most leading neurodegenerative diseases, aging and TBI. By identifying low-dimensional population codes within our model which correspond to a known behavior, a proxy metric for cognitive deficit can be constructed. Specifically, limit cycles in our dominant features serve as a neural proxy for actions such as forward motion in the *C. elegans*. Such trajectories can be artificially mapped onto experimental body-shape modes, and suggests a behavioral interpretation of the distorted limit cycles resulting from an injury. Our analysis also suggests that there is a diversity of functional deficits that arise from the same level of injury on a connectomic network.

The ability to provide a theoretical understanding of functional, cognitive and behavioral deficits due to connectomic injuries is a the forefront of TBI and neurodegenerative disease studies. Both have an enormous societal impact and implications. Specifically, TBI is annually responsible for millions of hospitalizations [52, 53], with reports estimating that 57 million people worldwide experienced some form of TBI [14]. It was also manifest in around 15% of all veterans of the Iraq and Afghanistan wars, with blast injuries being the signature wound of these conflicts [14, 53]. Numerous studies show that even mild concussions, if induced repeatedly, can lead to permanent brain damage; the issue is constantly debated in the sports media, but especially in football [54]. Neurodegeneration affects orders of magnitude more people than TBI through diseases such as Alzheimer’s disease [10, 11], Creutzfeldt-Jakob’s disease [15], HIV dementia [16], Multiple Sclerosis [17, 18] and Parkinson’s disease [19]. Thus, any study that can help understand how FAS compromises cognitive function is of great value.

### Diagnostic Tools

Simulated injuries distort dynamical signatures in the network’s activity, such as limit cycles. Our Procrustes Distance metric quantifies how much the shape of the limit cycle is distorted, compared to the healthy cycle. Our results indicate that as different injuries evolve, this metric follows qualitatively different trends (as in Fig 4). In all trials, a sufficiently high injury level *μ* = μ* collapses the limit cycle into a stable fixed point. The shape of the PD curve helps inform the location of this fixed point (as in Fig 5). This suggests that the shape of the PD curve, as the injury evolves, may help predict the eventual behavioral outcome (e.g., the body shape the worm will assume during temporary paralysis). Thus we have prescribed a method to monitor the dynamics of the injured worm and the implications of the injury as it evolves. Finally, our classification trees divides neural injuries into two distinct classes of functional outcomes (i.e. endpoints in the “lower” or “upper” portions of the distribution). Its cross-validation predictive accuracy is over 85% and implicates only three specific neurons (ALML, AVM, and SDQL). This relationship between injury structure and behavioral outcome is simple, interpretable and testable. Such trees can be fit for arbitrary injured behaviors and could be used more broadly for any given model of injured full-Connectome dynamics.

The metrics and methods described in this work can potentially be used to construct diagnostic tools capable of identifying a variety of cognitive deficits. Moreover, the severity of a TBI injury and/or neurodegenerative disease can be quantified by measuring its metric distance from the normal/healthy performance. Our work gives clear mathematical tools capable of formulating such diagnostic tools for assessing injuries and functional deficits.

### Limitations

The present study has many limitations, many due to the lack of biophysical evidence required to build better models. For example, though we treat all neurons as identical passive, linear units, it is known experimentally that different neurons appear to exhibit different behaviors (for example, some neurons appear to be functionally bistable [55] and could be modeled as such, as in [56]). We predict the results of injuries only on the two “forward-motion” motorneuron modes, ignoring other modes potentially associated with impaired behaviors. Furthermore, the exact mapping of our motorneuron voltage modes onto these body-shape modes is ambiguous. The model lacks muscles and body features of the worm which limits our ability to make more general predictions. We also neglect external feedback mechanisms required for sustained and spontaneous forward motion, and assume that tail-touch neurons are constantly stimulated. It is uncertain how such feedback mechanisms would alter the trajectory. The order-of-magnitude parameter estimates of our model parameters also make direct quantitative comparisons difficult.

### Future Work

We believe the merit of this study lies not so much on the specific results presented, but on the new directions and methodologies it opens for future work. In fact, computational and experimental studies on the effects of network injury are still at their infancy for *C. elegans* and other models. Many limitations of this work could be overcome with a more detailed model for the *C. elegans* neuronal network both before and after injury. Coupling this with an external, mechanical model would allow for more general predictions. This could be accomplished with simplified mechanical models for locomotion (such as in [56]) or with more complete, future “in-silica” models such as OpenWorm [57]. The development of such models, which do not ignore the spatial extent and shape of neurons, would allow for the study of the effects of injuring individual connections, or the effect of injuring individual neurons non-homogeneously. This study suggests that such modeling work should also consider how to model neural injuries, after which our analysis techniques could be applied directly.

Experimental studies would not only test our model, but also in, in conjunction with our work, provide a new testbed for models of injured connectomic dynamics. Our Procrustes Distance metric, shown here to carry information about the eventual outcome of an injury, may also be useful in the real-time analysis of injury progression. Thus our study provides a way forward in monitoring behavioral outcomes of injured networks.

Ultimately at present, limitations in biophysical measurements and neural recordings make it extremely difficult to identify more sophisticated underlying mechanisms responsible for dysfunctions in neural networks, especially when circuits display intrinsically complex behavior and functional activity. We believe the rapid advancement of recording technologies in neuroscience will significantly help refine the model presented here.

Given that the modeling of neuronal networks is one of the most vibrant fields of computational neuroscience [49, 58, 59], our contribution provides a comprehensive study of how the effects attributed to FAS jeopardize the network functionality, opening new possibilities and objectives for the study of network architectures.

## Supporting Information

S1 Source Code Simulation source code. A .zip file of the MATLAB code used to both conduct simulations for a random injury, calculate the PD curves, and visualize the injured trajectories.
(ZIP)

S1 Figures Figures for 1,447 injuries. Figures similar to the rows of Fig 4, for all 1,447 trials conducted.
(ZIP)

S1 Videos Visualization videos. Videos of injured trajectories mapped onto body shape modes. We include three examples of distorted trajectories along with the healthy trajectory. Similar videos can be created with the included source code.
(ZIP)

S1 Initial Conditions Injury distributions for 1,447 injuries. MATLAB data file containing injury distributions for all 1,447 trials. Can be used in conjunction with the above source code to recreate the injuries simulated within this manuscript.
(MAT)
