# Supplementary figures and images for: Functionality and Robustness of Injured Connectomic Dynamics in C. elegans: Linking Behavioral Deficits to Neural Circuit Damage (part 1 of 3)
Source: PLoS Comput Biol. 2017 Jan 5;13(1):e1005261. doi: 10.1371/journal.pcbi.1005261 (PMC5215891; doi:10.1371/journal.pcbi.1005261)

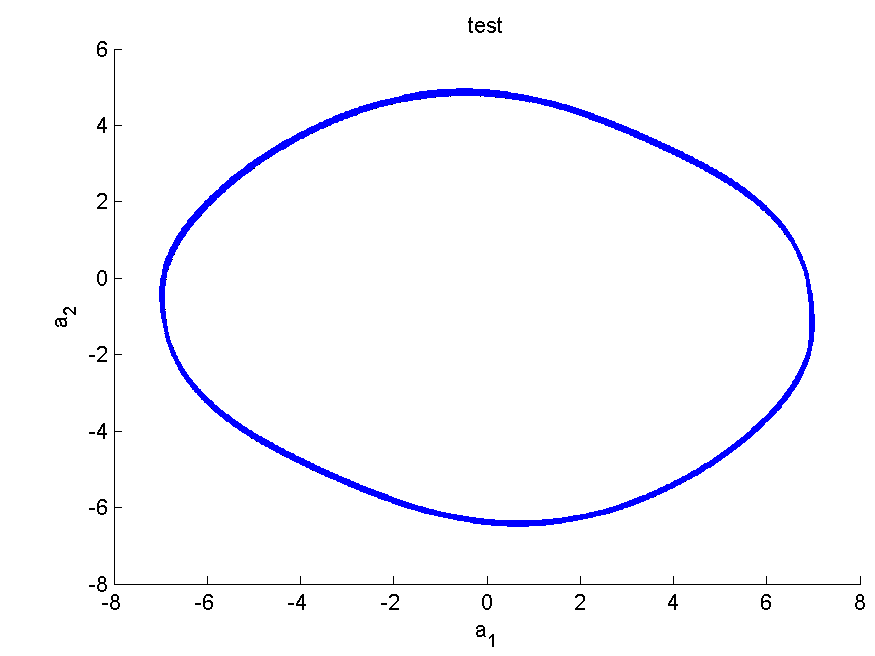

Supplement: S1 Source Code — A .zip file of the MATLAB code used to both conduct simulations for a random injury, calculate the PD curves, and visualize the injured trajectories. (ZIP) [file pcbi.1005261.s001.zip › subfunctions_simulation/testtest.png]

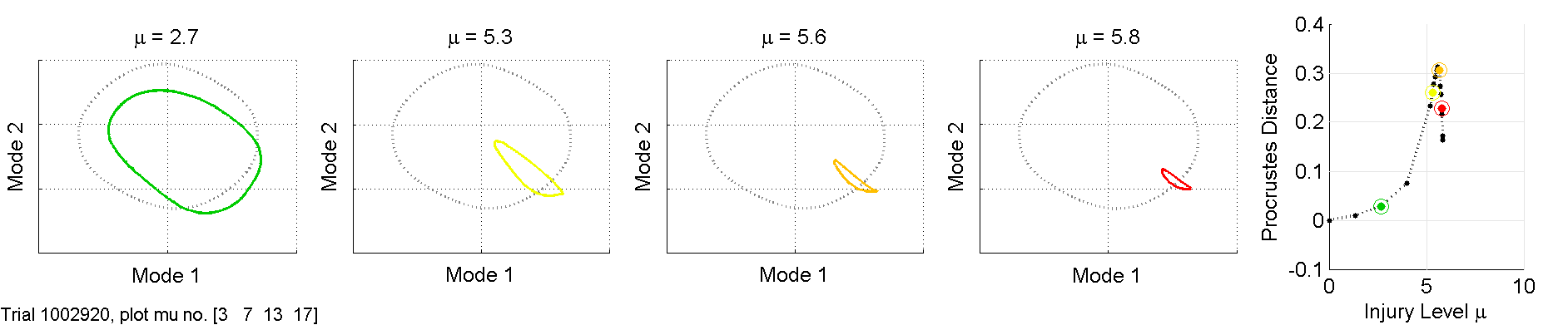

Supplement: S1 Figures — Figures similar to the rows of Fig 4, for all 1,447 trials conducted. (ZIP) [file pcbi.1005261.s002.zip › 1002920.png]

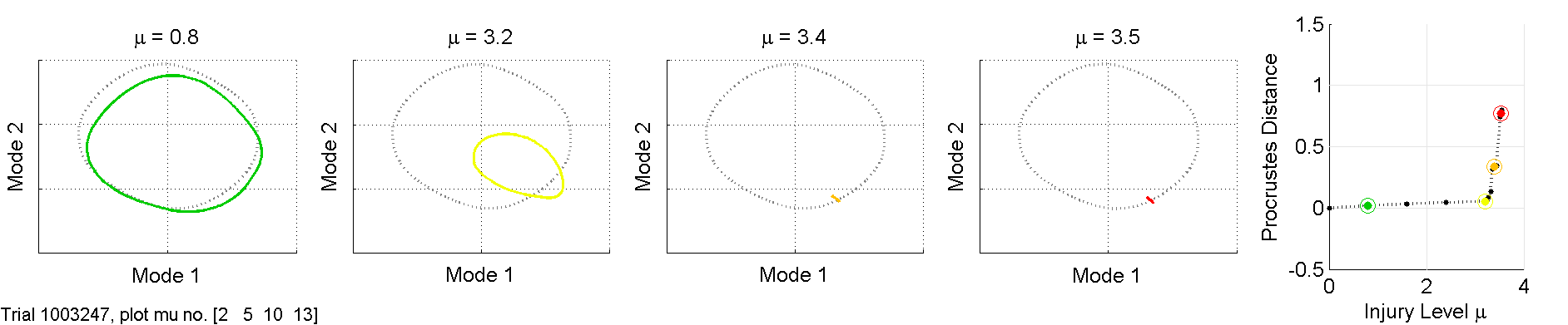

Supplement: S1 Figures — Figures similar to the rows of Fig 4, for all 1,447 trials conducted. (ZIP) [file pcbi.1005261.s002.zip › 1003247.png]

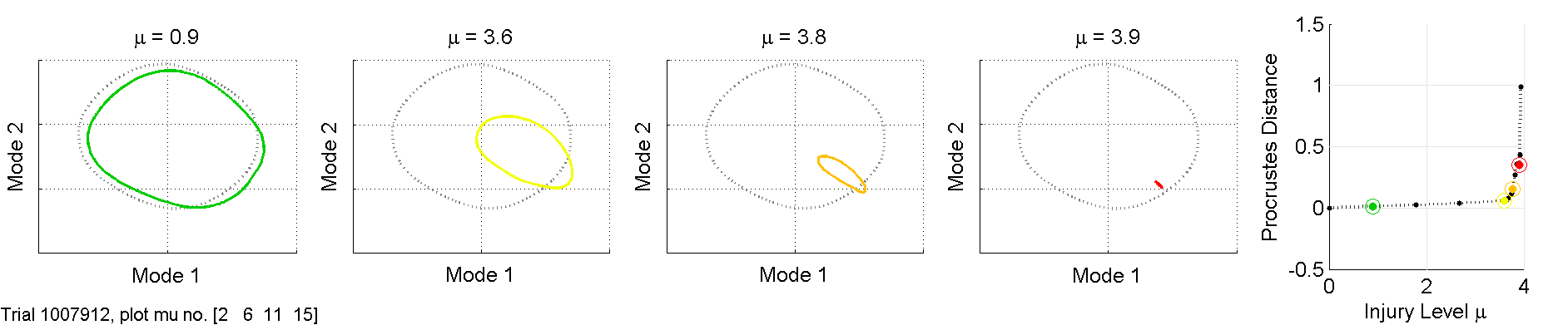

Supplement: S1 Figures — Figures similar to the rows of Fig 4, for all 1,447 trials conducted. (ZIP) [file pcbi.1005261.s002.zip › 1007912.png]

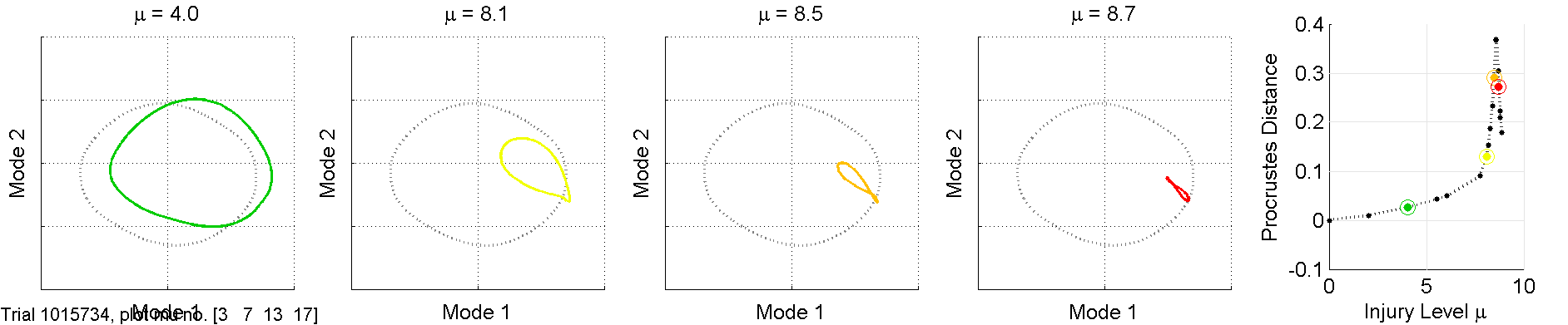

Supplement: S1 Figures — Figures similar to the rows of Fig 4, for all 1,447 trials conducted. (ZIP) [file pcbi.1005261.s002.zip › 1015734.png]

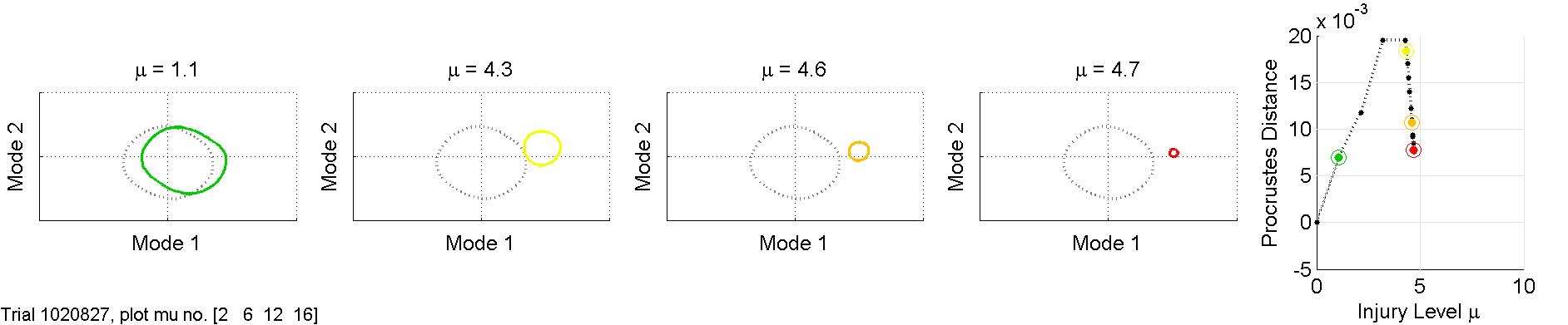

Supplement: S1 Figures — Figures similar to the rows of Fig 4, for all 1,447 trials conducted. (ZIP) [file pcbi.1005261.s002.zip › 1020827.png]

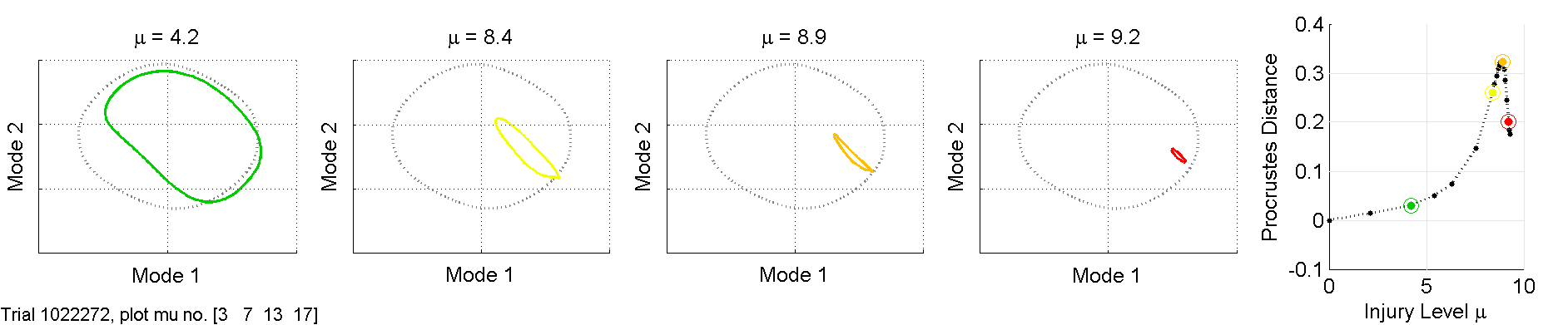

Supplement: S1 Figures — Figures similar to the rows of Fig 4, for all 1,447 trials conducted. (ZIP) [file pcbi.1005261.s002.zip › 1022272.png]

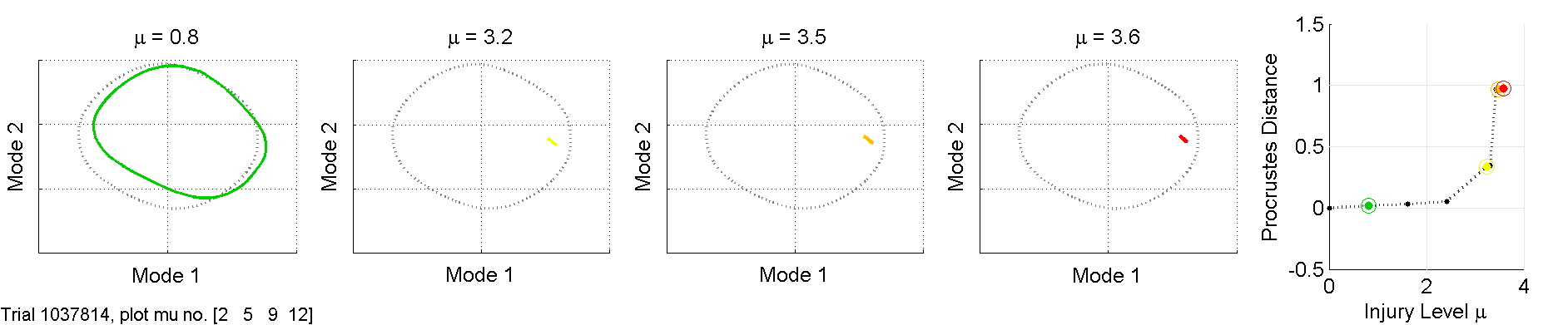

Supplement: S1 Figures — Figures similar to the rows of Fig 4, for all 1,447 trials conducted. (ZIP) [file pcbi.1005261.s002.zip › 1037814.png]

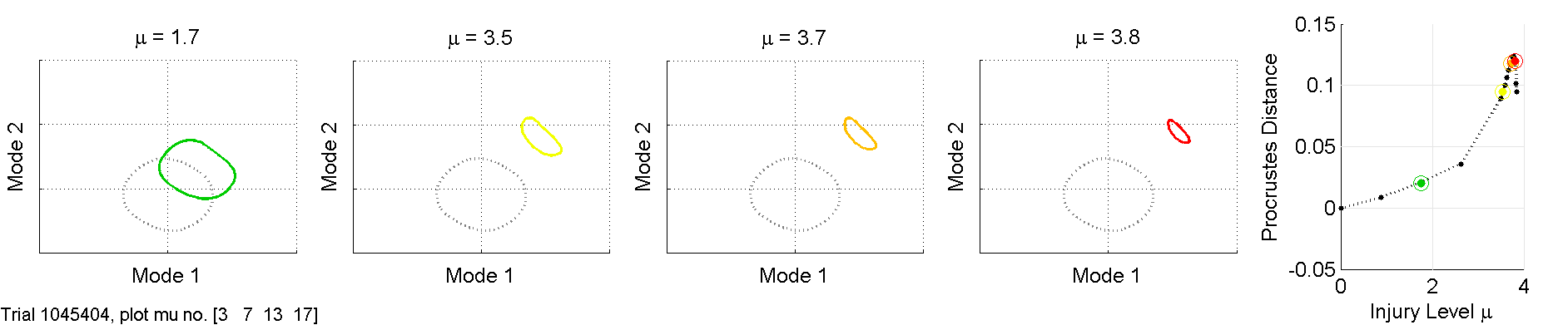

Supplement: S1 Figures — Figures similar to the rows of Fig 4, for all 1,447 trials conducted. (ZIP) [file pcbi.1005261.s002.zip › 1045404.png]

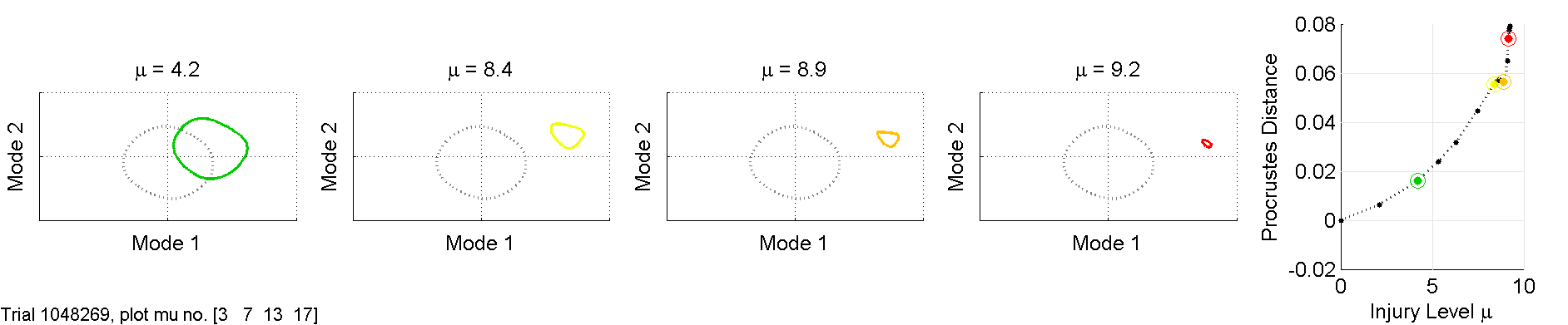

Supplement: S1 Figures — Figures similar to the rows of Fig 4, for all 1,447 trials conducted. (ZIP) [file pcbi.1005261.s002.zip › 1048269.png]

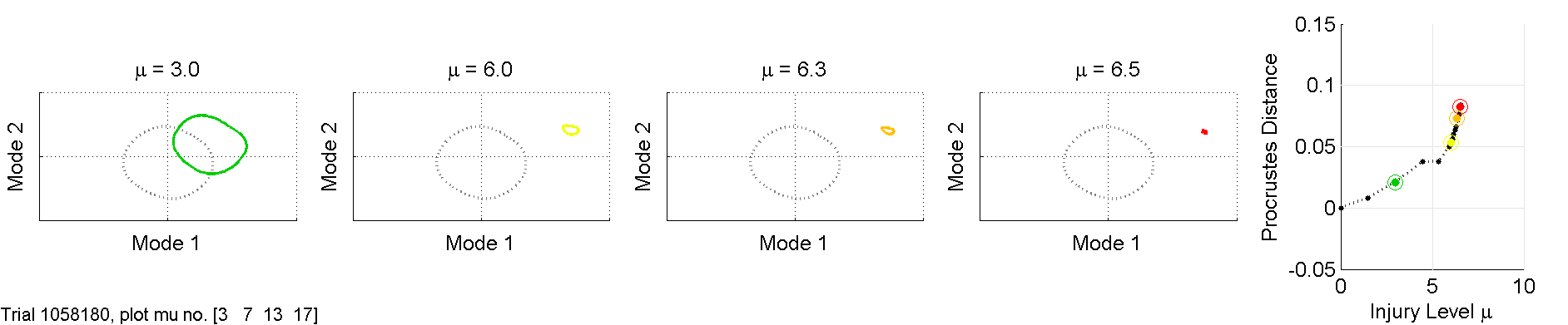

Supplement: S1 Figures — Figures similar to the rows of Fig 4, for all 1,447 trials conducted. (ZIP) [file pcbi.1005261.s002.zip › 1058180.png]

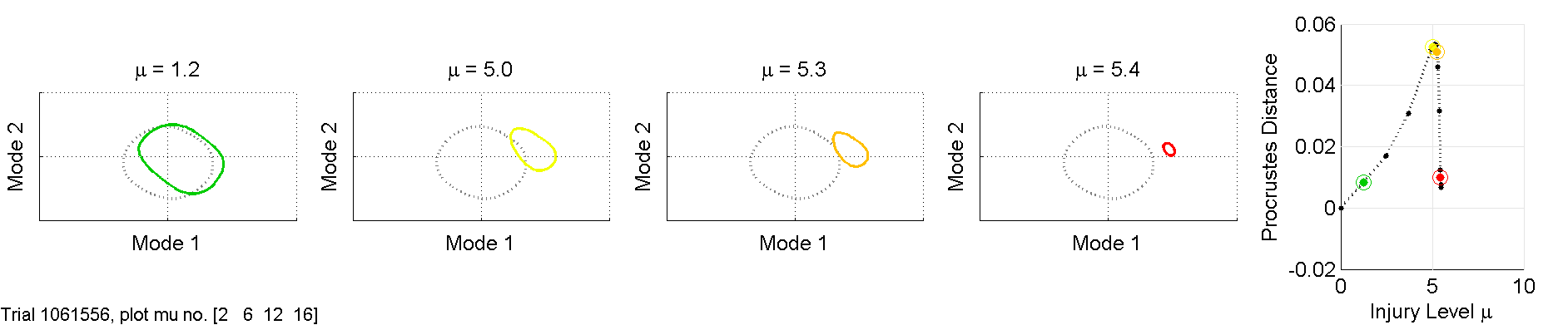

Supplement: S1 Figures — Figures similar to the rows of Fig 4, for all 1,447 trials conducted. (ZIP) [file pcbi.1005261.s002.zip › 1061556.png]

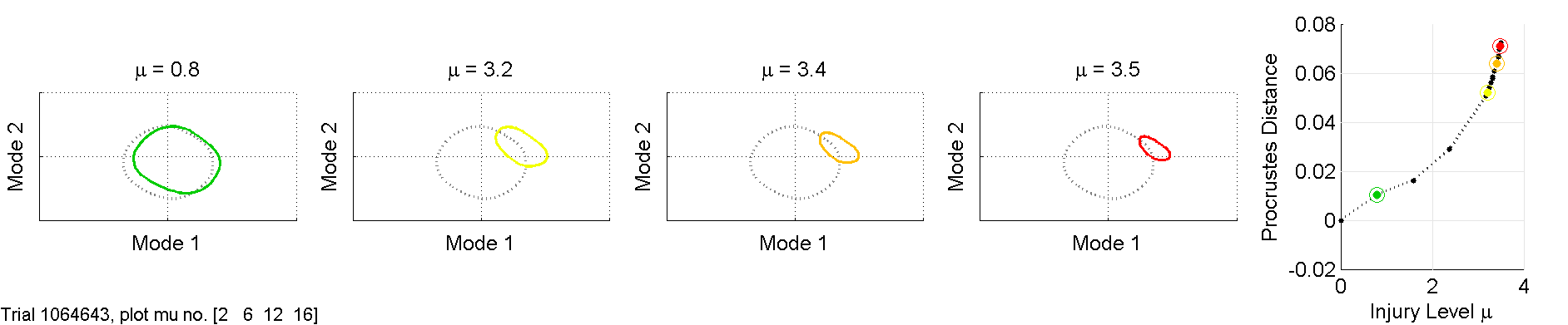

Supplement: S1 Figures — Figures similar to the rows of Fig 4, for all 1,447 trials conducted. (ZIP) [file pcbi.1005261.s002.zip › 1064643.png]

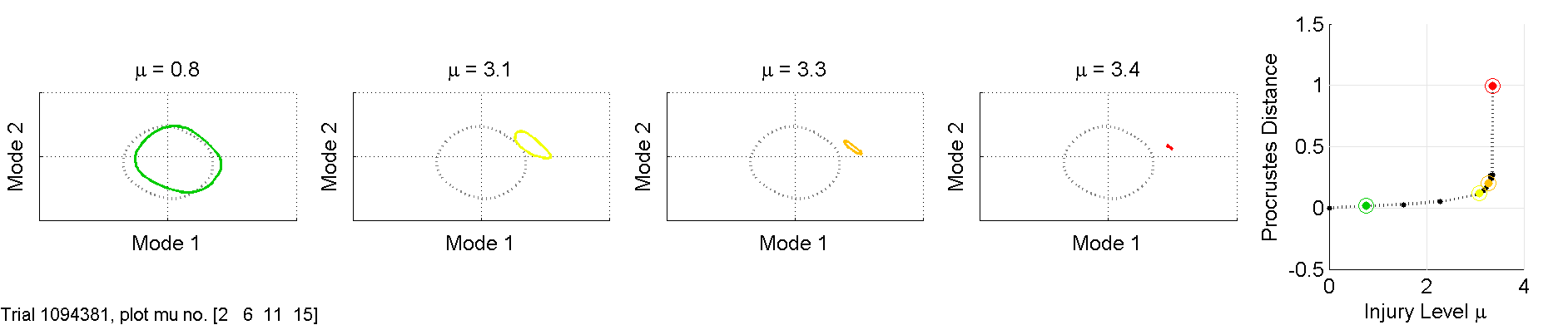

Supplement: S1 Figures — Figures similar to the rows of Fig 4, for all 1,447 trials conducted. (ZIP) [file pcbi.1005261.s002.zip › 1094381.png]

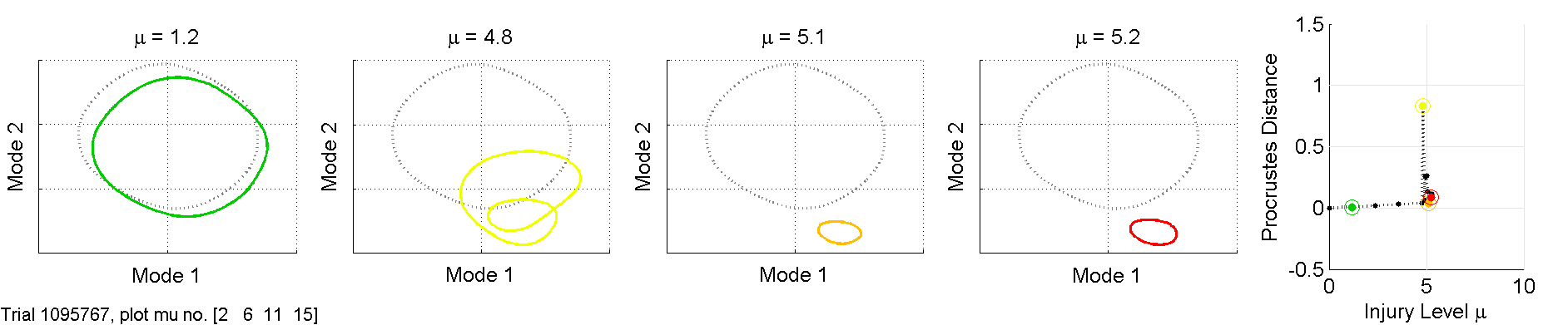

Supplement: S1 Figures — Figures similar to the rows of Fig 4, for all 1,447 trials conducted. (ZIP) [file pcbi.1005261.s002.zip › 1095767.png]

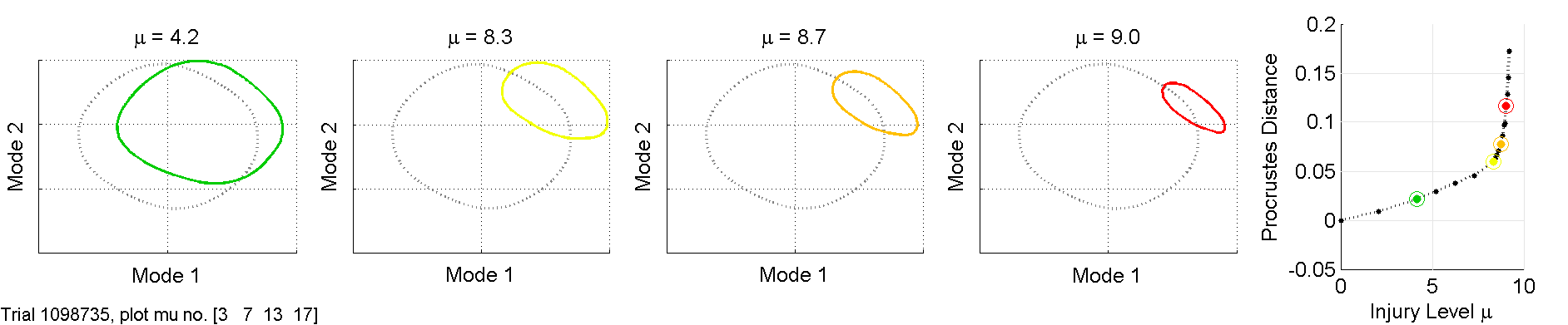

Supplement: S1 Figures — Figures similar to the rows of Fig 4, for all 1,447 trials conducted. (ZIP) [file pcbi.1005261.s002.zip › 1098735.png]

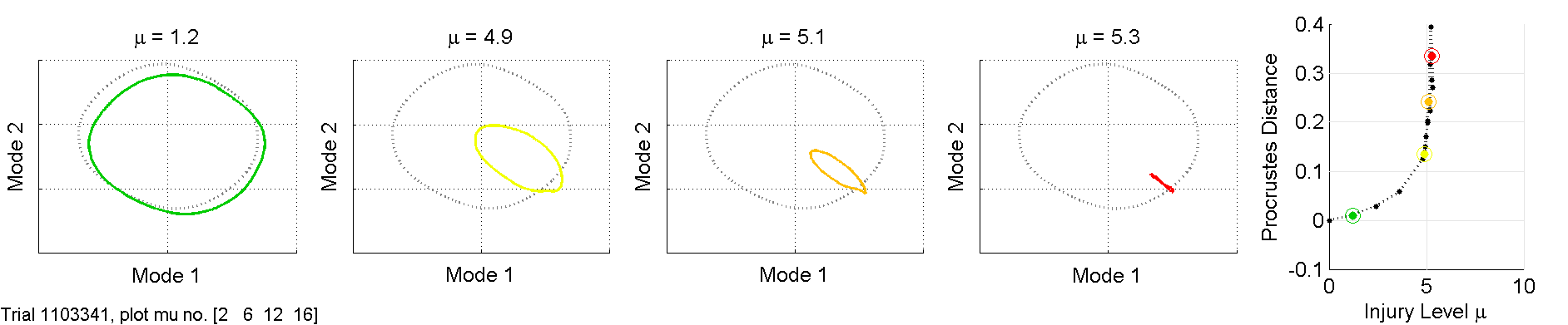

Supplement: S1 Figures — Figures similar to the rows of Fig 4, for all 1,447 trials conducted. (ZIP) [file pcbi.1005261.s002.zip › 1103341.png]

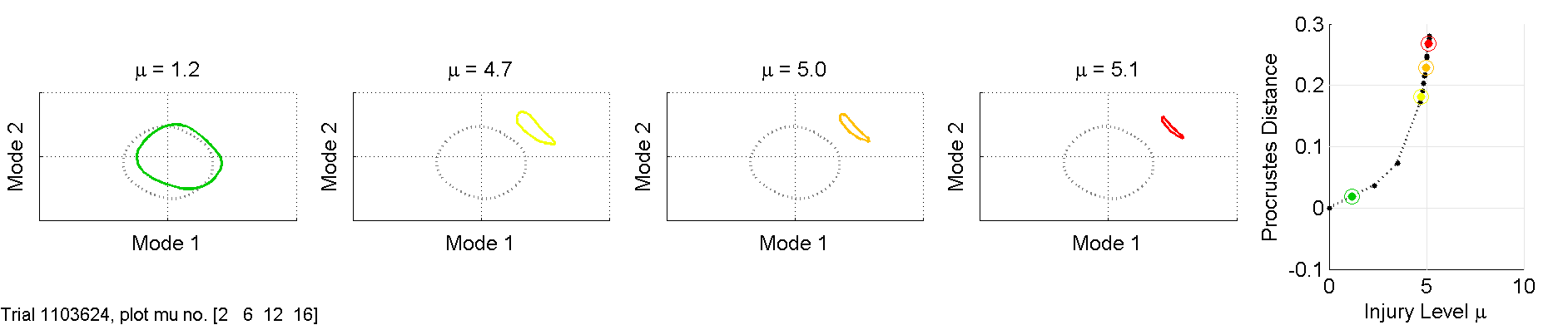

Supplement: S1 Figures — Figures similar to the rows of Fig 4, for all 1,447 trials conducted. (ZIP) [file pcbi.1005261.s002.zip › 1103624.png]

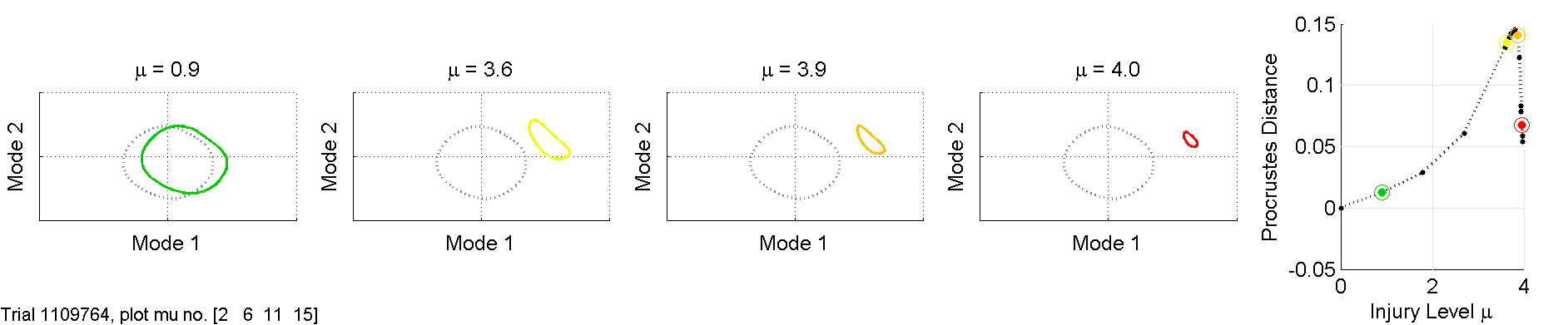

Supplement: S1 Figures — Figures similar to the rows of Fig 4, for all 1,447 trials conducted. (ZIP) [file pcbi.1005261.s002.zip › 1109764.png]

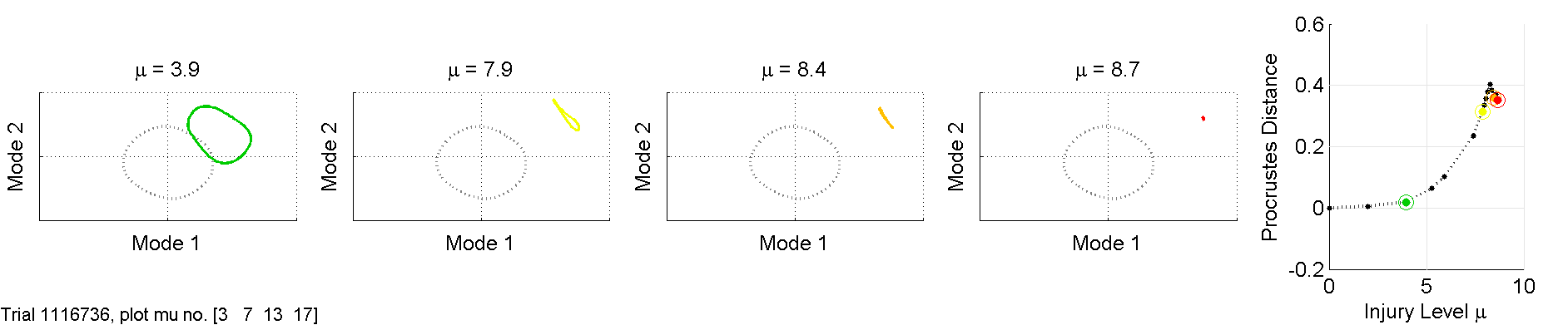

Supplement: S1 Figures — Figures similar to the rows of Fig 4, for all 1,447 trials conducted. (ZIP) [file pcbi.1005261.s002.zip › 1116736.png]

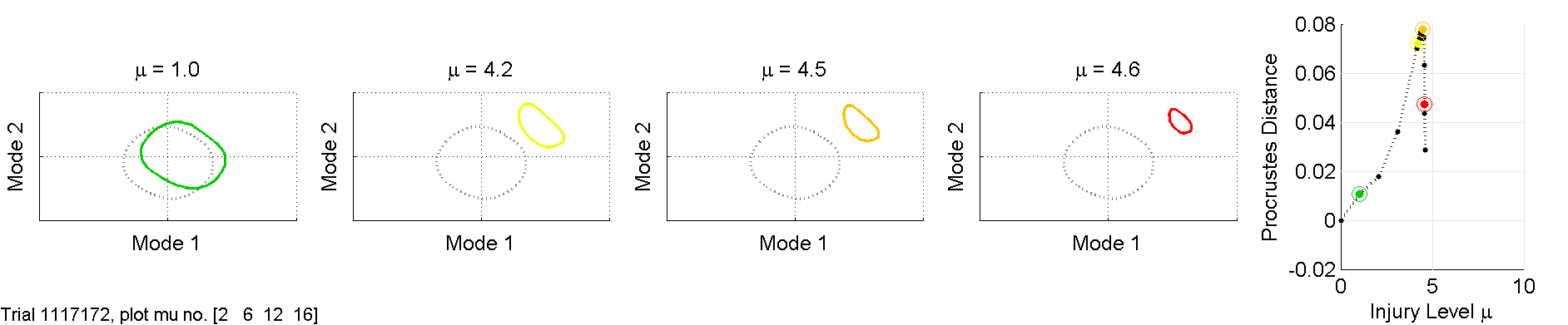

Supplement: S1 Figures — Figures similar to the rows of Fig 4, for all 1,447 trials conducted. (ZIP) [file pcbi.1005261.s002.zip › 1117172.png]

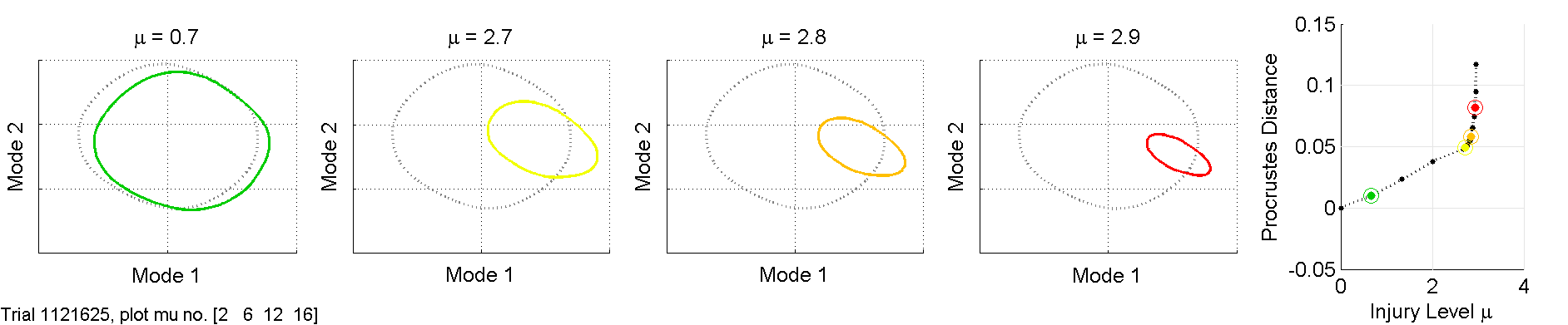

Supplement: S1 Figures — Figures similar to the rows of Fig 4, for all 1,447 trials conducted. (ZIP) [file pcbi.1005261.s002.zip › 1121625.png]

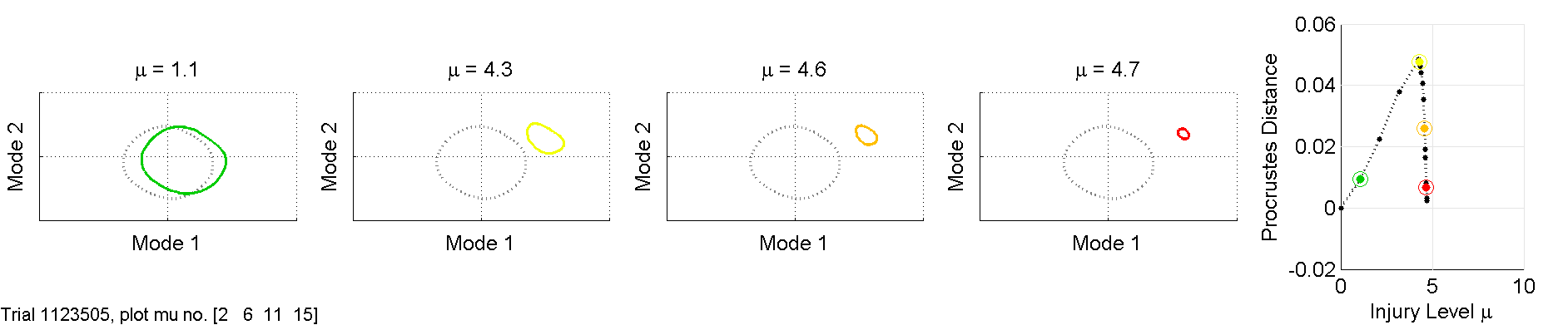

Supplement: S1 Figures — Figures similar to the rows of Fig 4, for all 1,447 trials conducted. (ZIP) [file pcbi.1005261.s002.zip › 1123505.png]

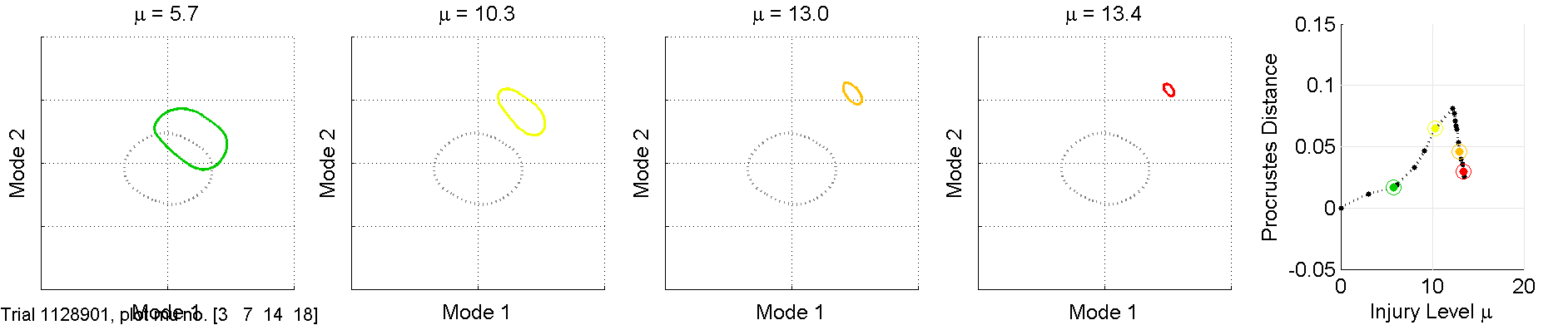

Supplement: S1 Figures — Figures similar to the rows of Fig 4, for all 1,447 trials conducted. (ZIP) [file pcbi.1005261.s002.zip › 1128901.png]

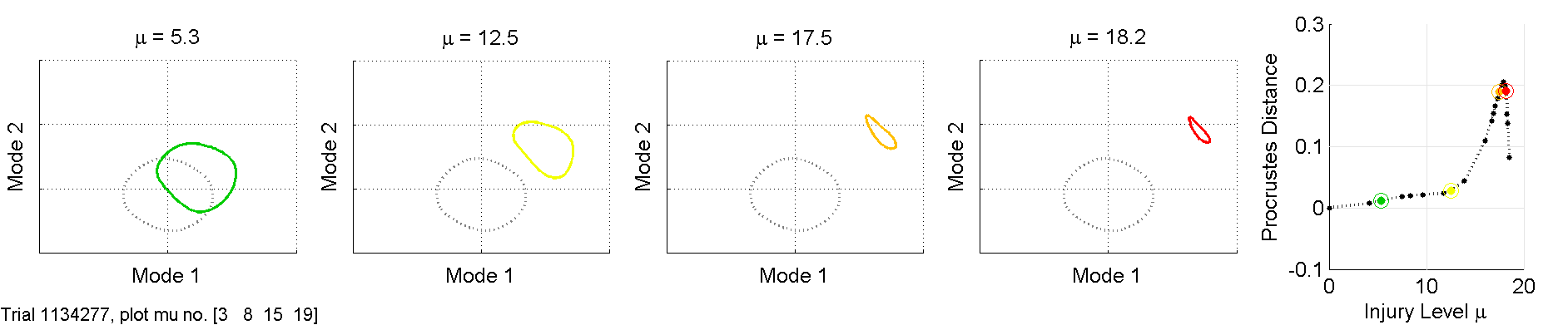

Supplement: S1 Figures — Figures similar to the rows of Fig 4, for all 1,447 trials conducted. (ZIP) [file pcbi.1005261.s002.zip › 1134277.png]

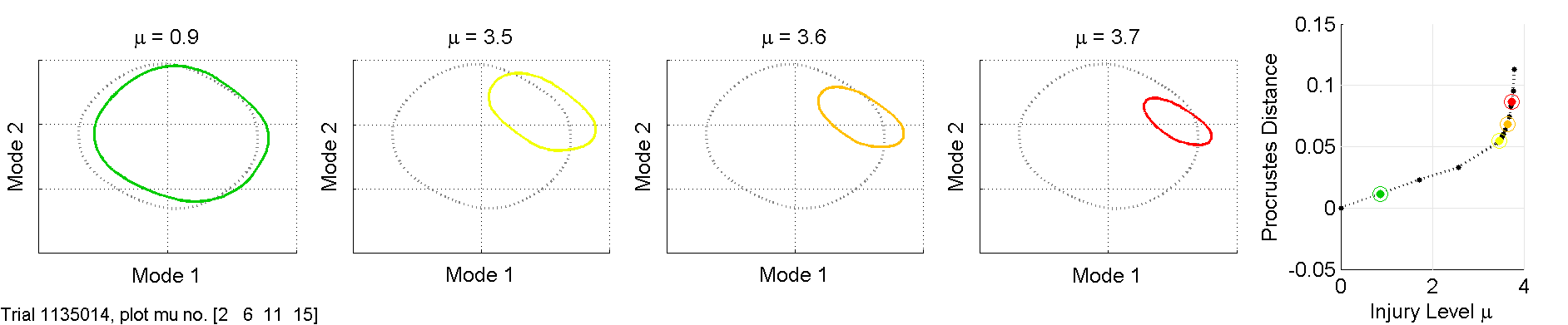

Supplement: S1 Figures — Figures similar to the rows of Fig 4, for all 1,447 trials conducted. (ZIP) [file pcbi.1005261.s002.zip › 1135014.png]

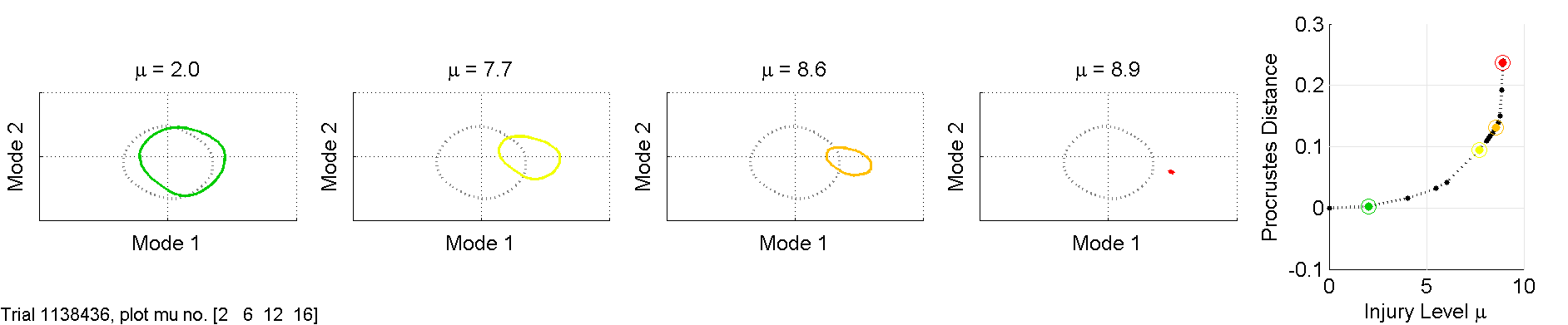

Supplement: S1 Figures — Figures similar to the rows of Fig 4, for all 1,447 trials conducted. (ZIP) [file pcbi.1005261.s002.zip › 1138436.png]

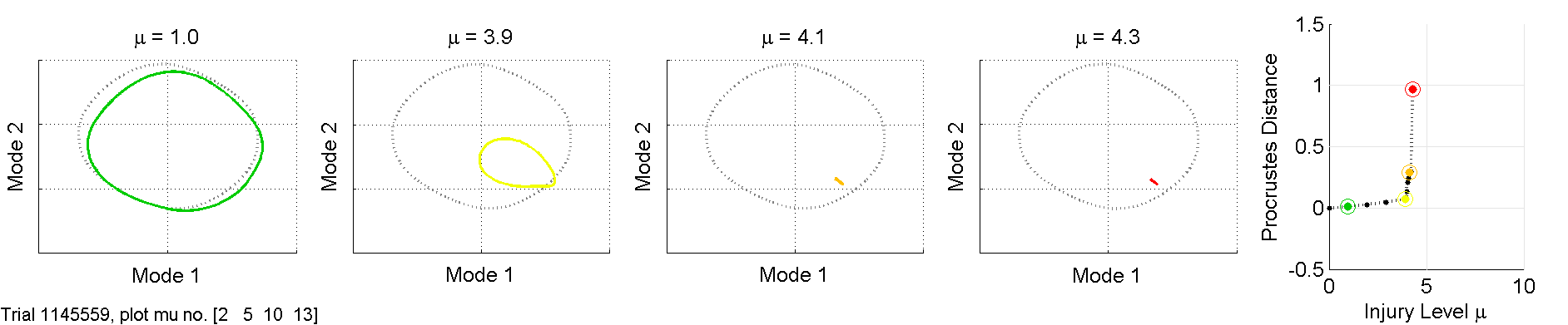

Supplement: S1 Figures — Figures similar to the rows of Fig 4, for all 1,447 trials conducted. (ZIP) [file pcbi.1005261.s002.zip › 1145559.png]

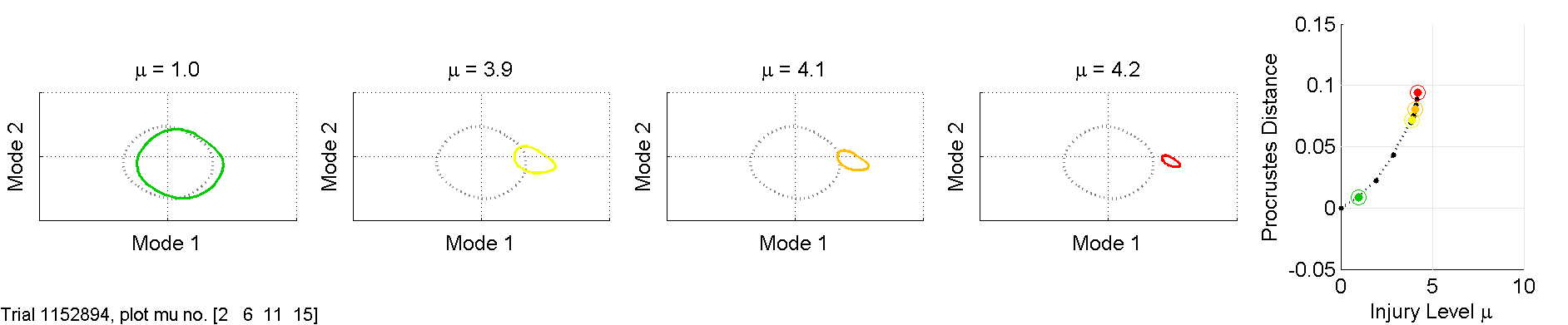

Supplement: S1 Figures — Figures similar to the rows of Fig 4, for all 1,447 trials conducted. (ZIP) [file pcbi.1005261.s002.zip › 1152894.png]

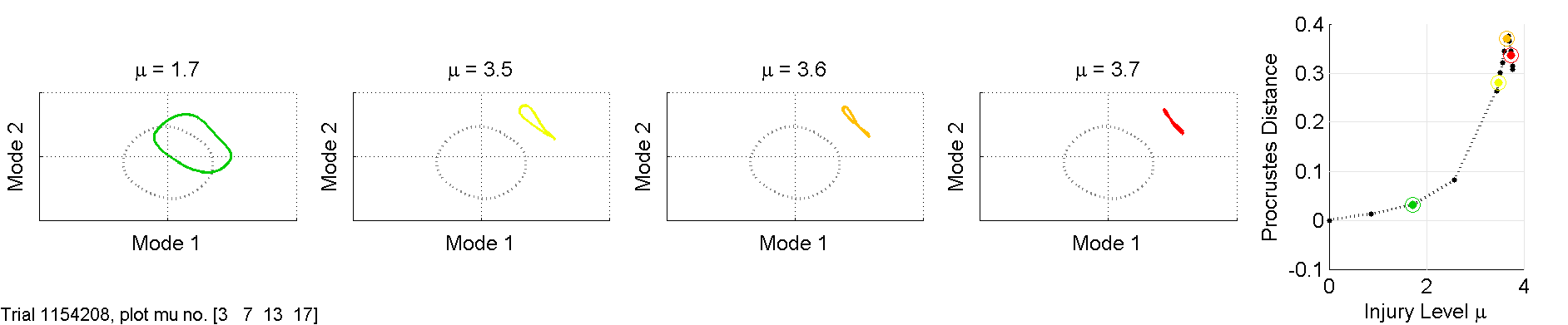

Supplement: S1 Figures — Figures similar to the rows of Fig 4, for all 1,447 trials conducted. (ZIP) [file pcbi.1005261.s002.zip › 1154208.png]

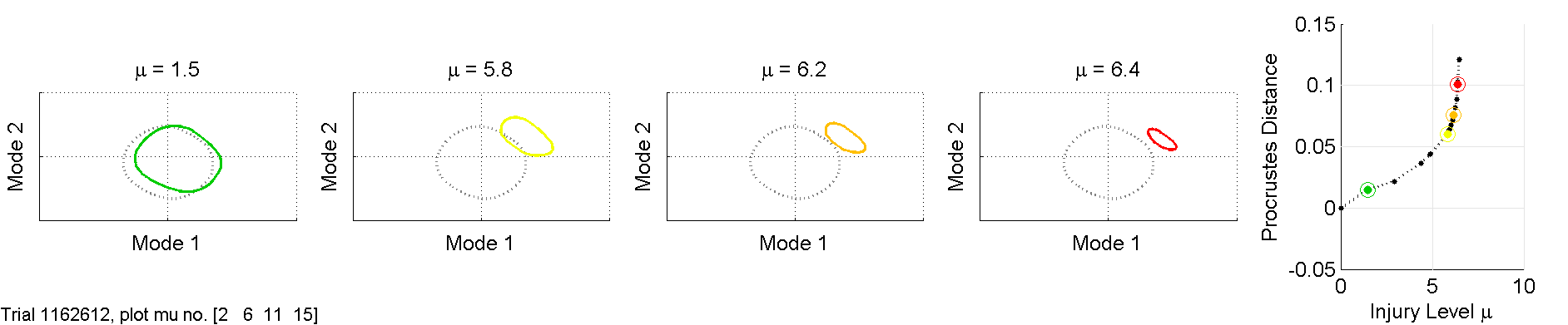

Supplement: S1 Figures — Figures similar to the rows of Fig 4, for all 1,447 trials conducted. (ZIP) [file pcbi.1005261.s002.zip › 1162612.png]

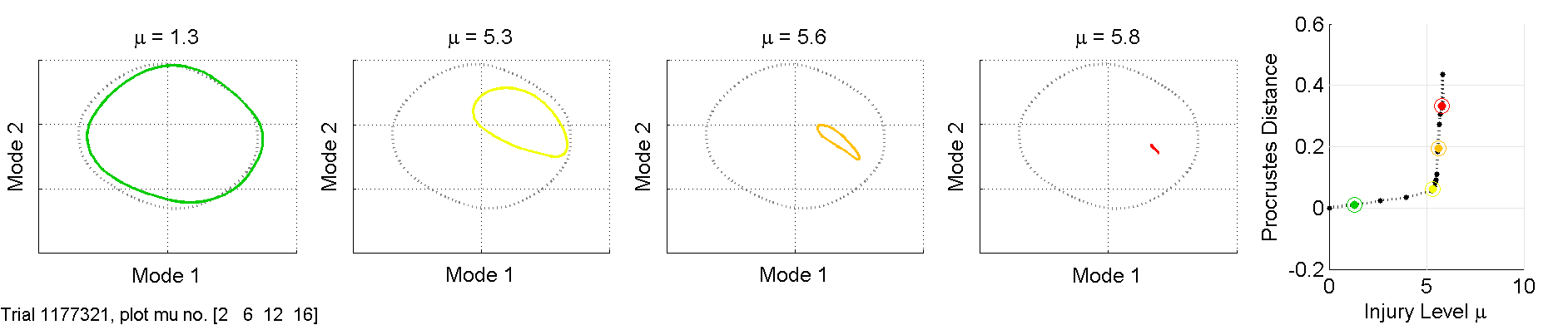

Supplement: S1 Figures — Figures similar to the rows of Fig 4, for all 1,447 trials conducted. (ZIP) [file pcbi.1005261.s002.zip › 1177321.png]

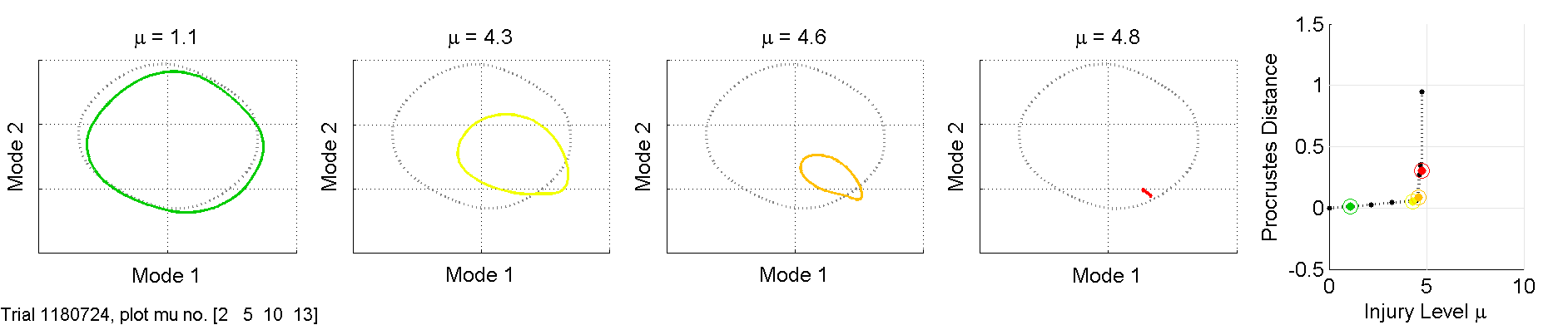

Supplement: S1 Figures — Figures similar to the rows of Fig 4, for all 1,447 trials conducted. (ZIP) [file pcbi.1005261.s002.zip › 1180724.png]

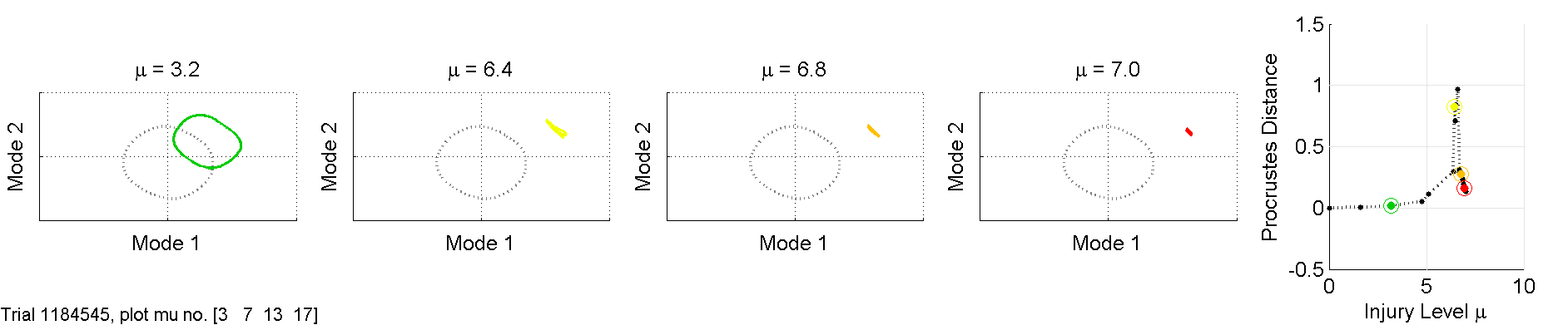

Supplement: S1 Figures — Figures similar to the rows of Fig 4, for all 1,447 trials conducted. (ZIP) [file pcbi.1005261.s002.zip › 1184545.png]

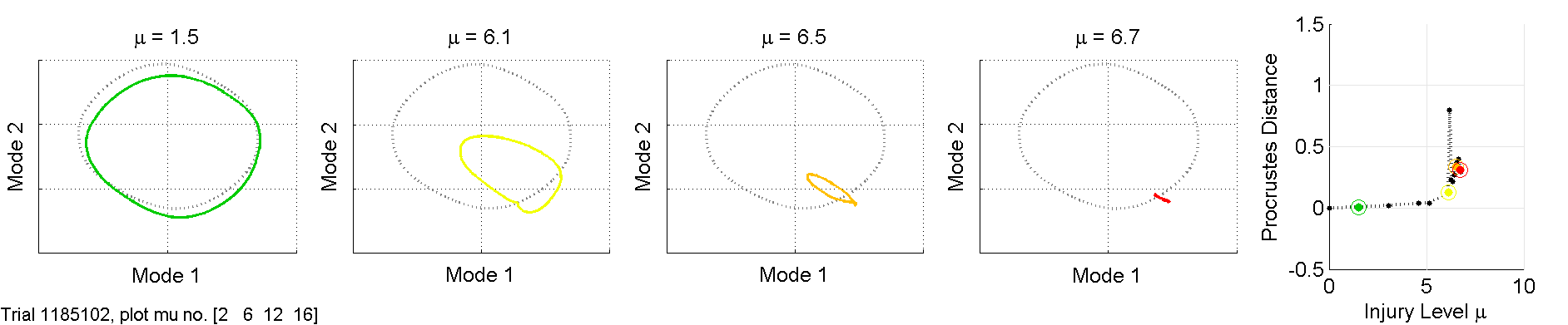

Supplement: S1 Figures — Figures similar to the rows of Fig 4, for all 1,447 trials conducted. (ZIP) [file pcbi.1005261.s002.zip › 1185102.png]

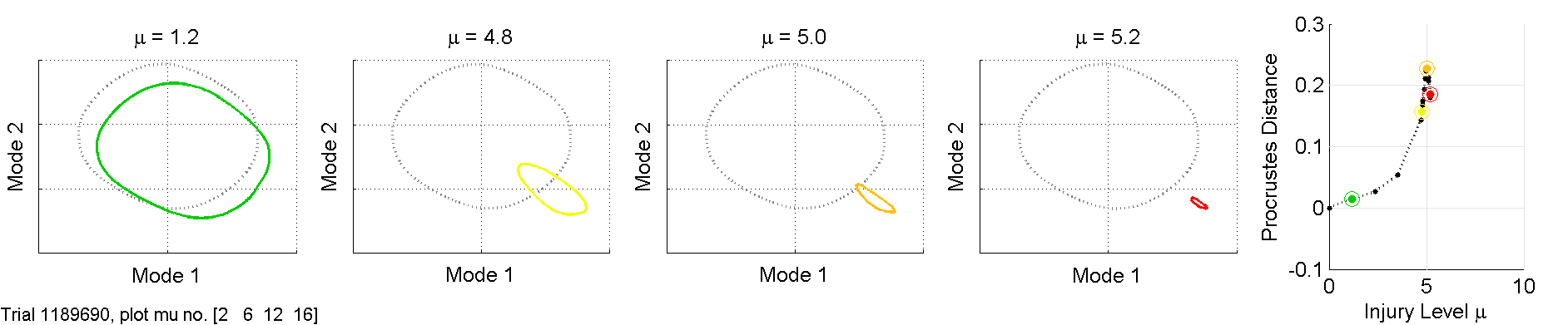

Supplement: S1 Figures — Figures similar to the rows of Fig 4, for all 1,447 trials conducted. (ZIP) [file pcbi.1005261.s002.zip › 1189690.png]

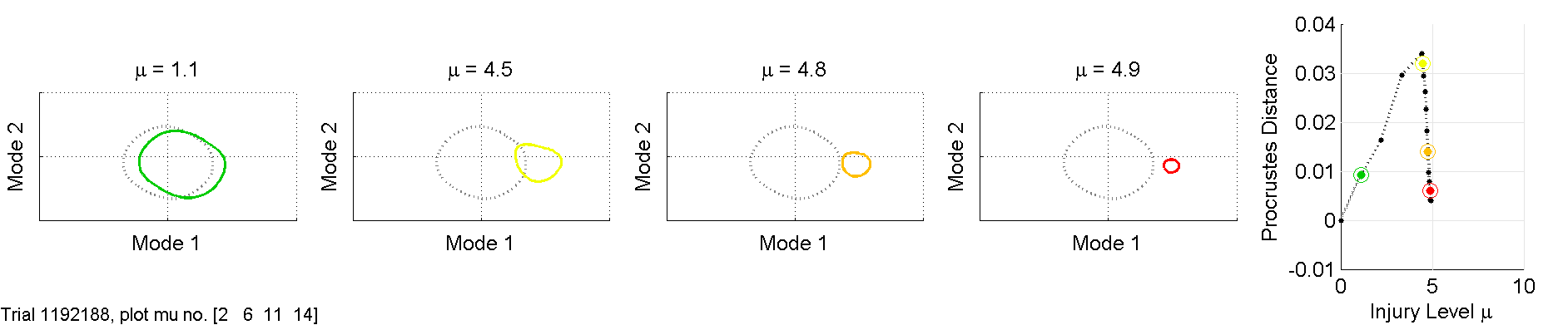

Supplement: S1 Figures — Figures similar to the rows of Fig 4, for all 1,447 trials conducted. (ZIP) [file pcbi.1005261.s002.zip › 1192188.png]

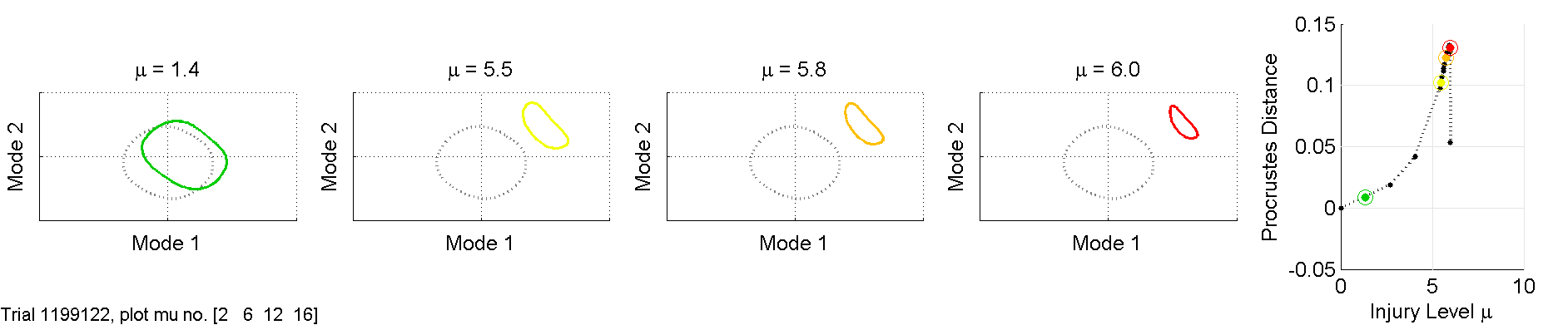

Supplement: S1 Figures — Figures similar to the rows of Fig 4, for all 1,447 trials conducted. (ZIP) [file pcbi.1005261.s002.zip › 1199122.png]

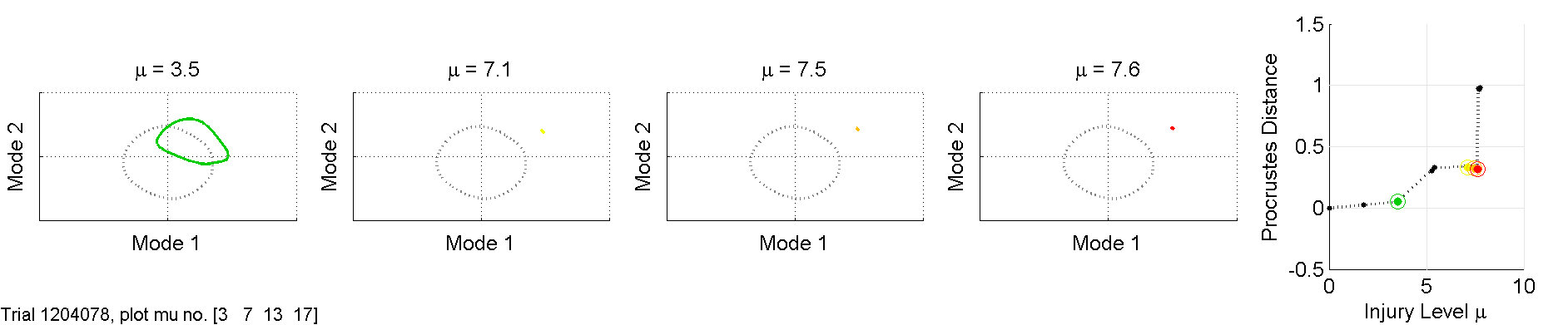

Supplement: S1 Figures — Figures similar to the rows of Fig 4, for all 1,447 trials conducted. (ZIP) [file pcbi.1005261.s002.zip › 1204078.png]

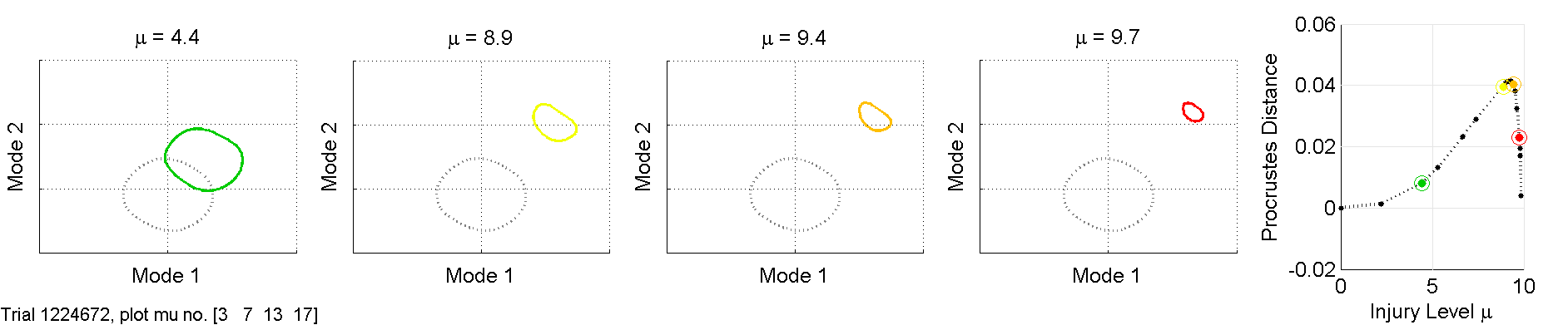

Supplement: S1 Figures — Figures similar to the rows of Fig 4, for all 1,447 trials conducted. (ZIP) [file pcbi.1005261.s002.zip › 1224672.png]

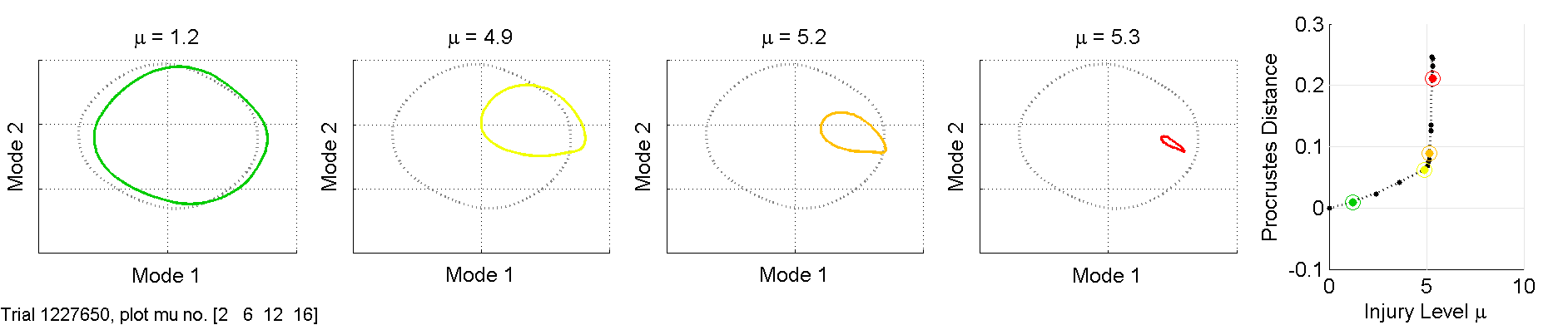

Supplement: S1 Figures — Figures similar to the rows of Fig 4, for all 1,447 trials conducted. (ZIP) [file pcbi.1005261.s002.zip › 1227650.png]

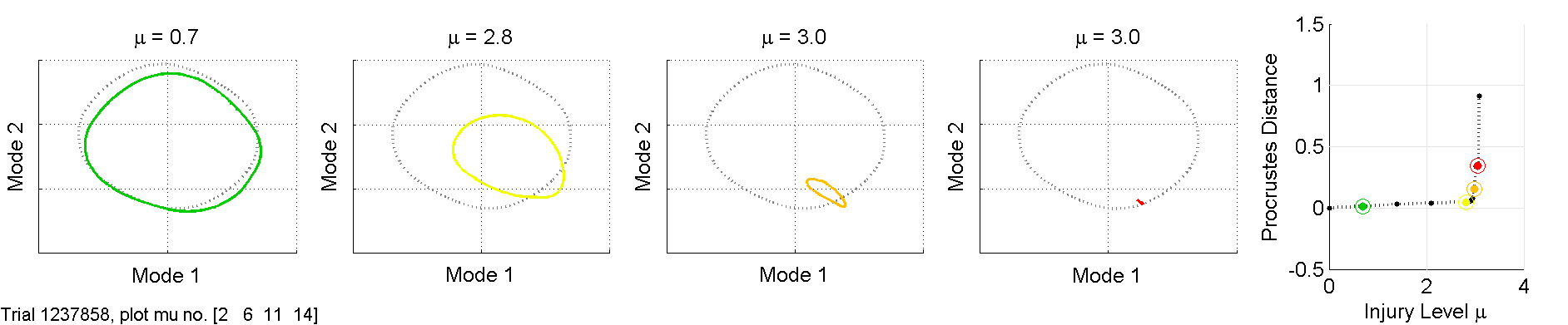

Supplement: S1 Figures — Figures similar to the rows of Fig 4, for all 1,447 trials conducted. (ZIP) [file pcbi.1005261.s002.zip › 1237858.png]

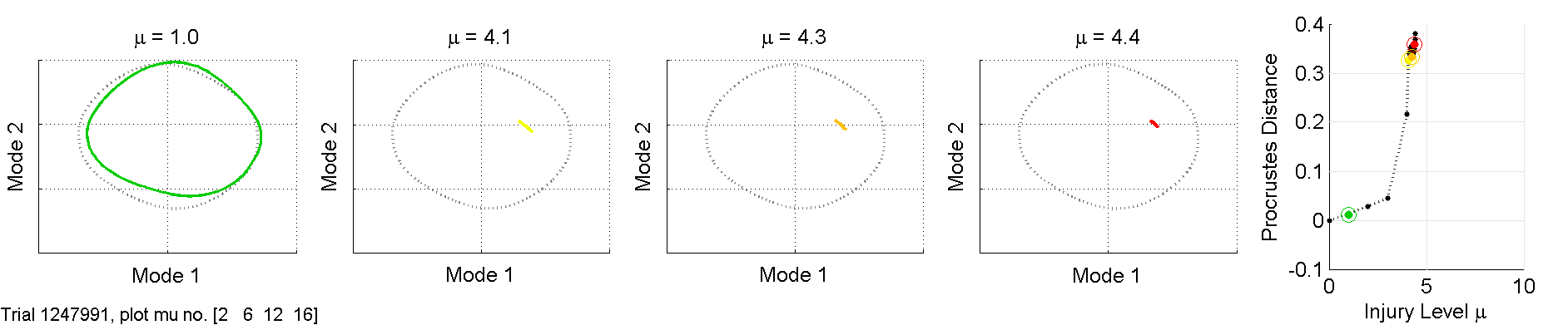

Supplement: S1 Figures — Figures similar to the rows of Fig 4, for all 1,447 trials conducted. (ZIP) [file pcbi.1005261.s002.zip › 1247991.png]

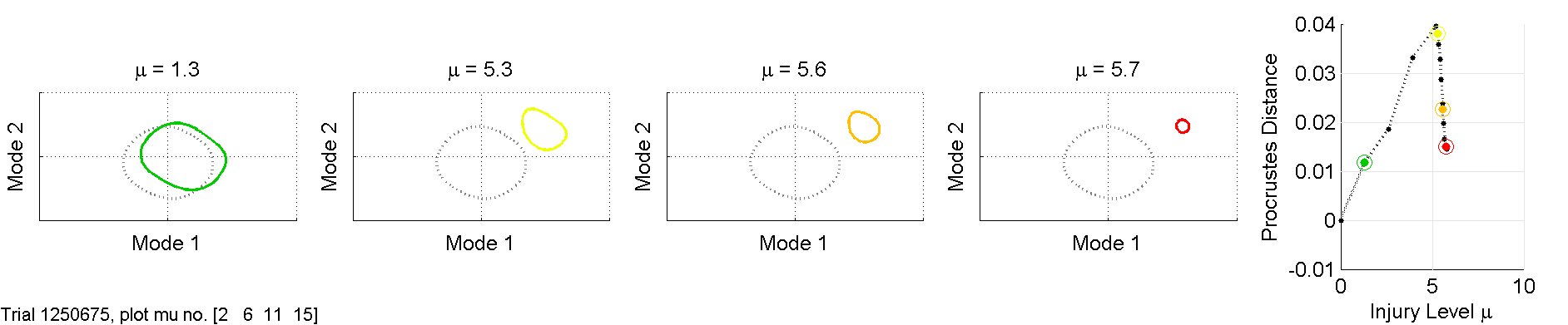

Supplement: S1 Figures — Figures similar to the rows of Fig 4, for all 1,447 trials conducted. (ZIP) [file pcbi.1005261.s002.zip › 1250675.png]

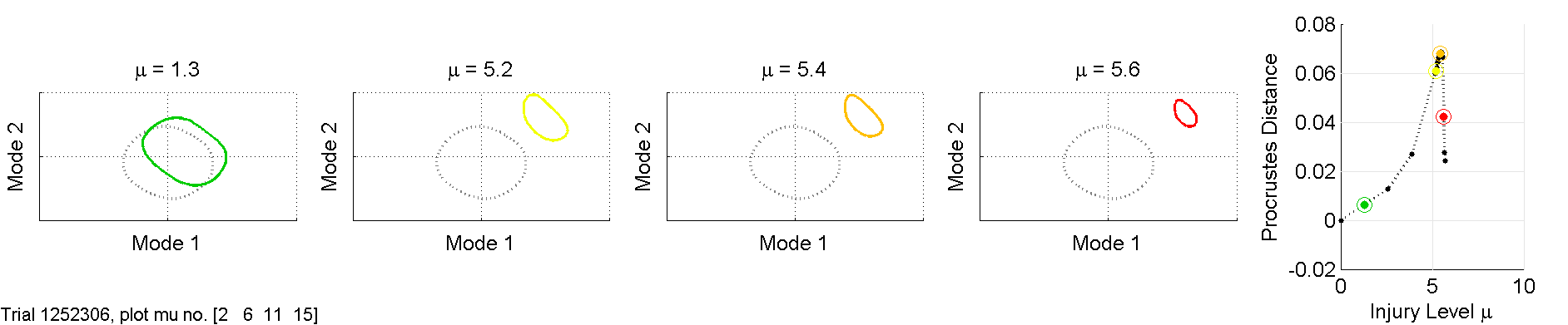

Supplement: S1 Figures — Figures similar to the rows of Fig 4, for all 1,447 trials conducted. (ZIP) [file pcbi.1005261.s002.zip › 1252306.png]

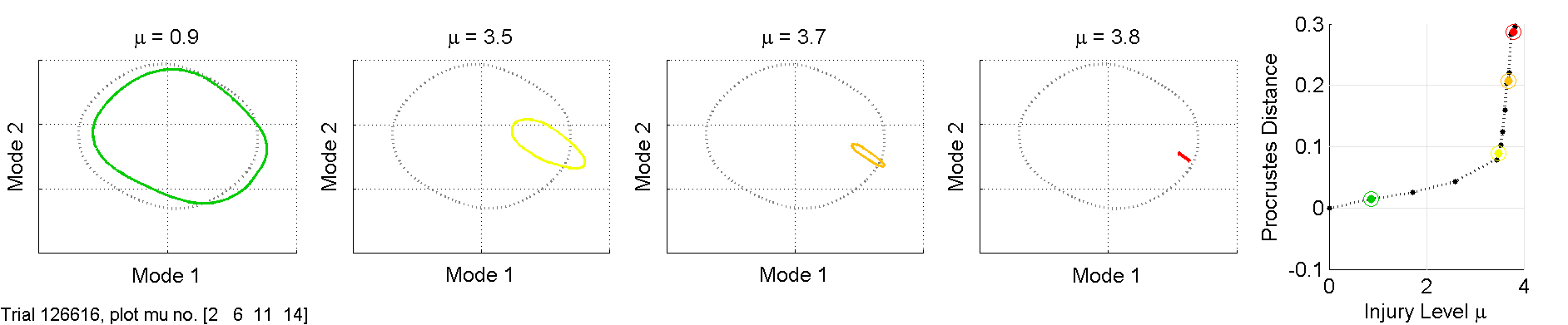

Supplement: S1 Figures — Figures similar to the rows of Fig 4, for all 1,447 trials conducted. (ZIP) [file pcbi.1005261.s002.zip › 126616.png]

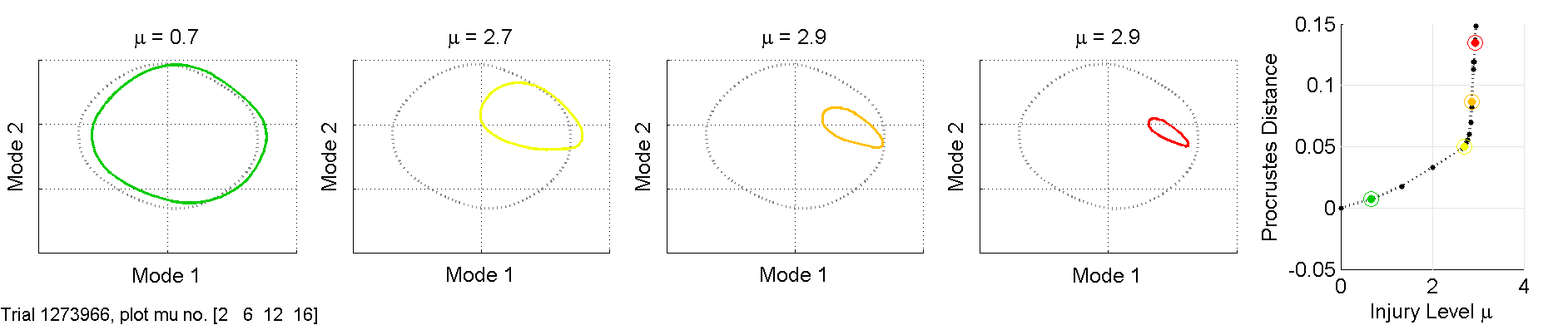

Supplement: S1 Figures — Figures similar to the rows of Fig 4, for all 1,447 trials conducted. (ZIP) [file pcbi.1005261.s002.zip › 1273966.png]

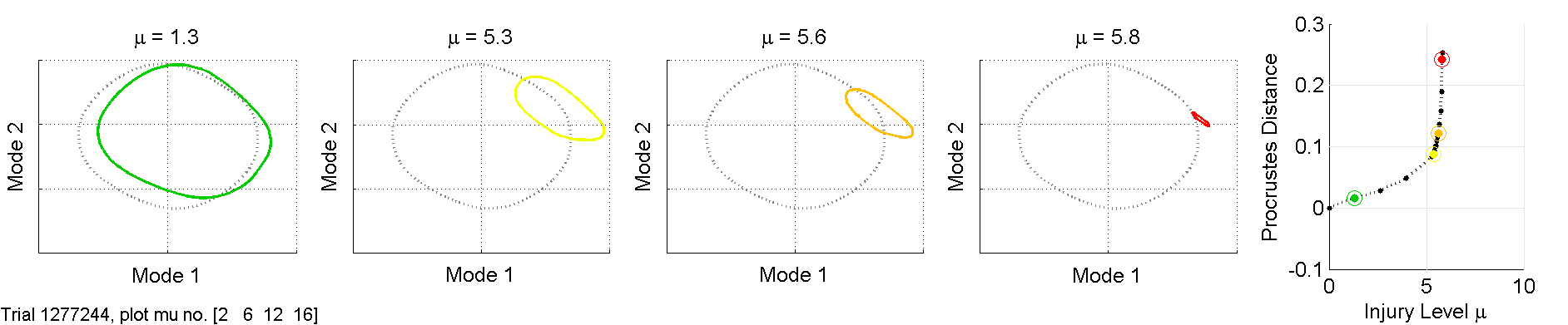

Supplement: S1 Figures — Figures similar to the rows of Fig 4, for all 1,447 trials conducted. (ZIP) [file pcbi.1005261.s002.zip › 1277244.png]

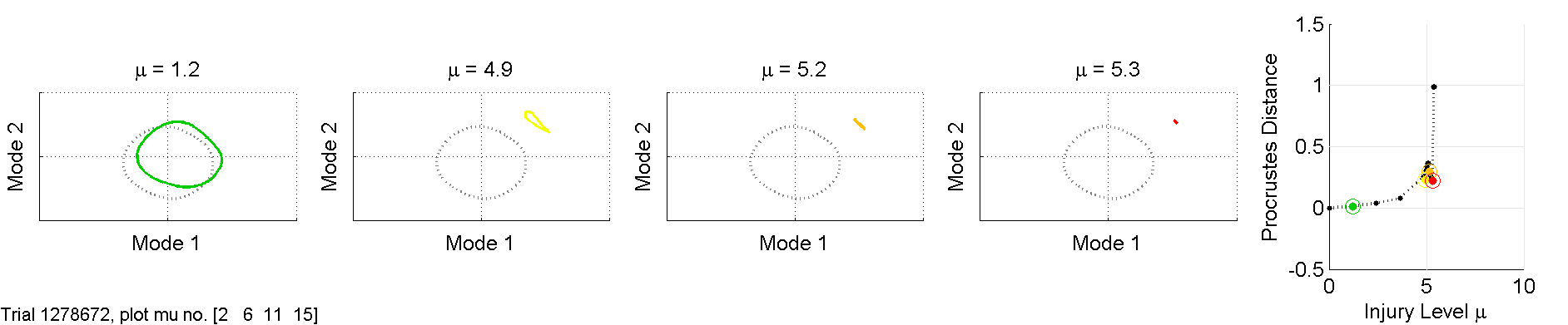

Supplement: S1 Figures — Figures similar to the rows of Fig 4, for all 1,447 trials conducted. (ZIP) [file pcbi.1005261.s002.zip › 1278672.png]

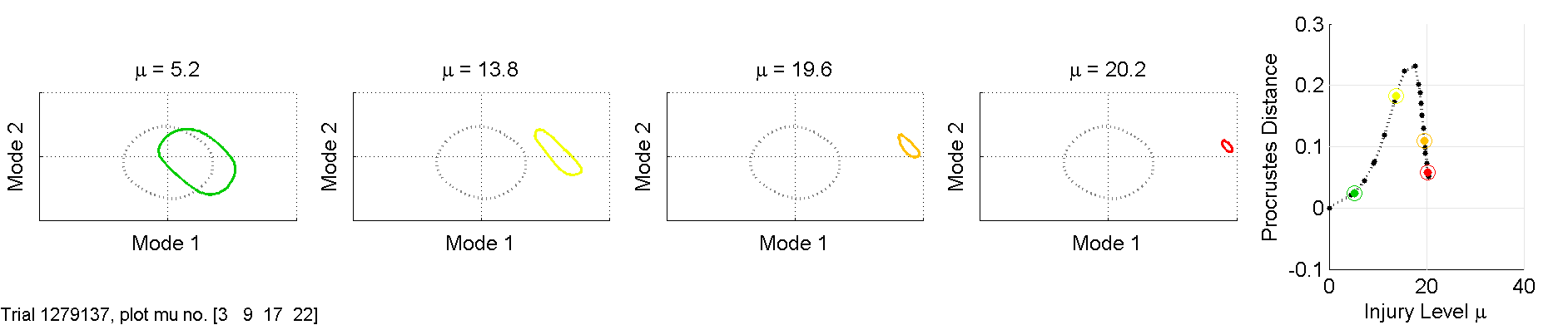

Supplement: S1 Figures — Figures similar to the rows of Fig 4, for all 1,447 trials conducted. (ZIP) [file pcbi.1005261.s002.zip › 1279137.png]

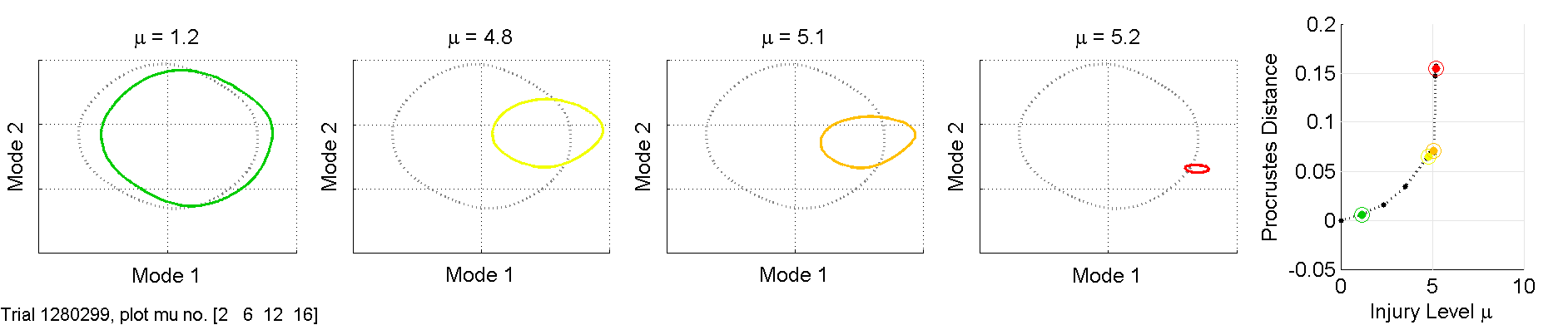

Supplement: S1 Figures — Figures similar to the rows of Fig 4, for all 1,447 trials conducted. (ZIP) [file pcbi.1005261.s002.zip › 1280299.png]

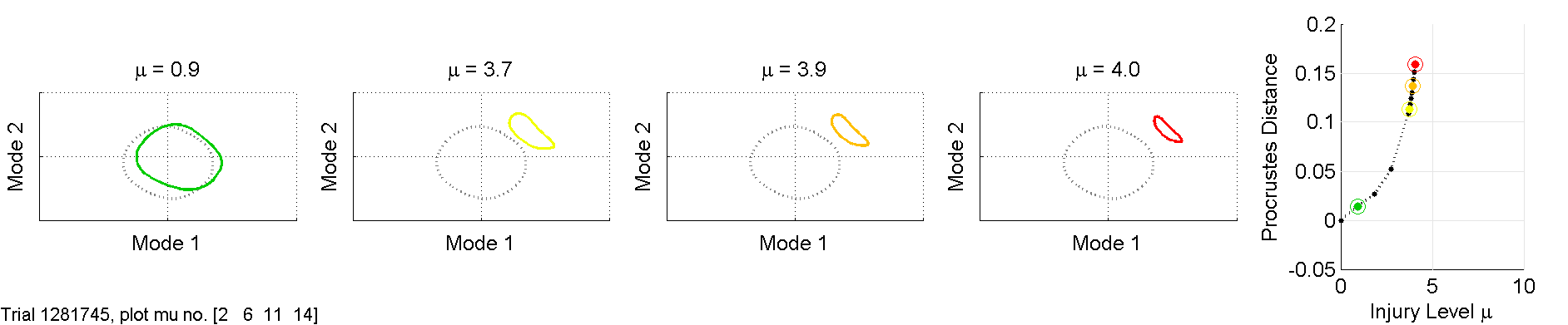

Supplement: S1 Figures — Figures similar to the rows of Fig 4, for all 1,447 trials conducted. (ZIP) [file pcbi.1005261.s002.zip › 1281745.png]

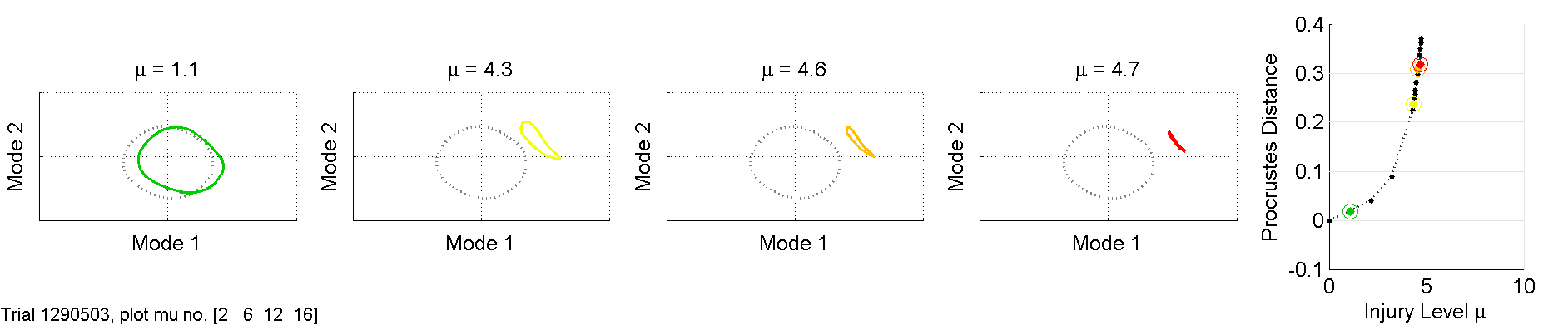

Supplement: S1 Figures — Figures similar to the rows of Fig 4, for all 1,447 trials conducted. (ZIP) [file pcbi.1005261.s002.zip › 1290503.png]

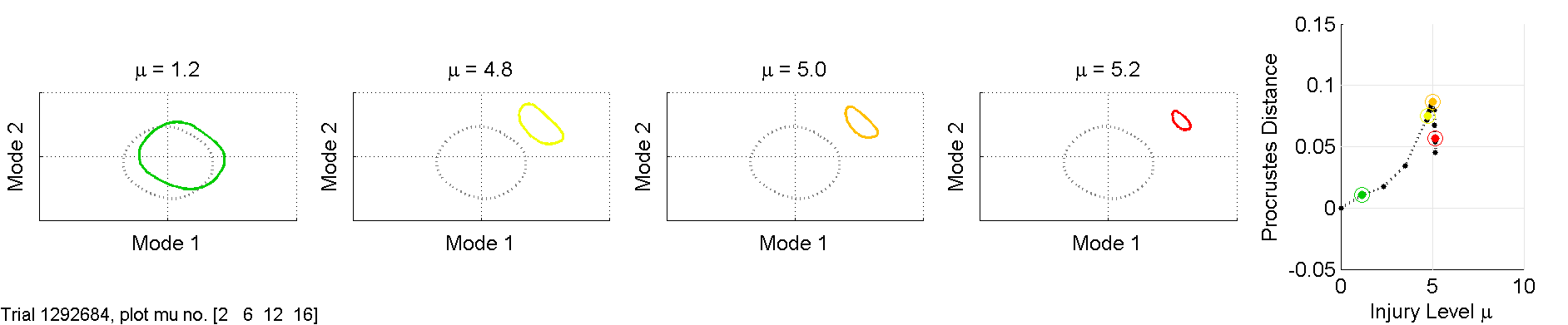

Supplement: S1 Figures — Figures similar to the rows of Fig 4, for all 1,447 trials conducted. (ZIP) [file pcbi.1005261.s002.zip › 1292684.png]

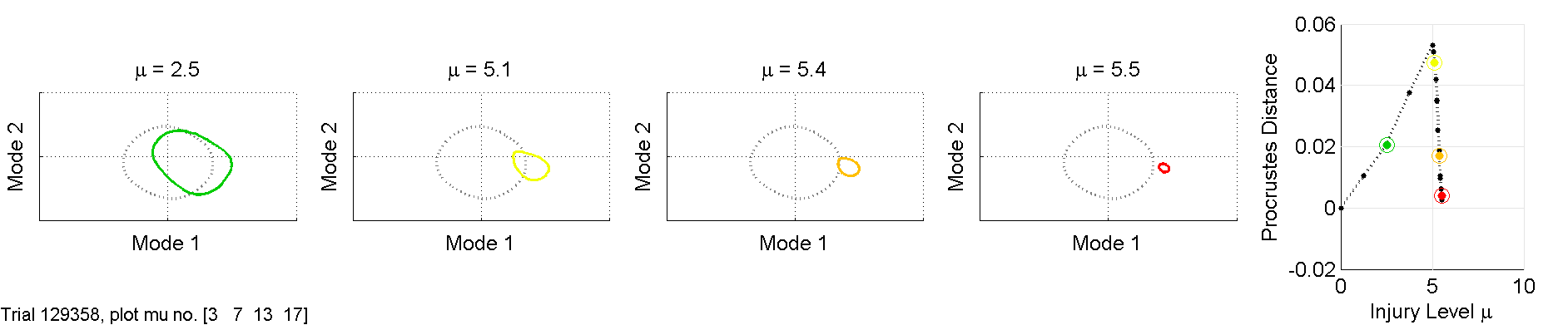

Supplement: S1 Figures — Figures similar to the rows of Fig 4, for all 1,447 trials conducted. (ZIP) [file pcbi.1005261.s002.zip › 129358.png]

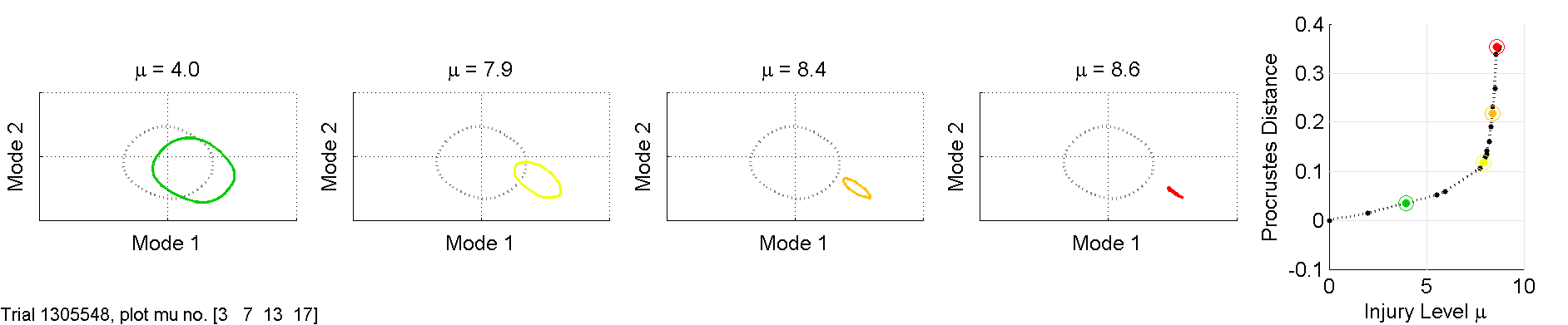

Supplement: S1 Figures — Figures similar to the rows of Fig 4, for all 1,447 trials conducted. (ZIP) [file pcbi.1005261.s002.zip › 1305548.png]

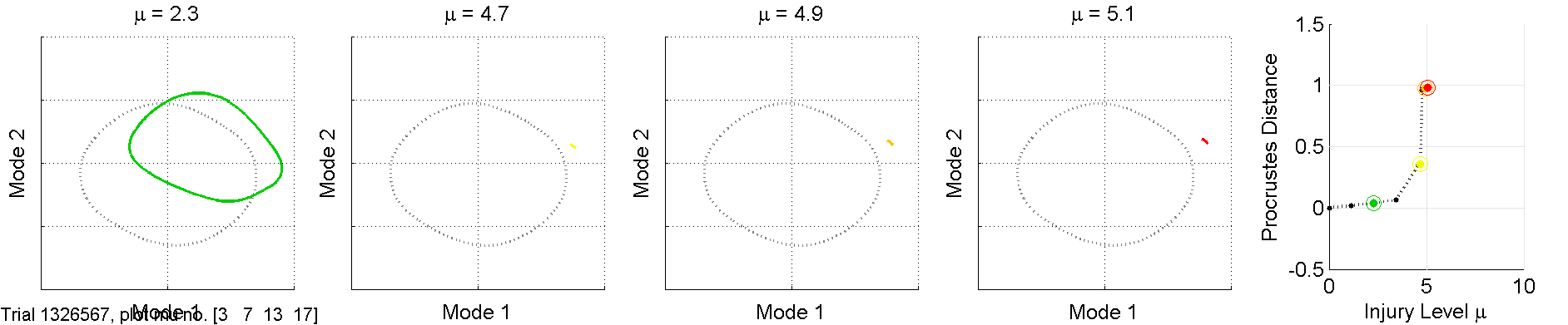

Supplement: S1 Figures — Figures similar to the rows of Fig 4, for all 1,447 trials conducted. (ZIP) [file pcbi.1005261.s002.zip › 1326567.png]

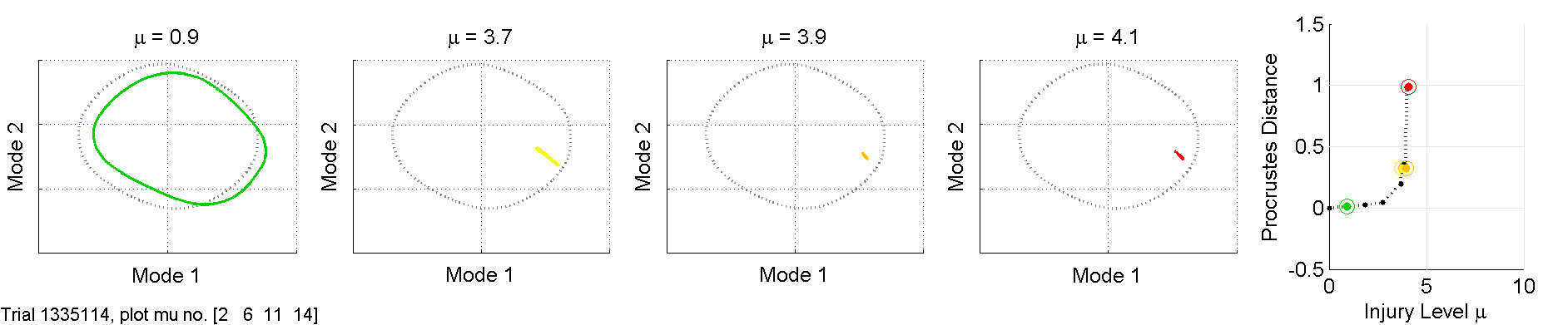

Supplement: S1 Figures — Figures similar to the rows of Fig 4, for all 1,447 trials conducted. (ZIP) [file pcbi.1005261.s002.zip › 1335114.png]

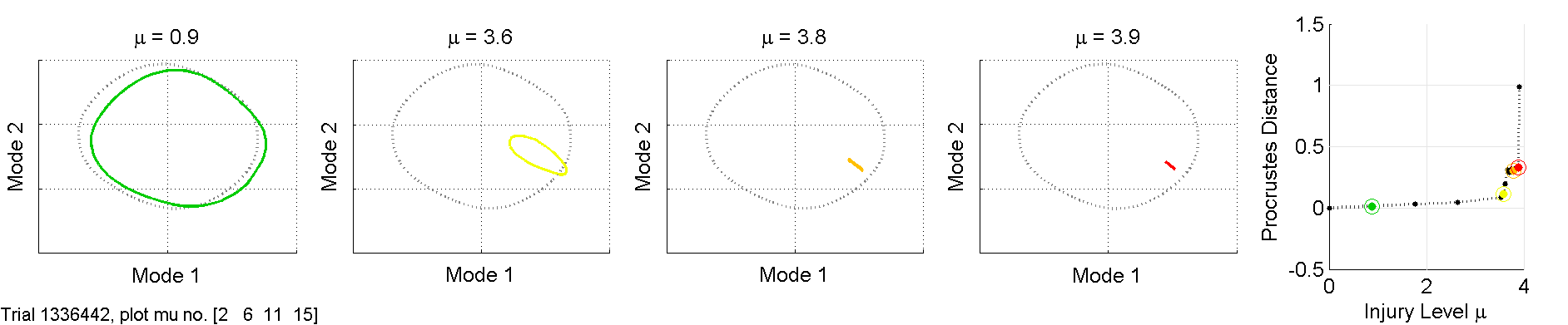

Supplement: S1 Figures — Figures similar to the rows of Fig 4, for all 1,447 trials conducted. (ZIP) [file pcbi.1005261.s002.zip › 1336442.png]

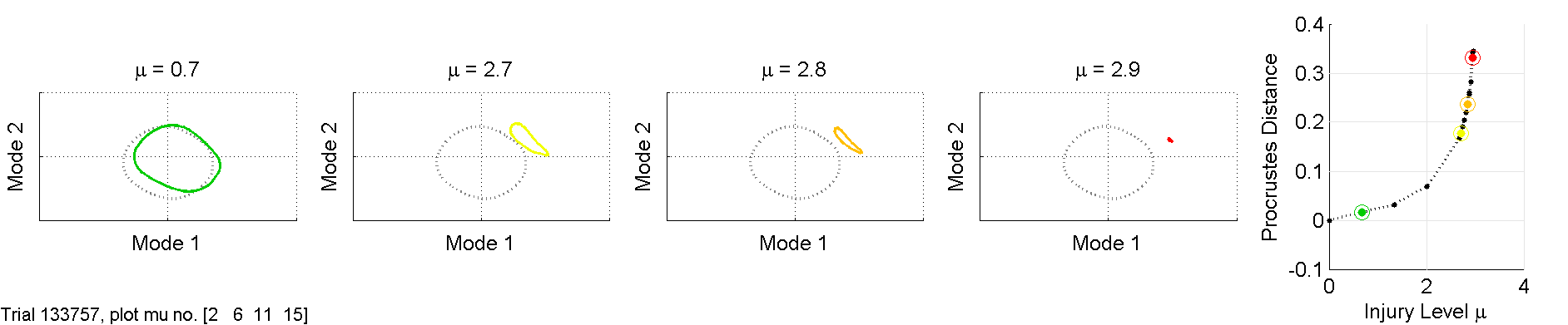

Supplement: S1 Figures — Figures similar to the rows of Fig 4, for all 1,447 trials conducted. (ZIP) [file pcbi.1005261.s002.zip › 133757.png]

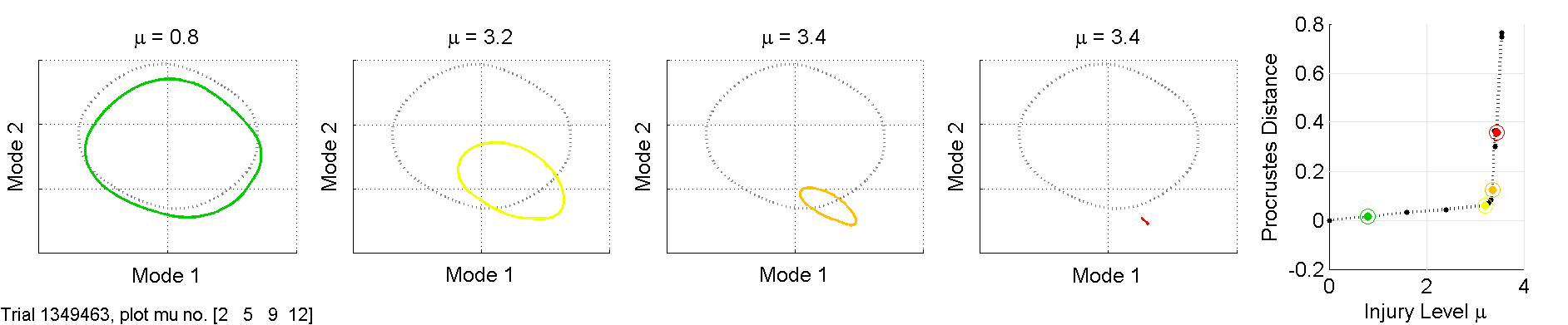

Supplement: S1 Figures — Figures similar to the rows of Fig 4, for all 1,447 trials conducted. (ZIP) [file pcbi.1005261.s002.zip › 1349463.png]

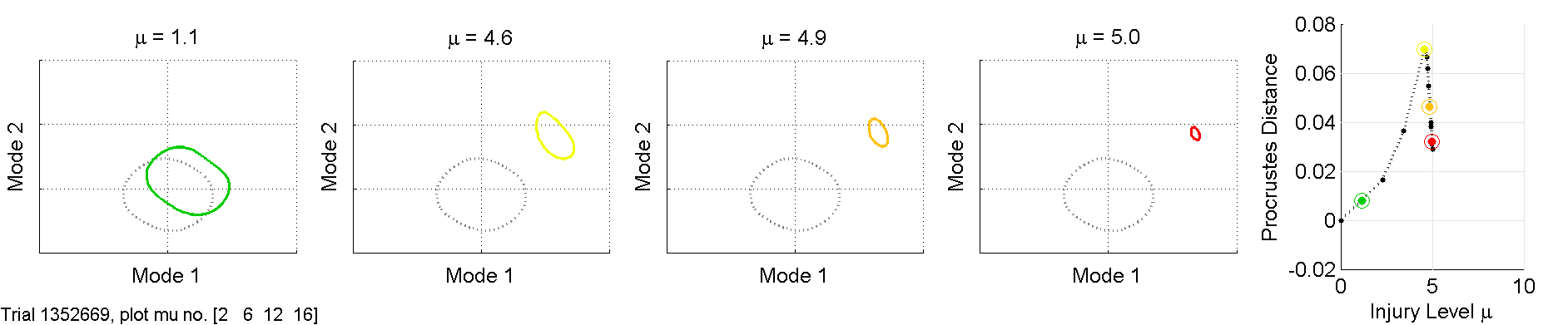

Supplement: S1 Figures — Figures similar to the rows of Fig 4, for all 1,447 trials conducted. (ZIP) [file pcbi.1005261.s002.zip › 1352669.png]

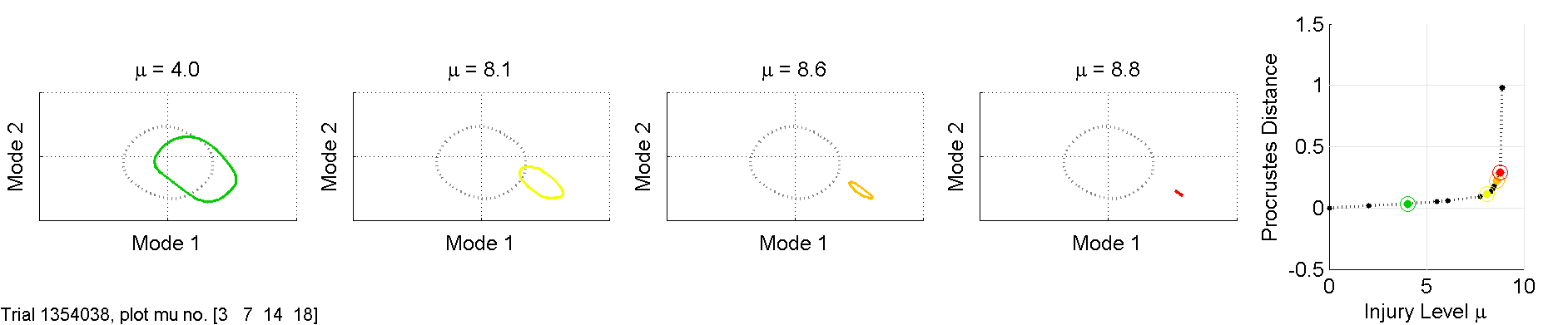

Supplement: S1 Figures — Figures similar to the rows of Fig 4, for all 1,447 trials conducted. (ZIP) [file pcbi.1005261.s002.zip › 1354038.png]

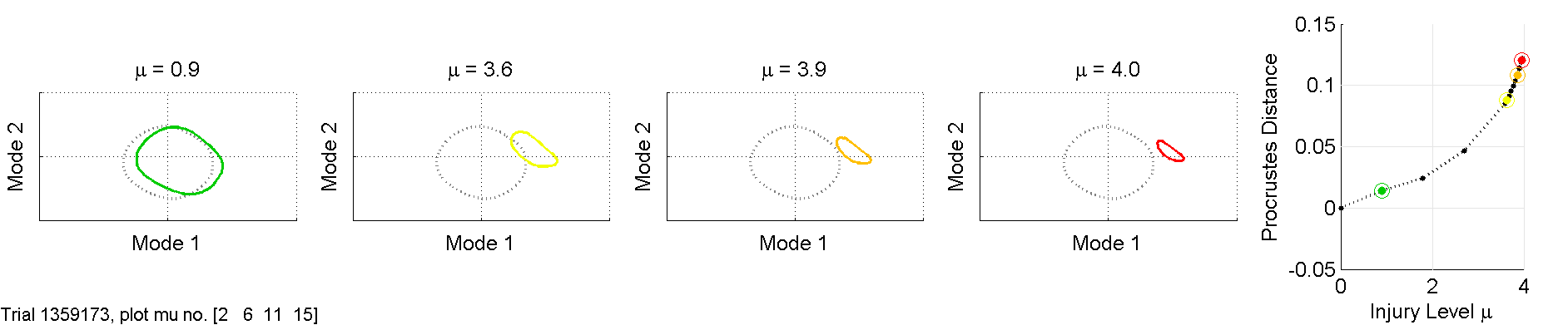

Supplement: S1 Figures — Figures similar to the rows of Fig 4, for all 1,447 trials conducted. (ZIP) [file pcbi.1005261.s002.zip › 1359173.png]

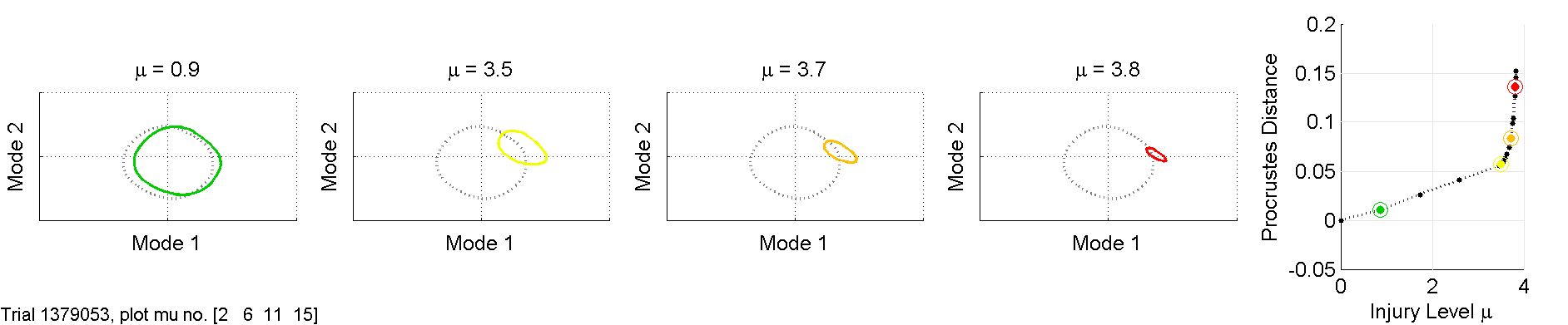

Supplement: S1 Figures — Figures similar to the rows of Fig 4, for all 1,447 trials conducted. (ZIP) [file pcbi.1005261.s002.zip › 1379053.png]

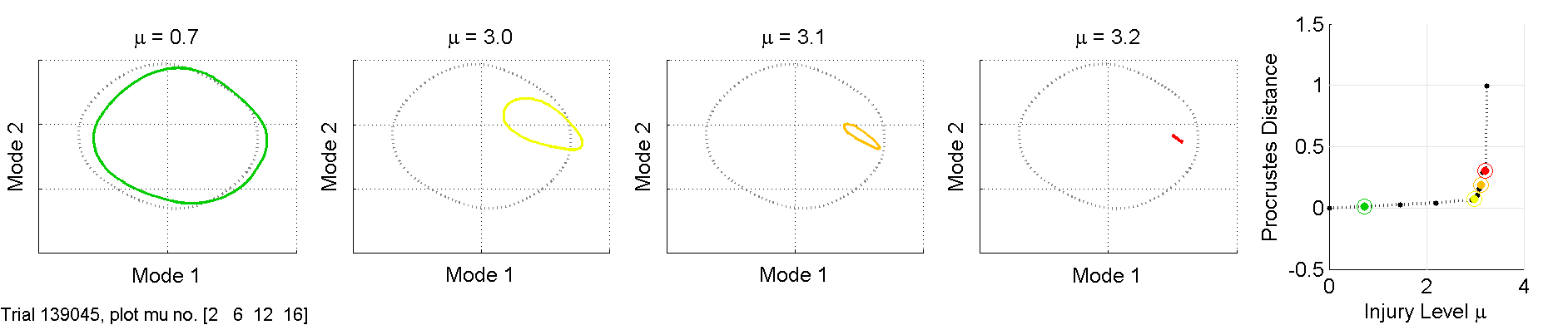

Supplement: S1 Figures — Figures similar to the rows of Fig 4, for all 1,447 trials conducted. (ZIP) [file pcbi.1005261.s002.zip › 139045.png]

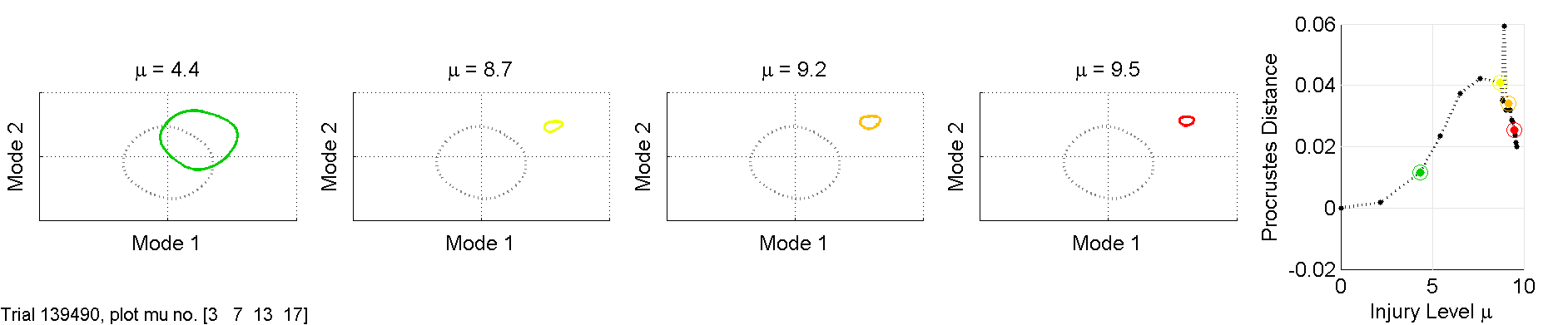

Supplement: S1 Figures — Figures similar to the rows of Fig 4, for all 1,447 trials conducted. (ZIP) [file pcbi.1005261.s002.zip › 139490.png]

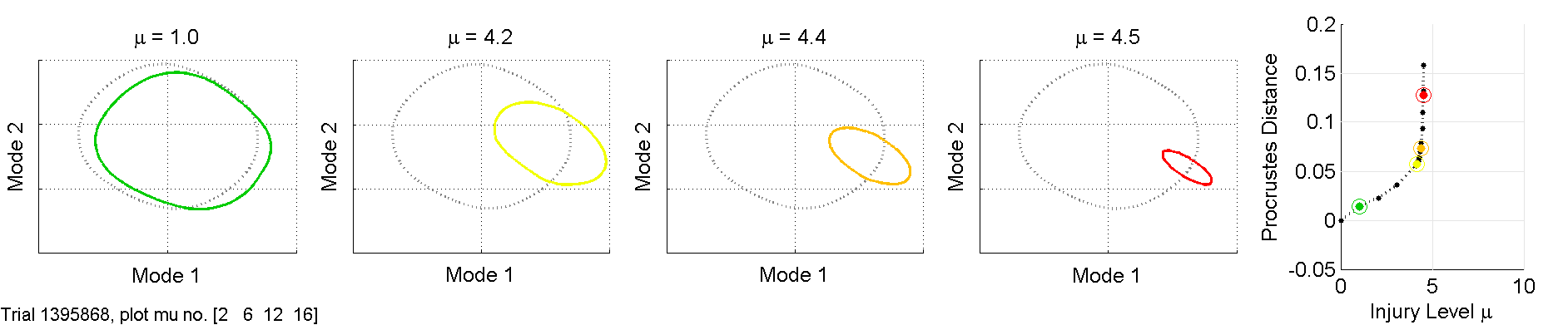

Supplement: S1 Figures — Figures similar to the rows of Fig 4, for all 1,447 trials conducted. (ZIP) [file pcbi.1005261.s002.zip › 1395868.png]

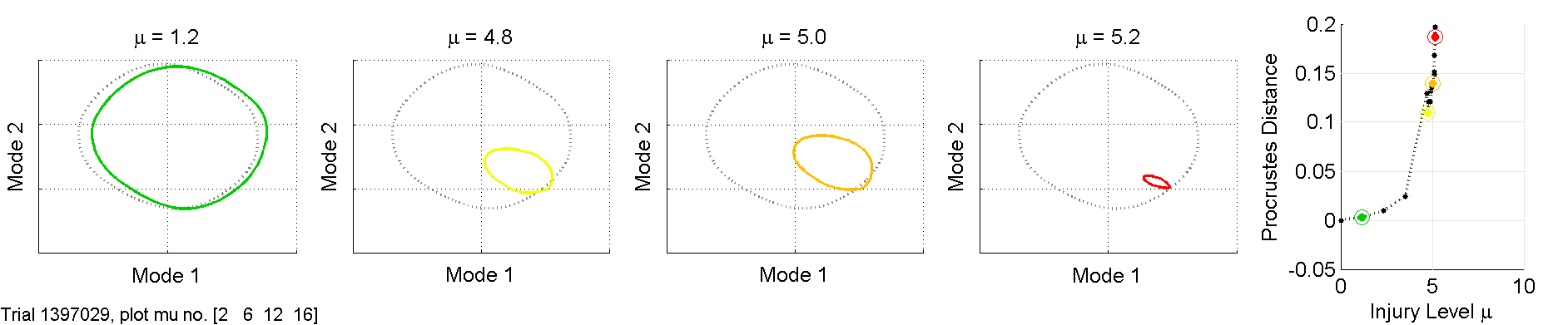

Supplement: S1 Figures — Figures similar to the rows of Fig 4, for all 1,447 trials conducted. (ZIP) [file pcbi.1005261.s002.zip › 1397029.png]

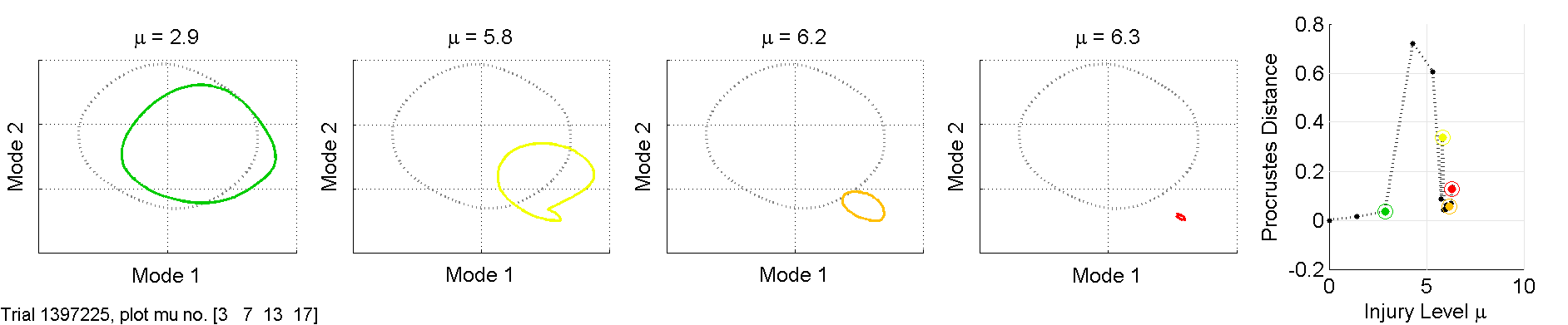

Supplement: S1 Figures — Figures similar to the rows of Fig 4, for all 1,447 trials conducted. (ZIP) [file pcbi.1005261.s002.zip › 1397225.png]

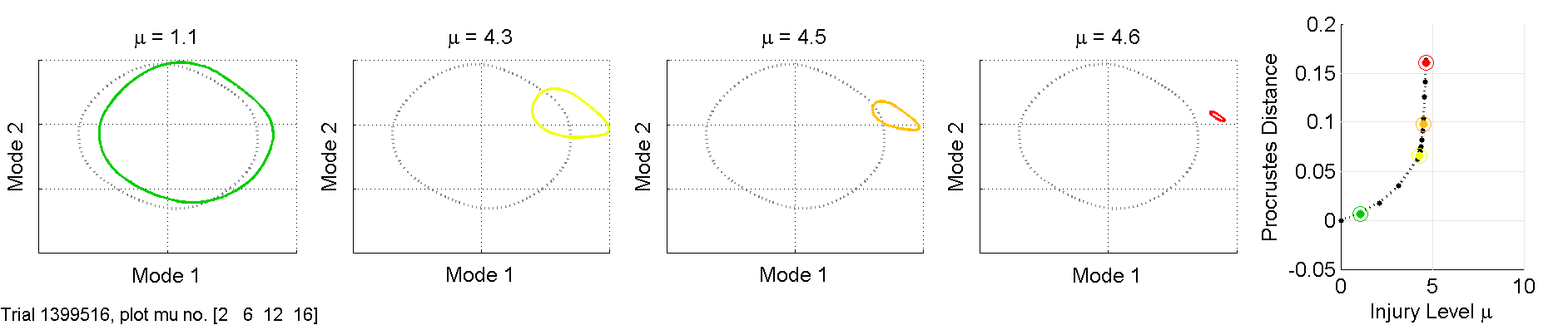

Supplement: S1 Figures — Figures similar to the rows of Fig 4, for all 1,447 trials conducted. (ZIP) [file pcbi.1005261.s002.zip › 1399516.png]

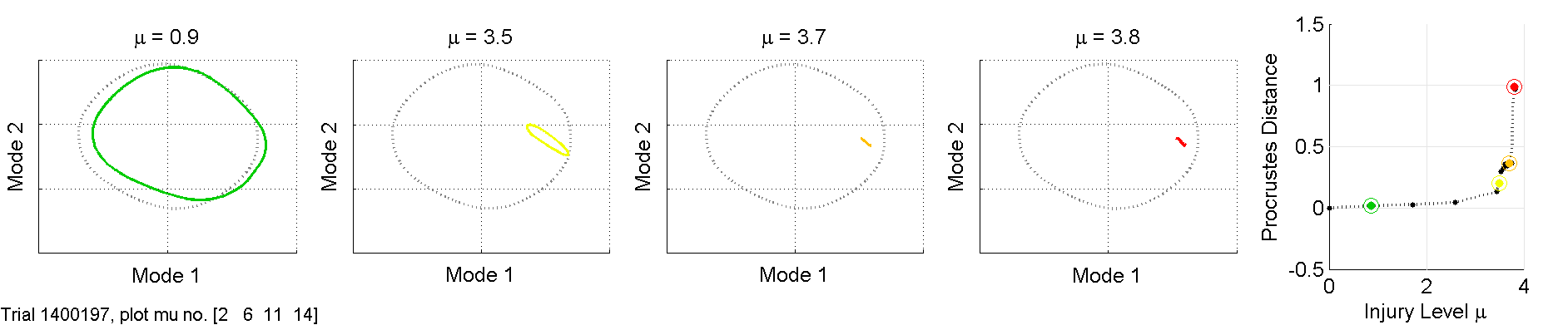

Supplement: S1 Figures — Figures similar to the rows of Fig 4, for all 1,447 trials conducted. (ZIP) [file pcbi.1005261.s002.zip › 1400197.png]

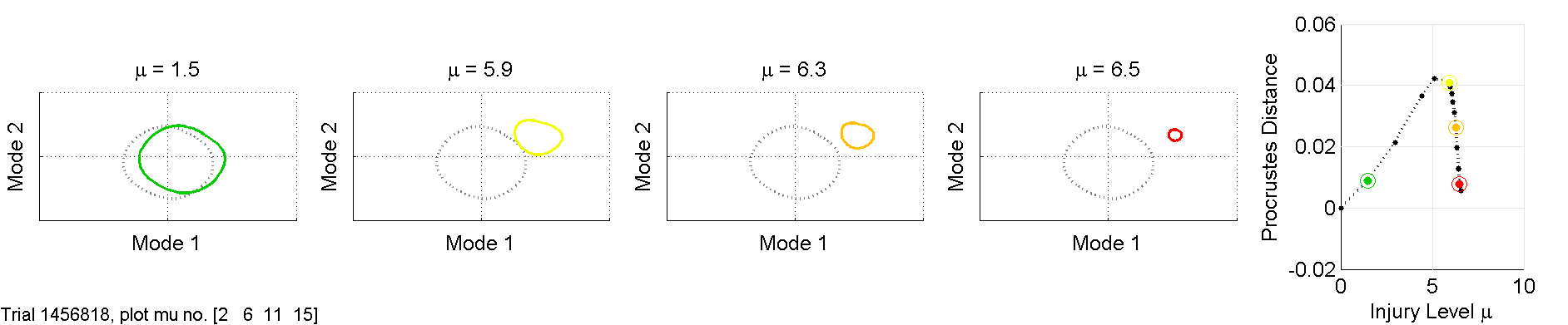

Supplement: S1 Figures — Figures similar to the rows of Fig 4, for all 1,447 trials conducted. (ZIP) [file pcbi.1005261.s002.zip › 1456818.png]

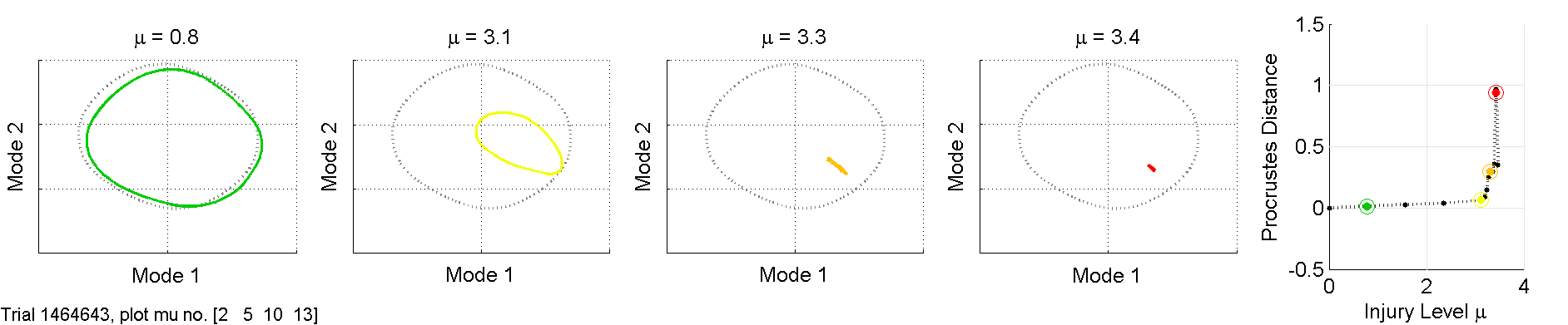

Supplement: S1 Figures — Figures similar to the rows of Fig 4, for all 1,447 trials conducted. (ZIP) [file pcbi.1005261.s002.zip › 1464643.png]

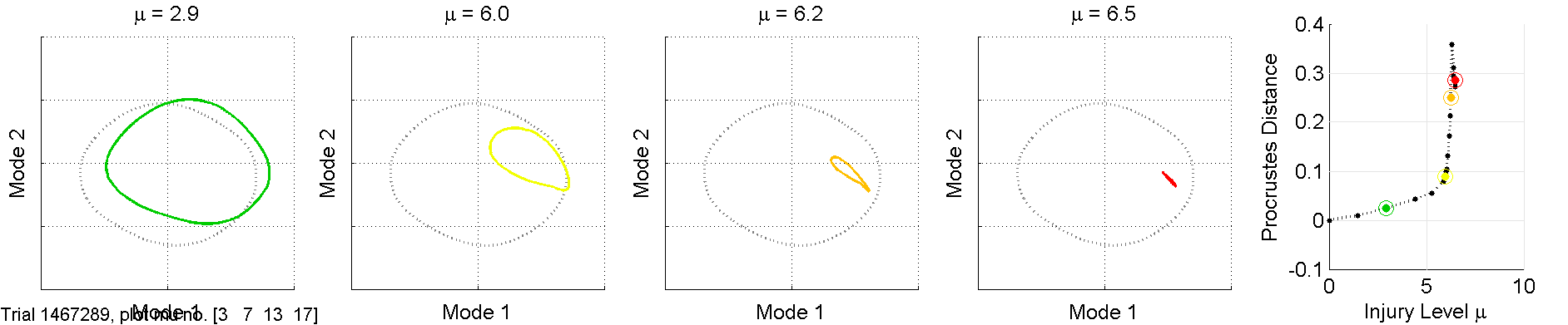

Supplement: S1 Figures — Figures similar to the rows of Fig 4, for all 1,447 trials conducted. (ZIP) [file pcbi.1005261.s002.zip › 1467289.png]

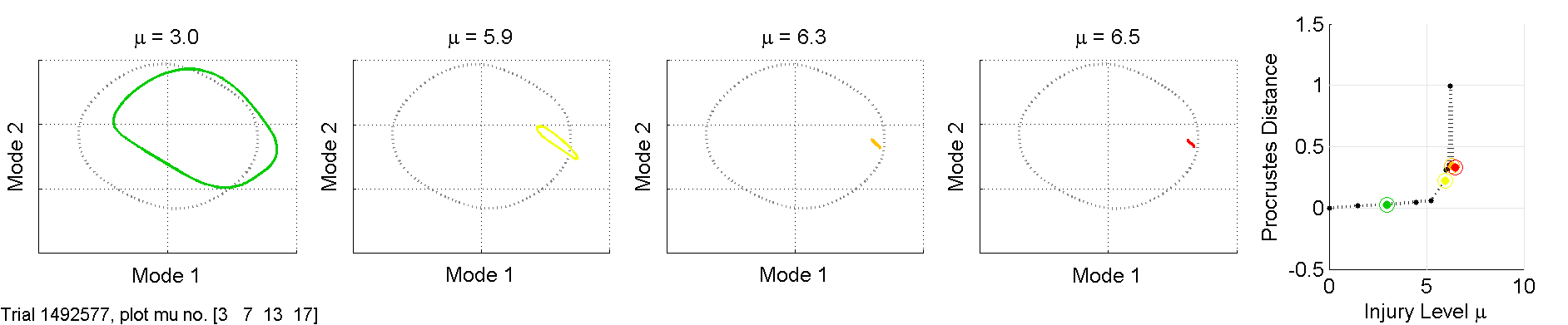

Supplement: S1 Figures — Figures similar to the rows of Fig 4, for all 1,447 trials conducted. (ZIP) [file pcbi.1005261.s002.zip › 1492577.png]

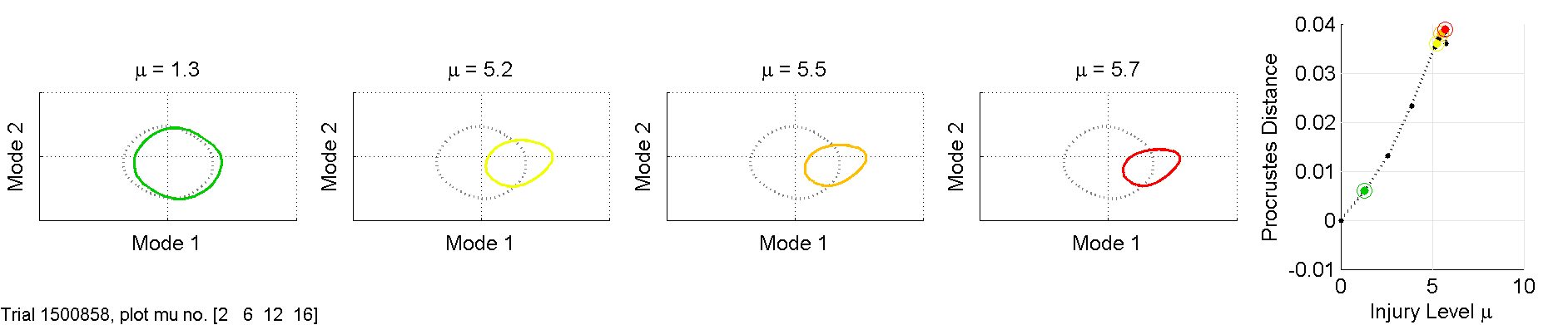

Supplement: S1 Figures — Figures similar to the rows of Fig 4, for all 1,447 trials conducted. (ZIP) [file pcbi.1005261.s002.zip › 1500858.png]

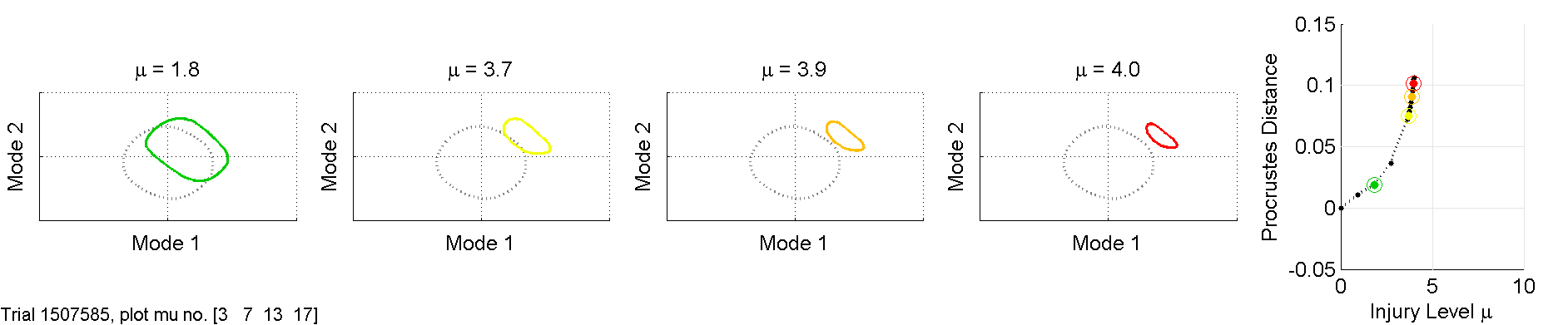

Supplement: S1 Figures — Figures similar to the rows of Fig 4, for all 1,447 trials conducted. (ZIP) [file pcbi.1005261.s002.zip › 1507585.png]

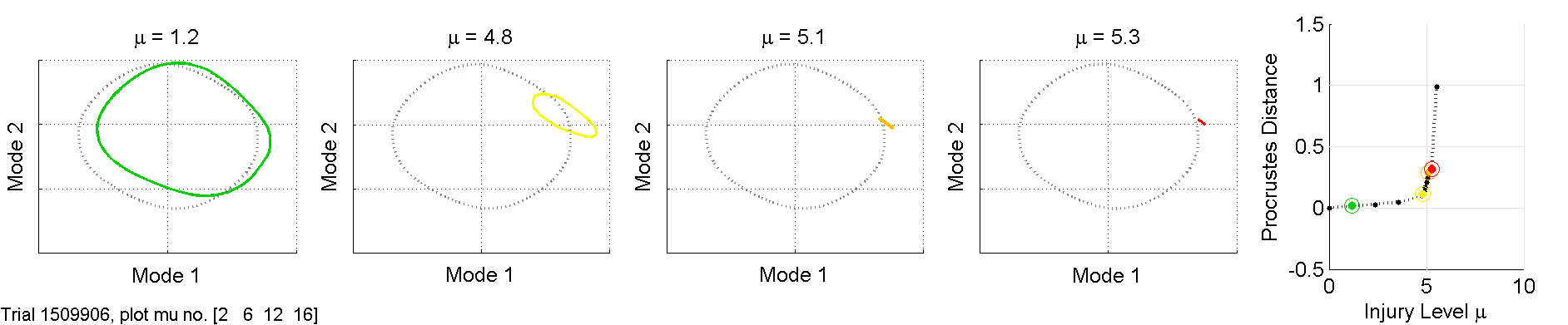

Supplement: S1 Figures — Figures similar to the rows of Fig 4, for all 1,447 trials conducted. (ZIP) [file pcbi.1005261.s002.zip › 1509906.png]

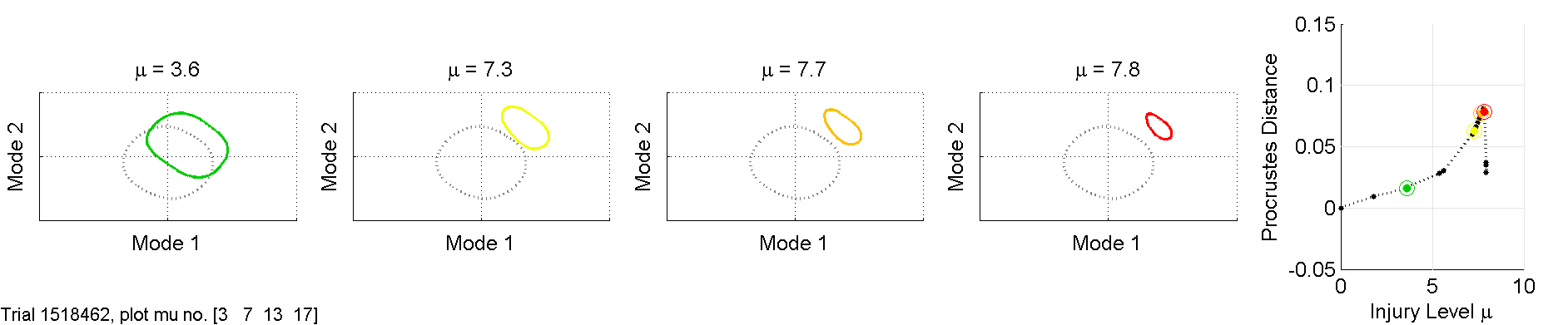

Supplement: S1 Figures — Figures similar to the rows of Fig 4, for all 1,447 trials conducted. (ZIP) [file pcbi.1005261.s002.zip › 1518462.png]

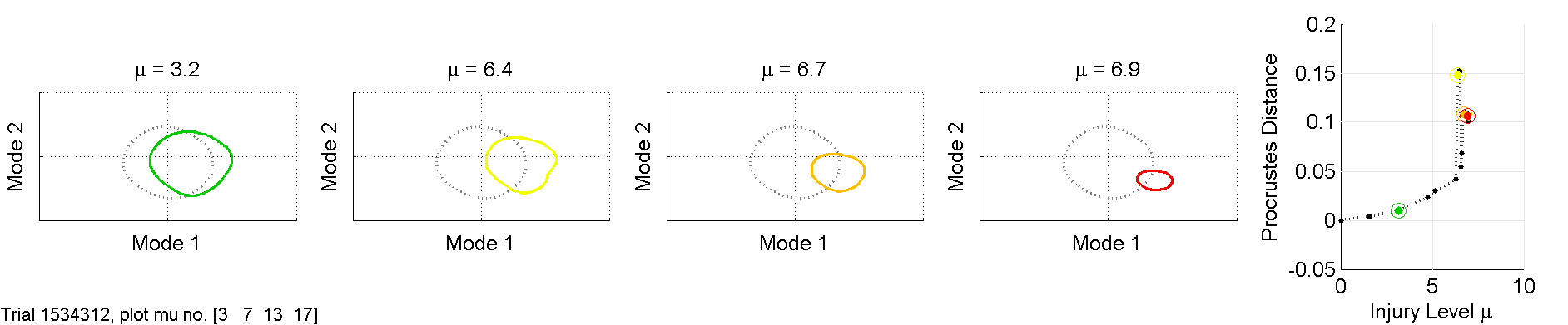

Supplement: S1 Figures — Figures similar to the rows of Fig 4, for all 1,447 trials conducted. (ZIP) [file pcbi.1005261.s002.zip › 1534312.png]

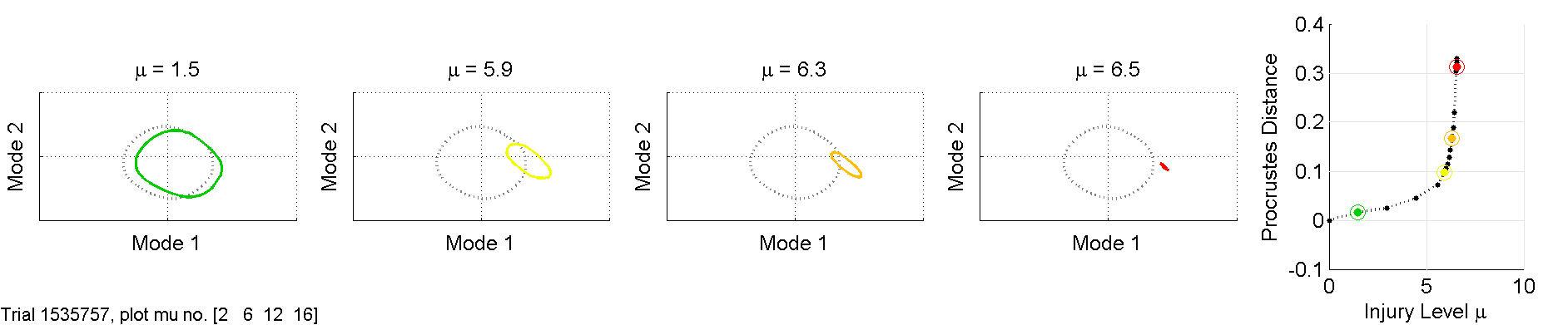

Supplement: S1 Figures — Figures similar to the rows of Fig 4, for all 1,447 trials conducted. (ZIP) [file pcbi.1005261.s002.zip › 1535757.png]

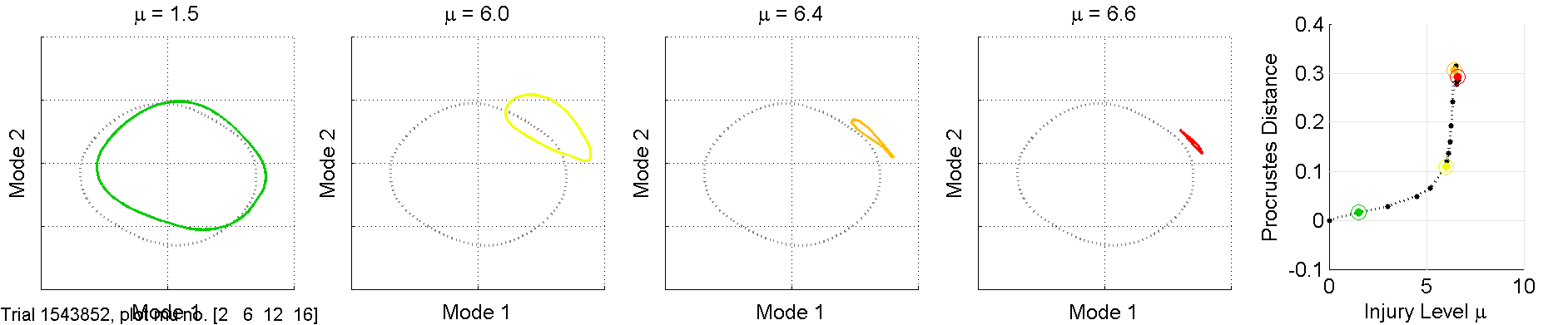

Supplement: S1 Figures — Figures similar to the rows of Fig 4, for all 1,447 trials conducted. (ZIP) [file pcbi.1005261.s002.zip › 1543852.png]

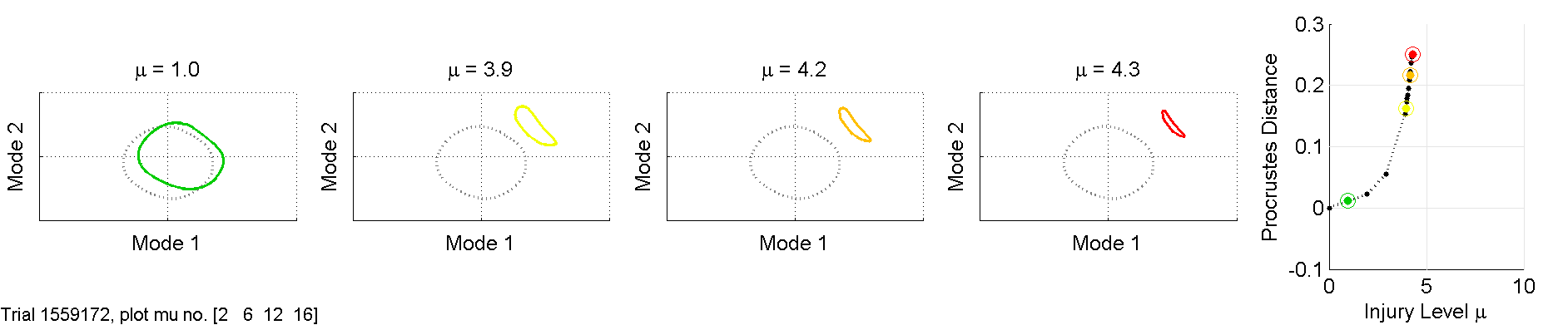

Supplement: S1 Figures — Figures similar to the rows of Fig 4, for all 1,447 trials conducted. (ZIP) [file pcbi.1005261.s002.zip › 1559172.png]

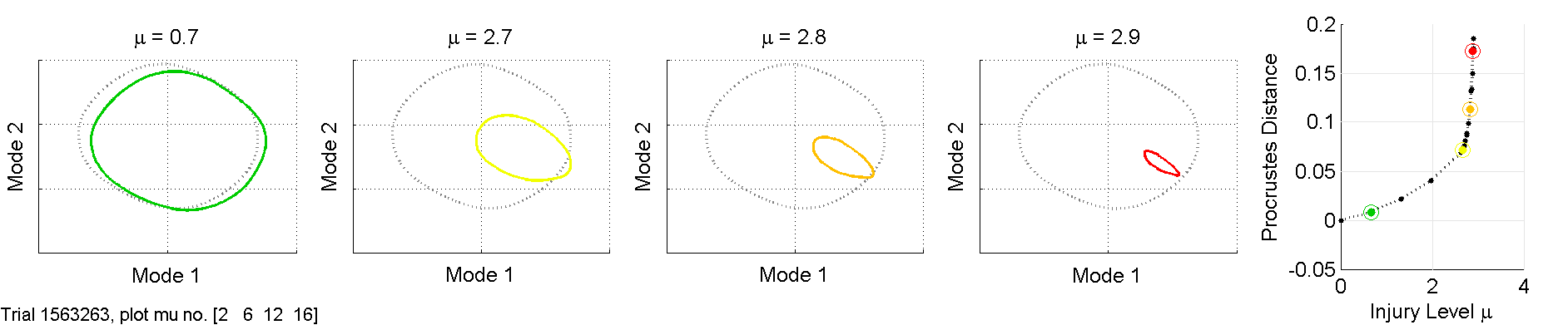

Supplement: S1 Figures — Figures similar to the rows of Fig 4, for all 1,447 trials conducted. (ZIP) [file pcbi.1005261.s002.zip › 1563263.png]

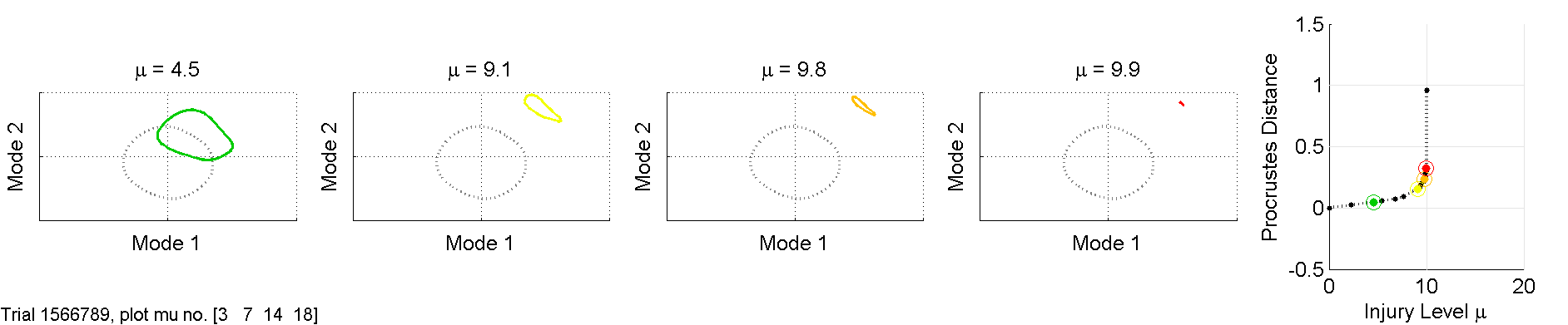

Supplement: S1 Figures — Figures similar to the rows of Fig 4, for all 1,447 trials conducted. (ZIP) [file pcbi.1005261.s002.zip › 1566789.png]

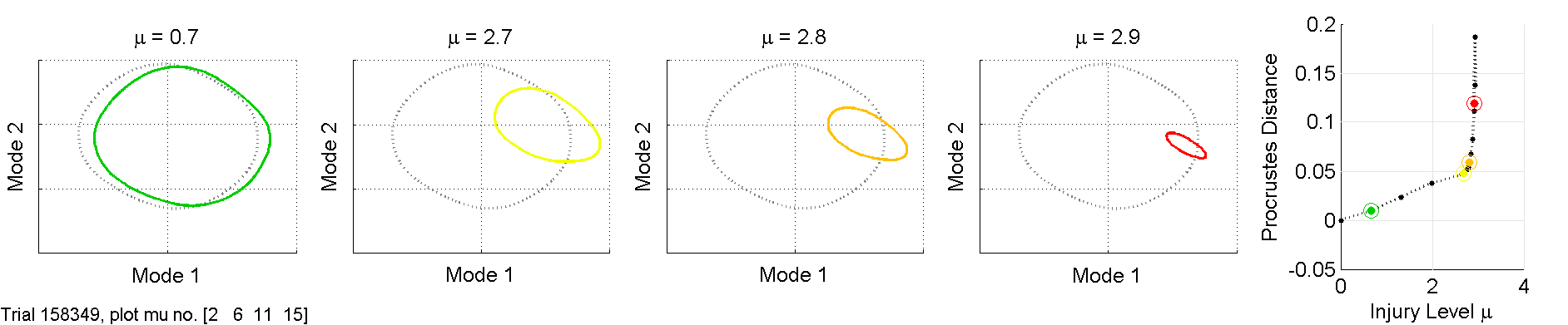

Supplement: S1 Figures — Figures similar to the rows of Fig 4, for all 1,447 trials conducted. (ZIP) [file pcbi.1005261.s002.zip › 158349.png]

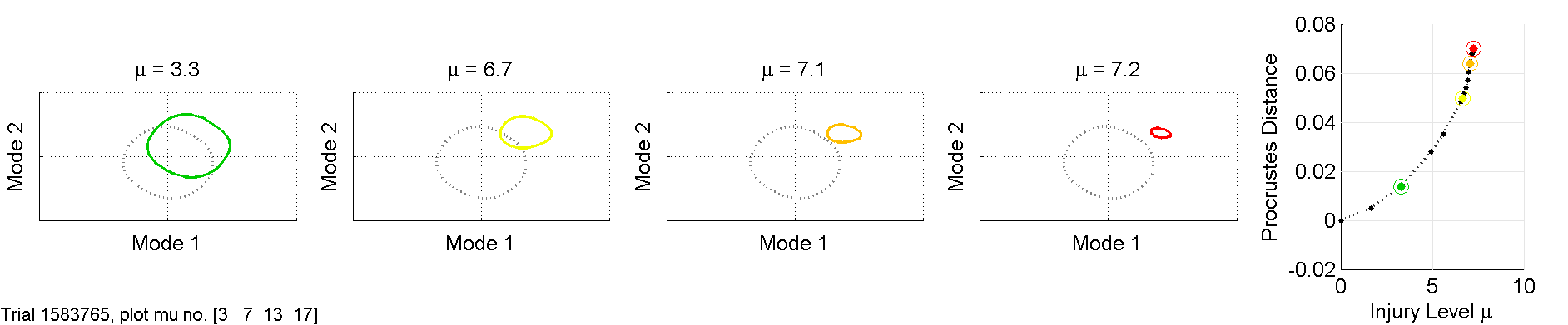

Supplement: S1 Figures — Figures similar to the rows of Fig 4, for all 1,447 trials conducted. (ZIP) [file pcbi.1005261.s002.zip › 1583765.png]

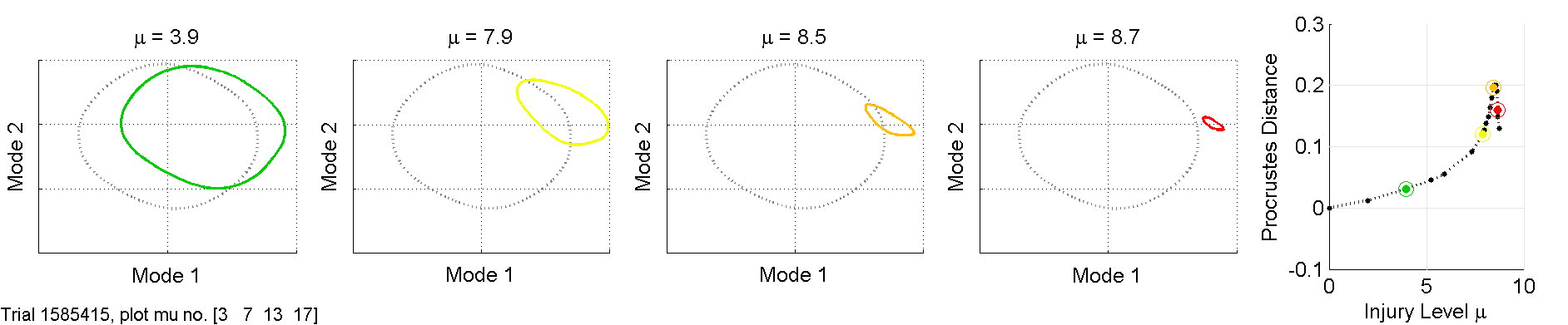

Supplement: S1 Figures — Figures similar to the rows of Fig 4, for all 1,447 trials conducted. (ZIP) [file pcbi.1005261.s002.zip › 1585415.png]

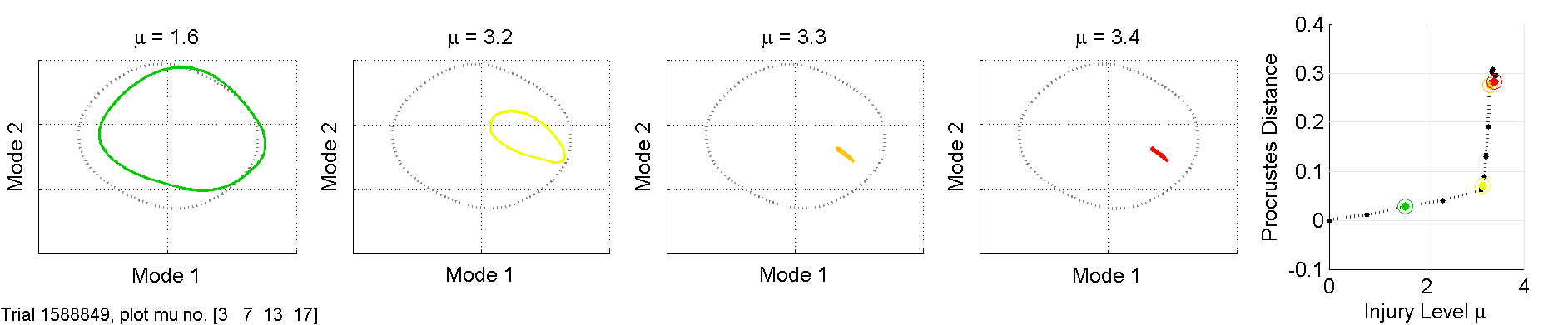

Supplement: S1 Figures — Figures similar to the rows of Fig 4, for all 1,447 trials conducted. (ZIP) [file pcbi.1005261.s002.zip › 1588849.png]

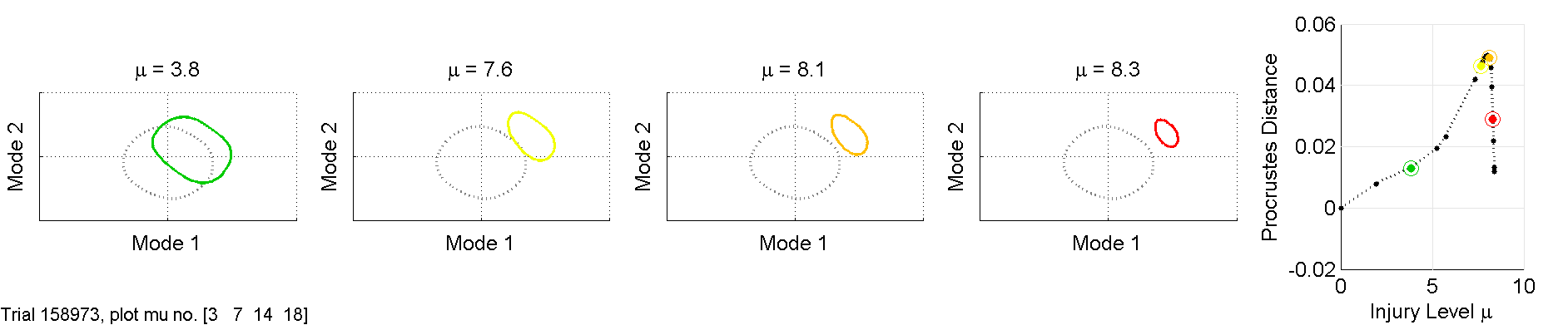

Supplement: S1 Figures — Figures similar to the rows of Fig 4, for all 1,447 trials conducted. (ZIP) [file pcbi.1005261.s002.zip › 158973.png]

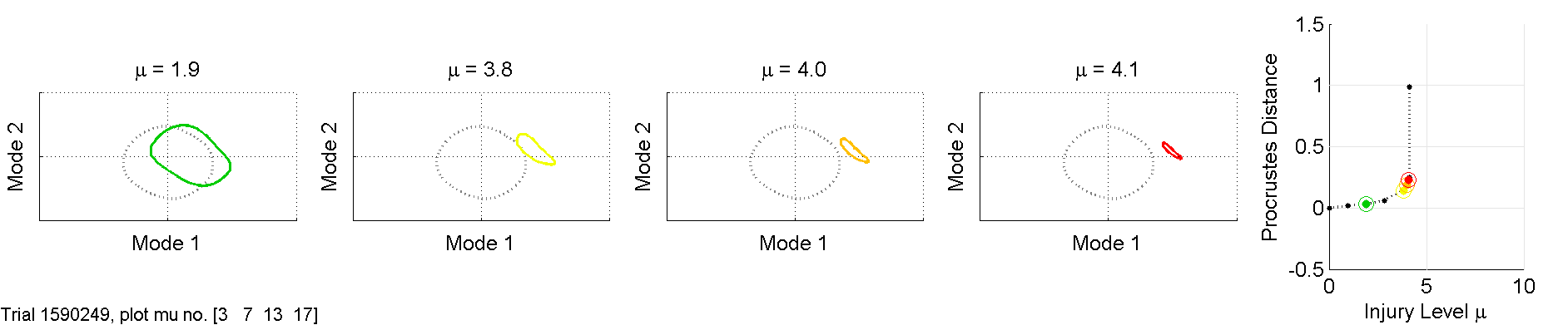

Supplement: S1 Figures — Figures similar to the rows of Fig 4, for all 1,447 trials conducted. (ZIP) [file pcbi.1005261.s002.zip › 1590249.png]

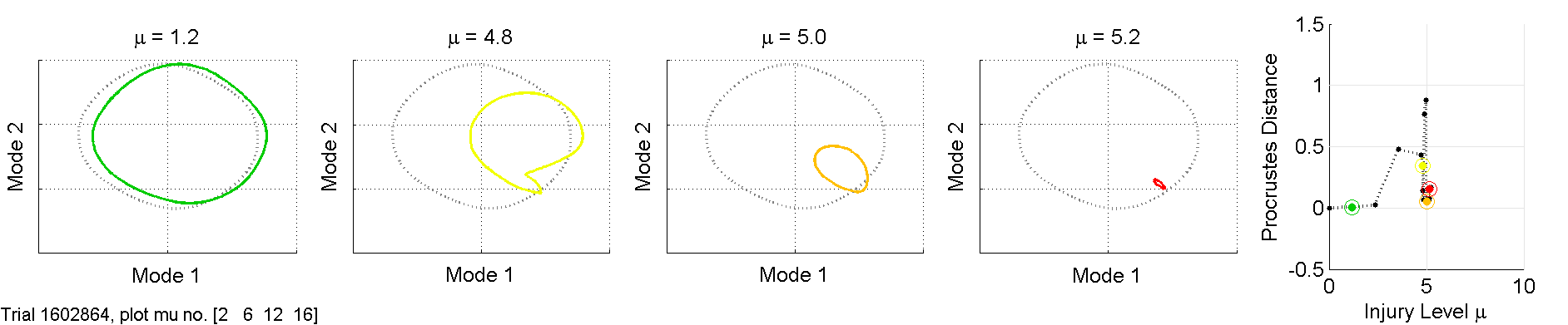

Supplement: S1 Figures — Figures similar to the rows of Fig 4, for all 1,447 trials conducted. (ZIP) [file pcbi.1005261.s002.zip › 1602864.png]

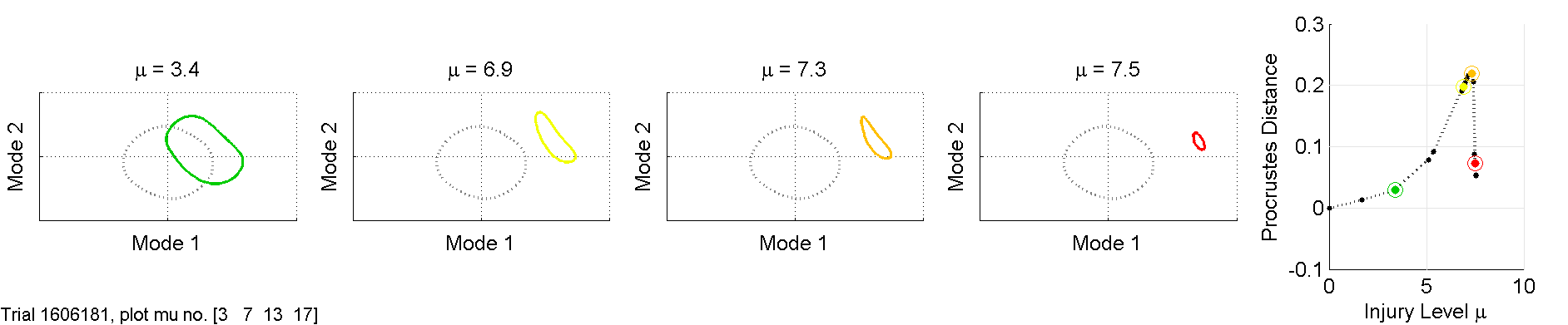

Supplement: S1 Figures — Figures similar to the rows of Fig 4, for all 1,447 trials conducted. (ZIP) [file pcbi.1005261.s002.zip › 1606181.png]

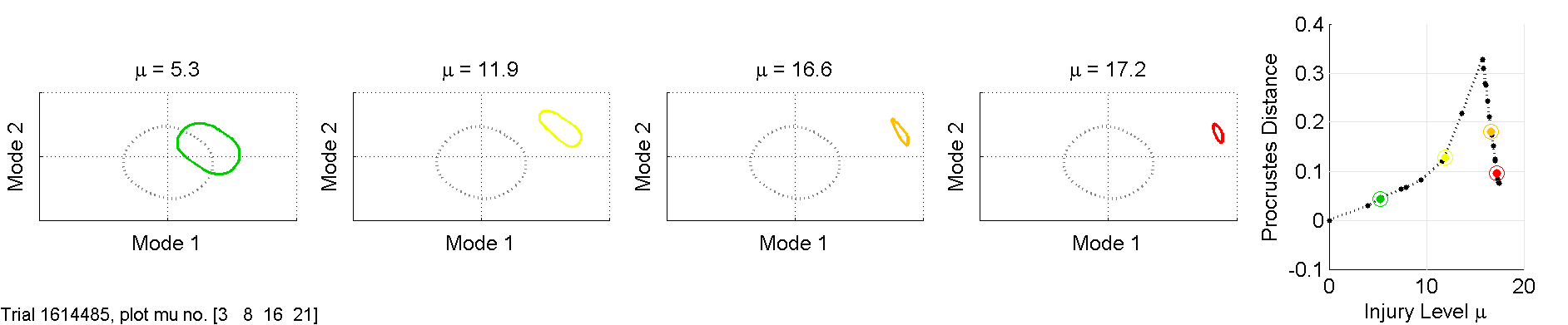

Supplement: S1 Figures — Figures similar to the rows of Fig 4, for all 1,447 trials conducted. (ZIP) [file pcbi.1005261.s002.zip › 1614485.png]

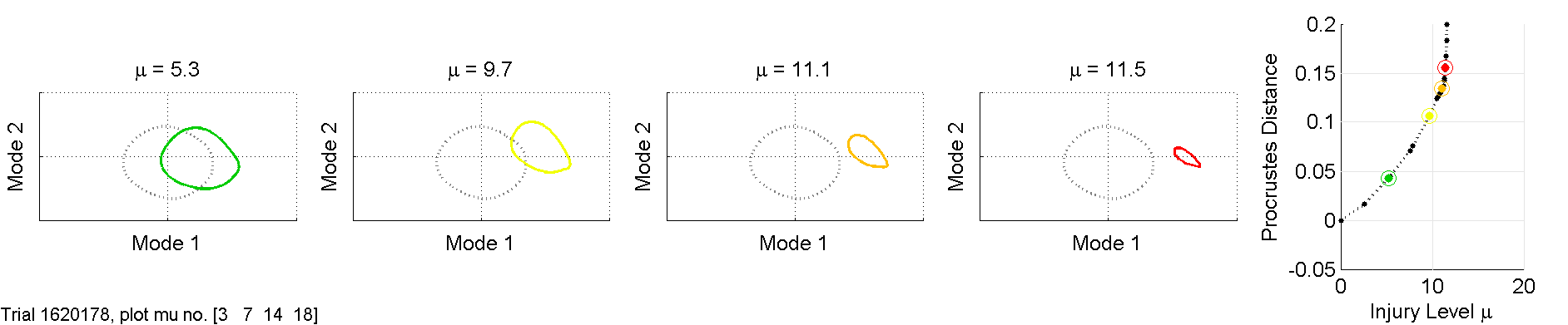

Supplement: S1 Figures — Figures similar to the rows of Fig 4, for all 1,447 trials conducted. (ZIP) [file pcbi.1005261.s002.zip › 1620178.png]

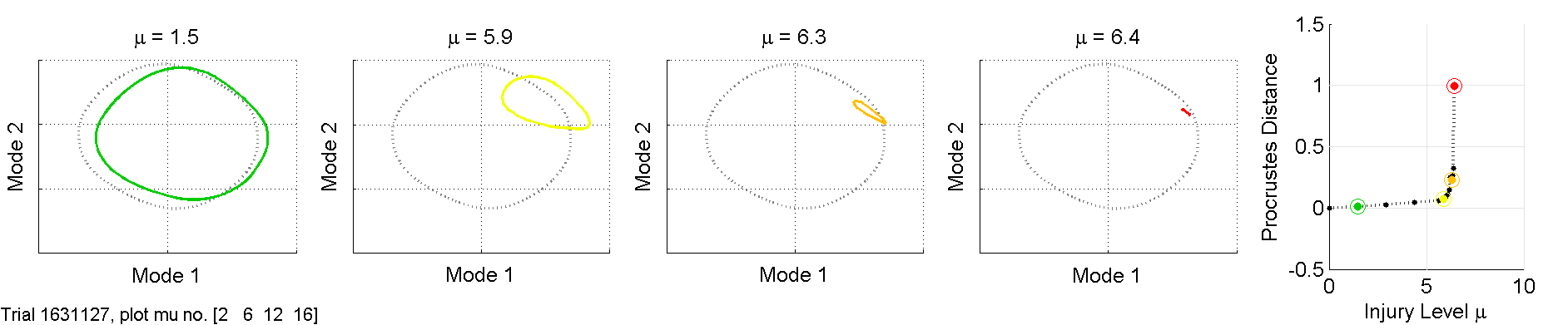

Supplement: S1 Figures — Figures similar to the rows of Fig 4, for all 1,447 trials conducted. (ZIP) [file pcbi.1005261.s002.zip › 1631127.png]

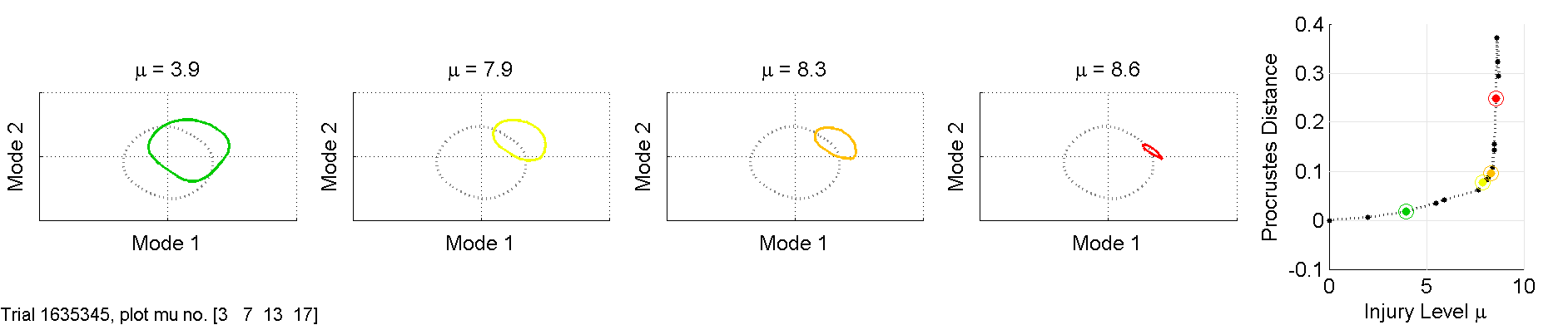

Supplement: S1 Figures — Figures similar to the rows of Fig 4, for all 1,447 trials conducted. (ZIP) [file pcbi.1005261.s002.zip › 1635345.png]

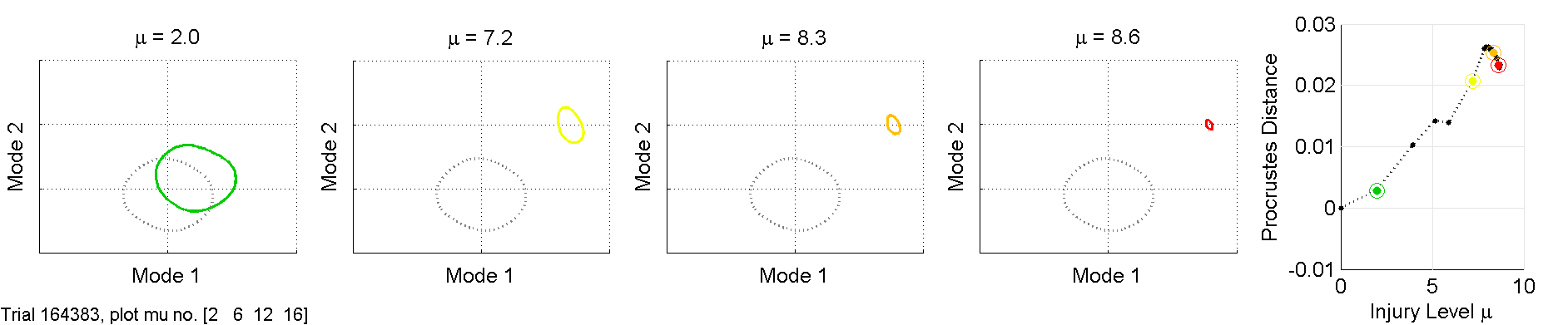

Supplement: S1 Figures — Figures similar to the rows of Fig 4, for all 1,447 trials conducted. (ZIP) [file pcbi.1005261.s002.zip › 164383.png]

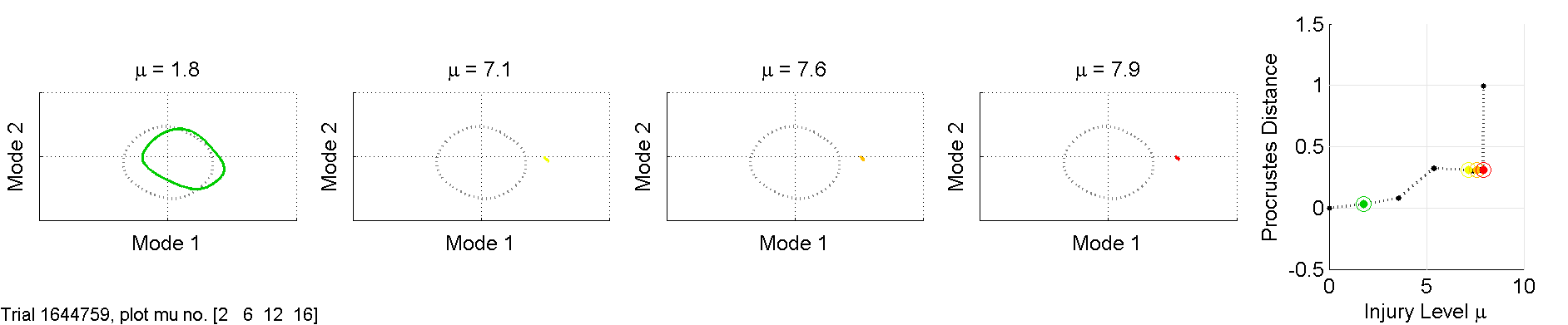

Supplement: S1 Figures — Figures similar to the rows of Fig 4, for all 1,447 trials conducted. (ZIP) [file pcbi.1005261.s002.zip › 1644759.png]
