# Supplementary figures and images for: Functionality and Robustness of Injured Connectomic Dynamics in C. elegans: Linking Behavioral Deficits to Neural Circuit Damage (part 3 of 3)
Source: PLoS Comput Biol. 2017 Jan 5;13(1):e1005261. doi: 10.1371/journal.pcbi.1005261 (PMC5215891; doi:10.1371/journal.pcbi.1005261)

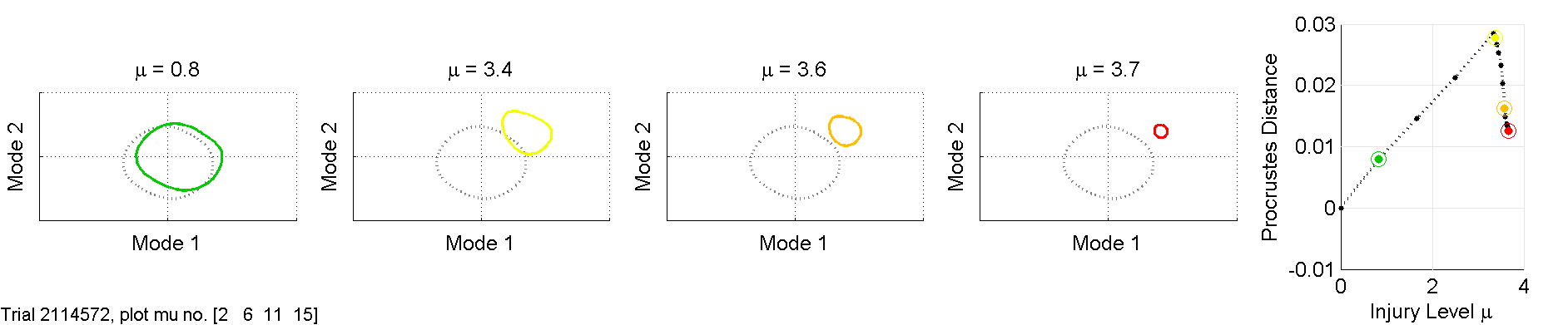

Supplement: S1 Figures — Figures similar to the rows of Fig 4, for all 1,447 trials conducted. (ZIP) [file pcbi.1005261.s002.zip › 2114572.png]
