# Supplementary figures and images for: Functionality and Robustness of Injured Connectomic Dynamics in C. elegans: Linking Behavioral Deficits to Neural Circuit Damage (part 2 of 3)
Source: PLoS Comput Biol. 2017 Jan 5;13(1):e1005261. doi: 10.1371/journal.pcbi.1005261 (PMC5215891; doi:10.1371/journal.pcbi.1005261)

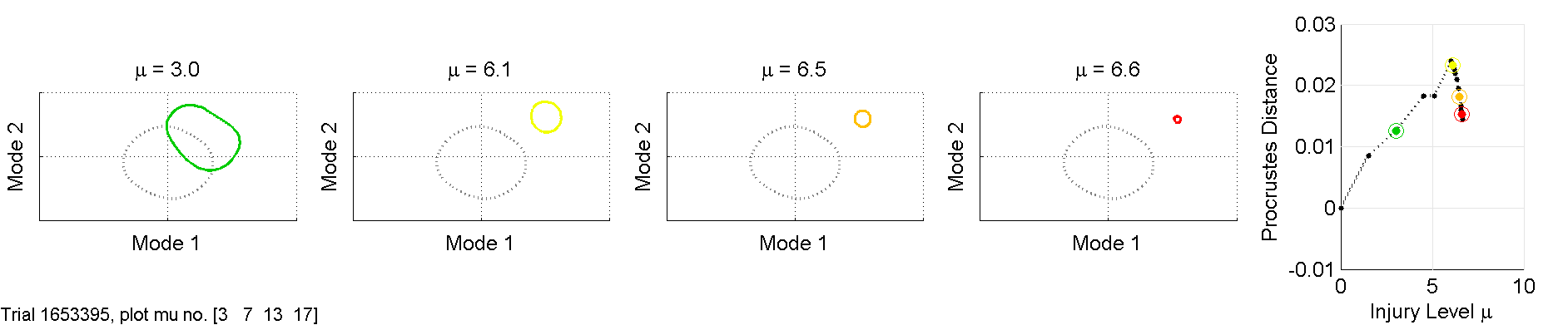

Supplement: S1 Figures — Figures similar to the rows of Fig 4, for all 1,447 trials conducted. (ZIP) [file pcbi.1005261.s002.zip › 1653395.png]

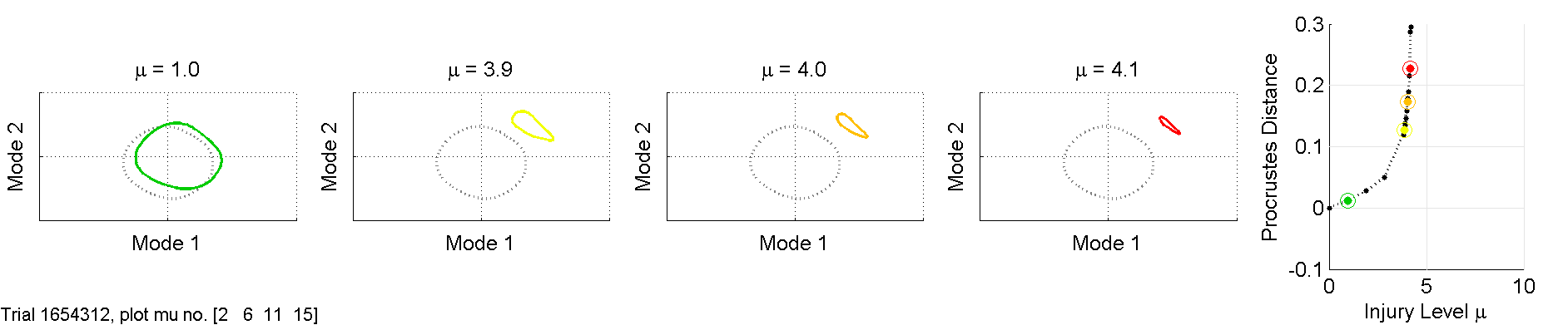

Supplement: S1 Figures — Figures similar to the rows of Fig 4, for all 1,447 trials conducted. (ZIP) [file pcbi.1005261.s002.zip › 1654312.png]

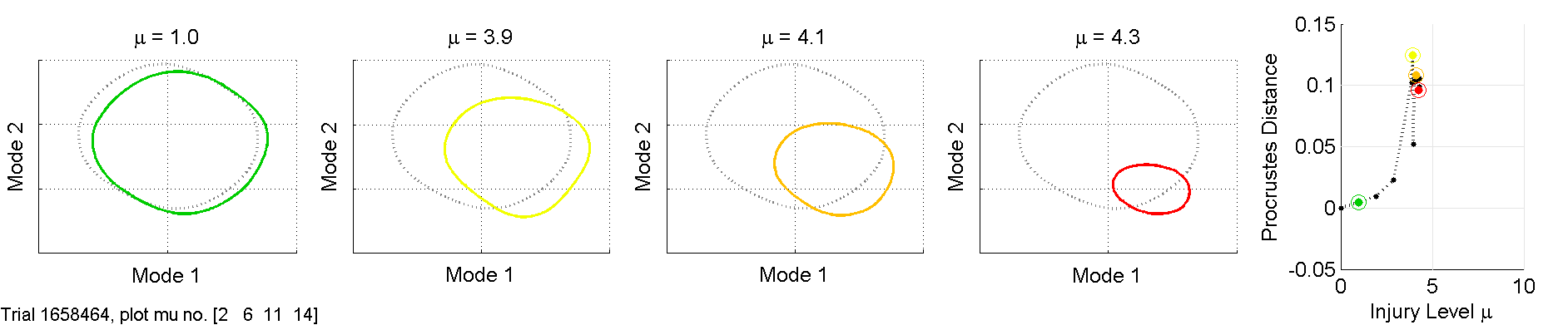

Supplement: S1 Figures — Figures similar to the rows of Fig 4, for all 1,447 trials conducted. (ZIP) [file pcbi.1005261.s002.zip › 1658464.png]

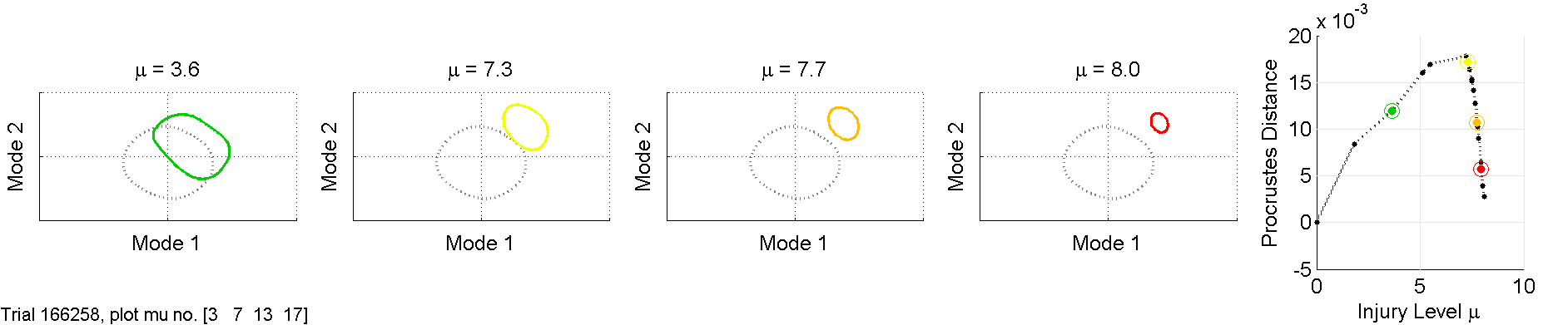

Supplement: S1 Figures — Figures similar to the rows of Fig 4, for all 1,447 trials conducted. (ZIP) [file pcbi.1005261.s002.zip › 166258.png]

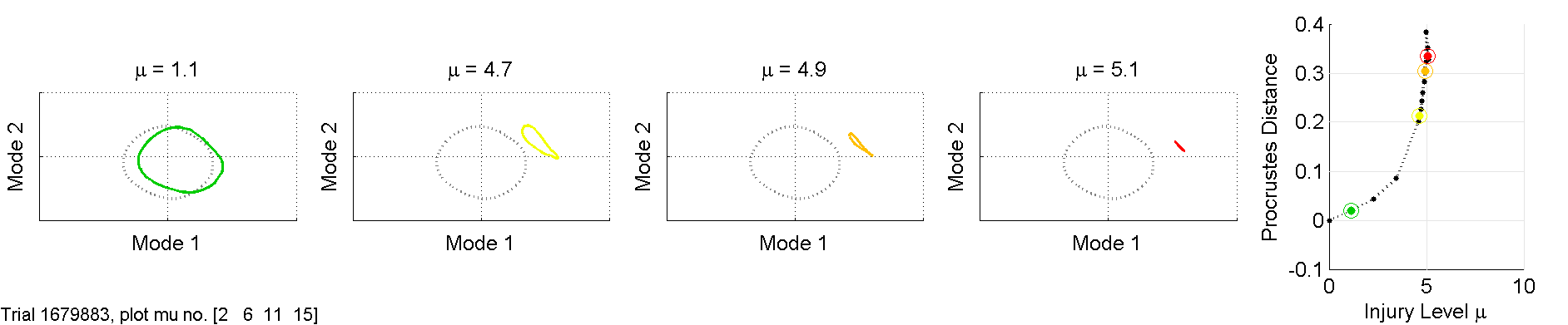

Supplement: S1 Figures — Figures similar to the rows of Fig 4, for all 1,447 trials conducted. (ZIP) [file pcbi.1005261.s002.zip › 1679883.png]

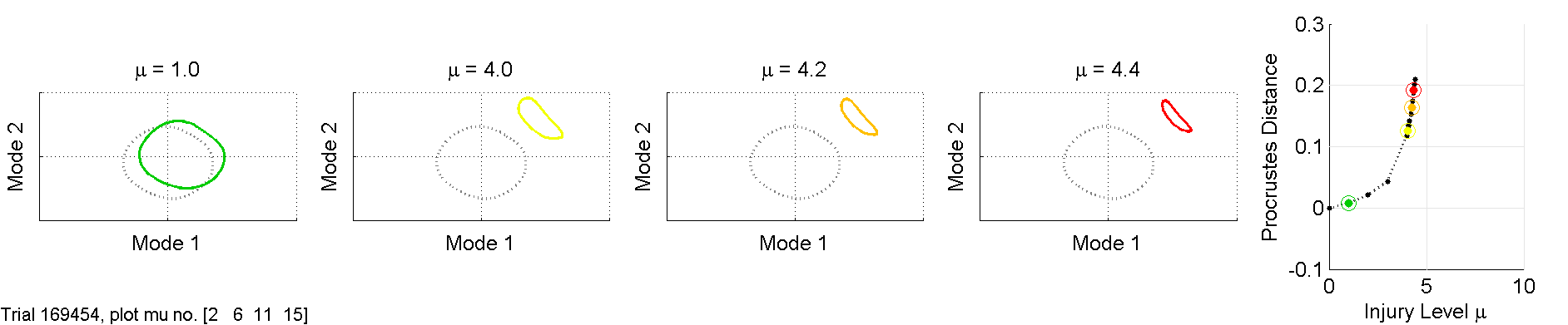

Supplement: S1 Figures — Figures similar to the rows of Fig 4, for all 1,447 trials conducted. (ZIP) [file pcbi.1005261.s002.zip › 169454.png]

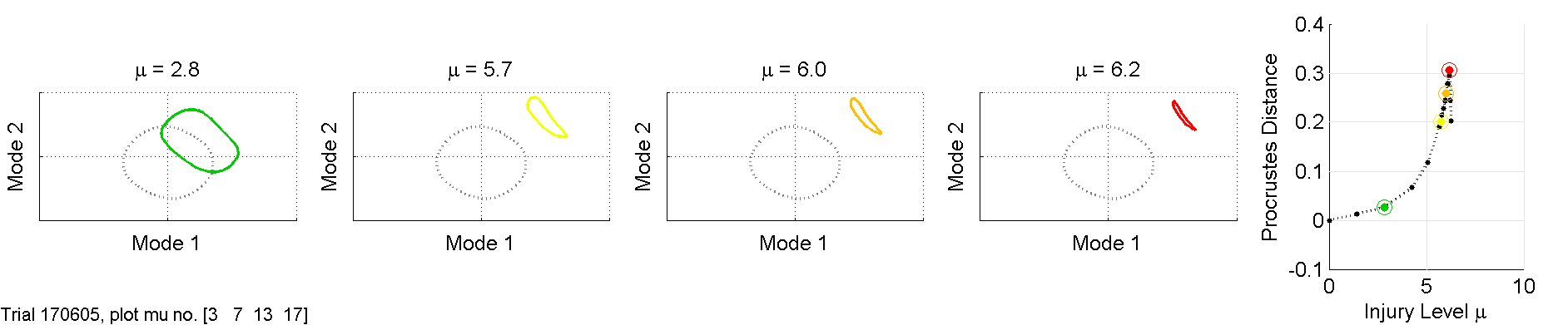

Supplement: S1 Figures — Figures similar to the rows of Fig 4, for all 1,447 trials conducted. (ZIP) [file pcbi.1005261.s002.zip › 170605.png]

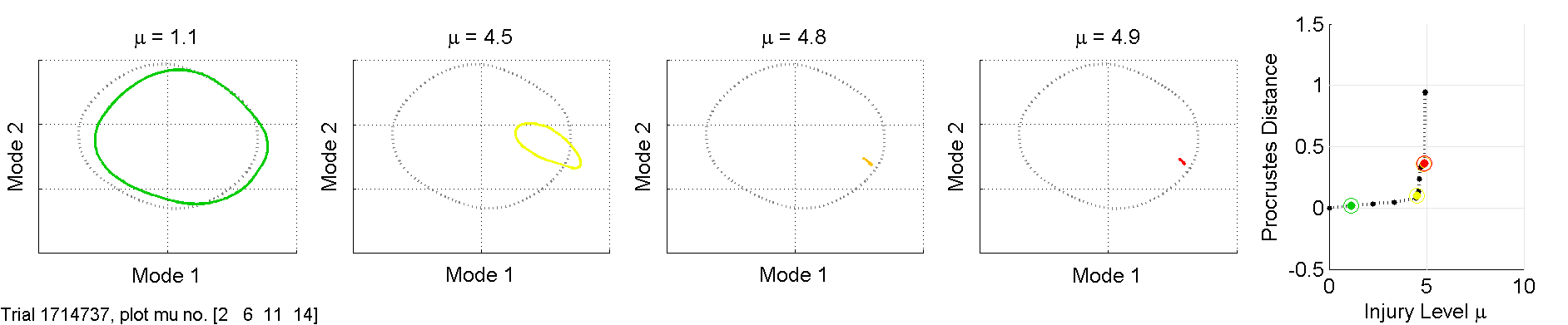

Supplement: S1 Figures — Figures similar to the rows of Fig 4, for all 1,447 trials conducted. (ZIP) [file pcbi.1005261.s002.zip › 1714737.png]

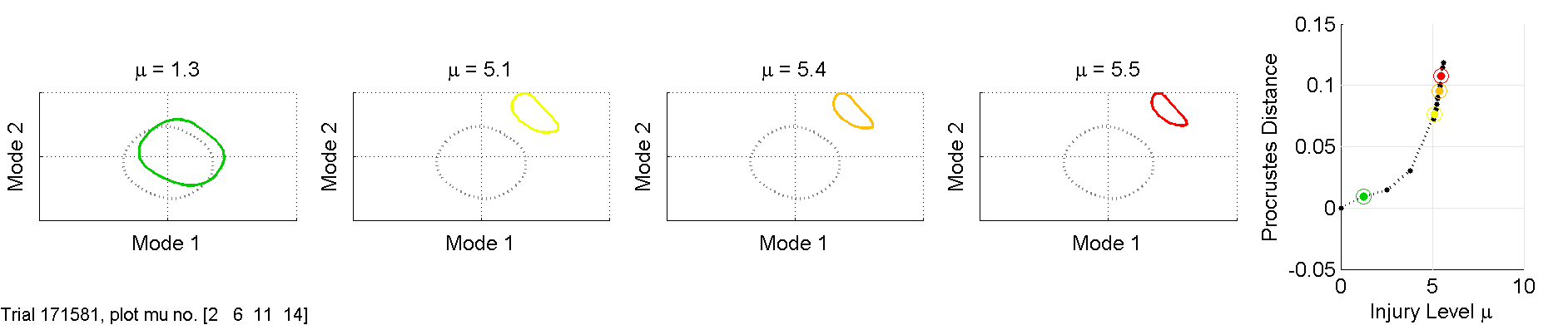

Supplement: S1 Figures — Figures similar to the rows of Fig 4, for all 1,447 trials conducted. (ZIP) [file pcbi.1005261.s002.zip › 171581.png]

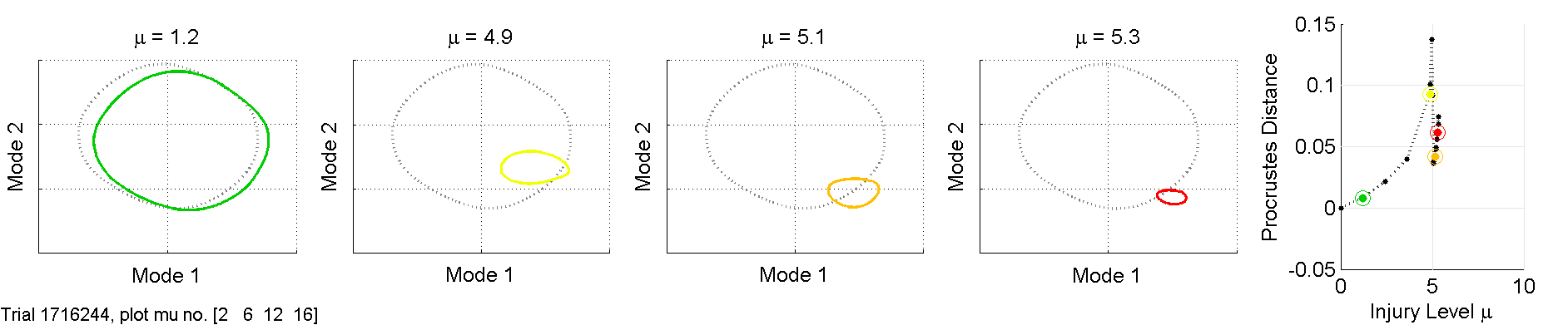

Supplement: S1 Figures — Figures similar to the rows of Fig 4, for all 1,447 trials conducted. (ZIP) [file pcbi.1005261.s002.zip › 1716244.png]

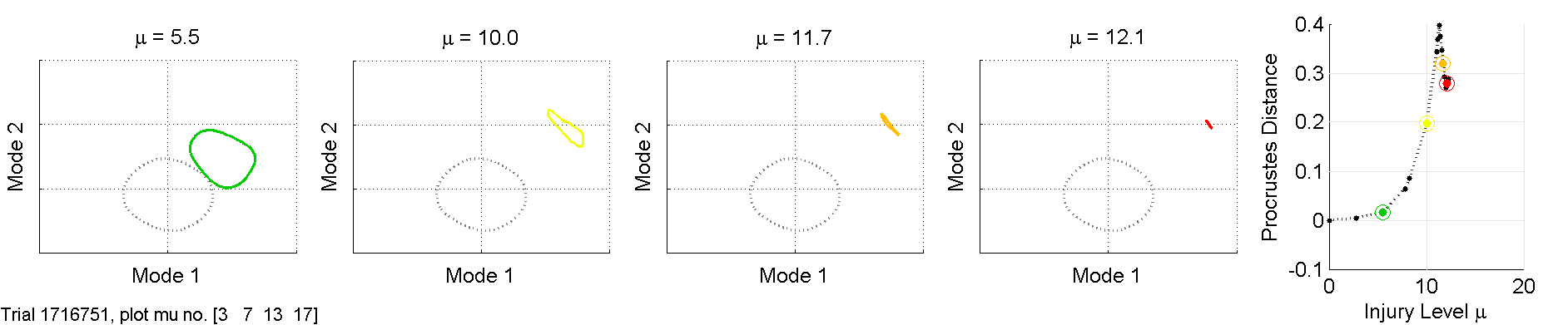

Supplement: S1 Figures — Figures similar to the rows of Fig 4, for all 1,447 trials conducted. (ZIP) [file pcbi.1005261.s002.zip › 1716751.png]

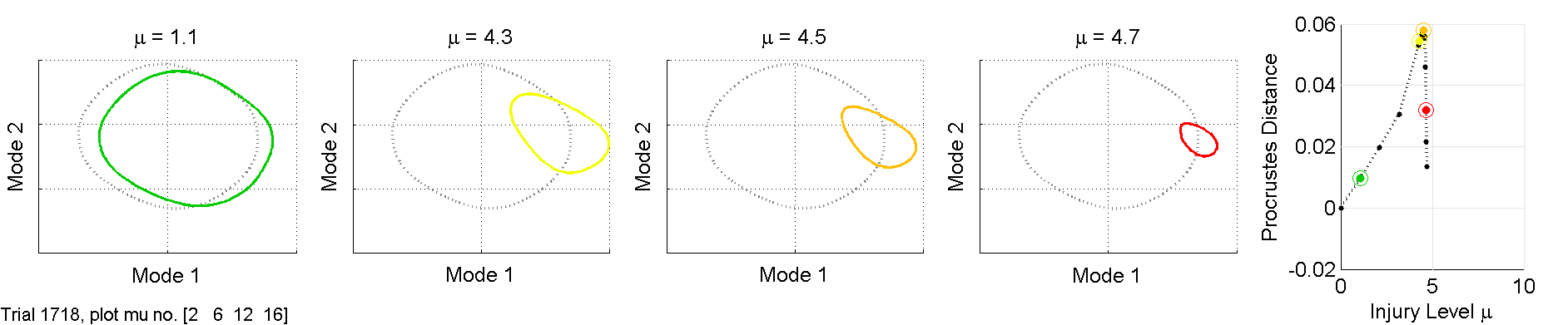

Supplement: S1 Figures — Figures similar to the rows of Fig 4, for all 1,447 trials conducted. (ZIP) [file pcbi.1005261.s002.zip › 1718.png]

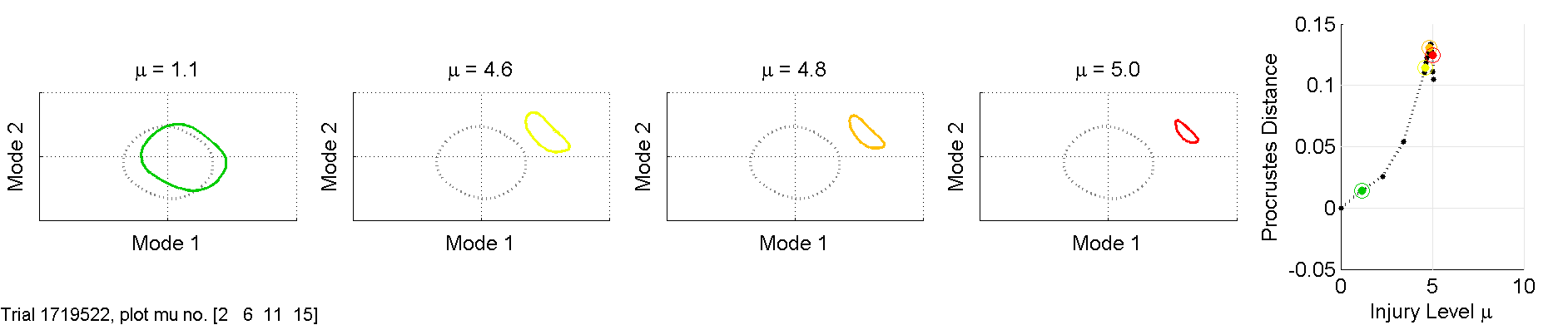

Supplement: S1 Figures — Figures similar to the rows of Fig 4, for all 1,447 trials conducted. (ZIP) [file pcbi.1005261.s002.zip › 1719522.png]

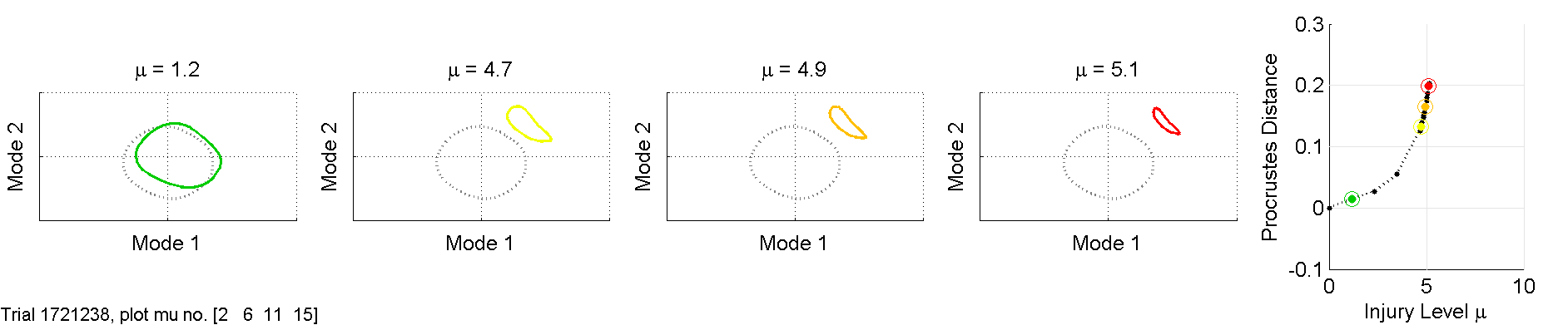

Supplement: S1 Figures — Figures similar to the rows of Fig 4, for all 1,447 trials conducted. (ZIP) [file pcbi.1005261.s002.zip › 1721238.png]

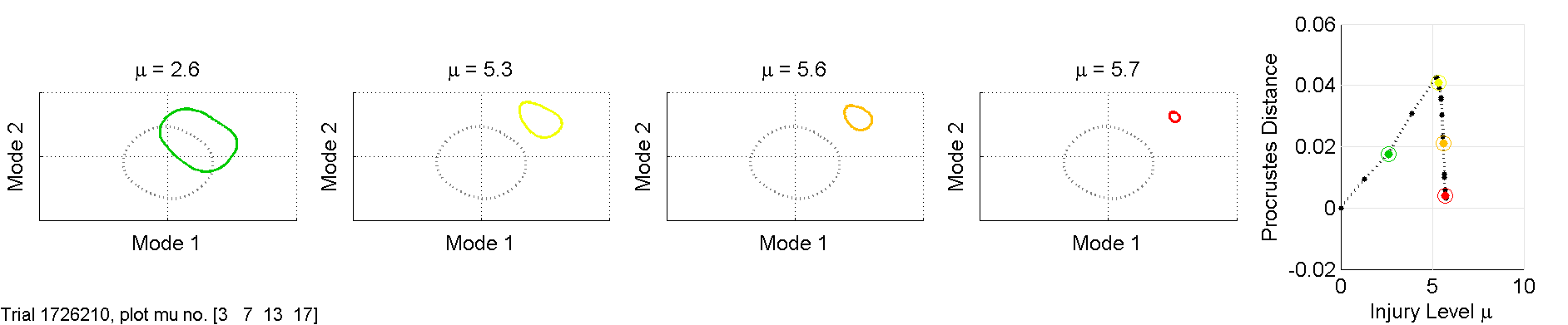

Supplement: S1 Figures — Figures similar to the rows of Fig 4, for all 1,447 trials conducted. (ZIP) [file pcbi.1005261.s002.zip › 1726210.png]

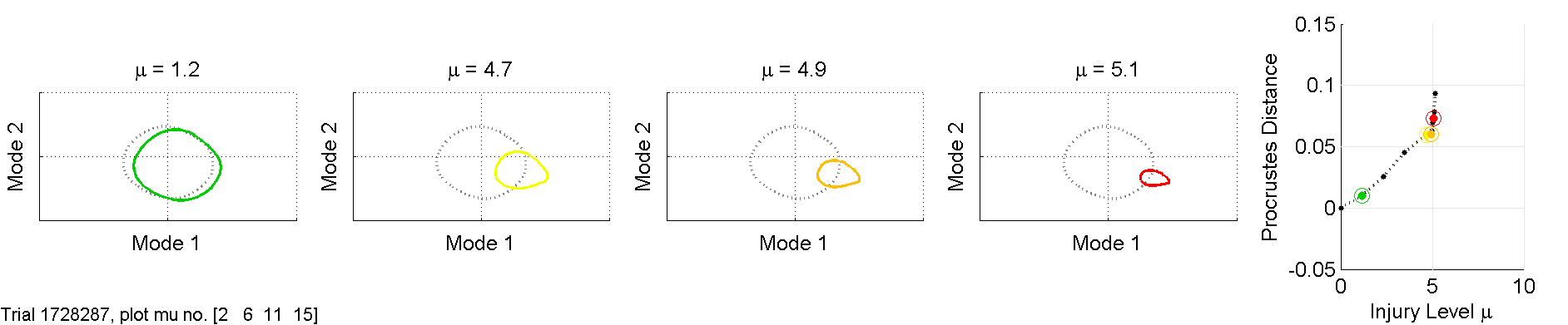

Supplement: S1 Figures — Figures similar to the rows of Fig 4, for all 1,447 trials conducted. (ZIP) [file pcbi.1005261.s002.zip › 1728287.png]

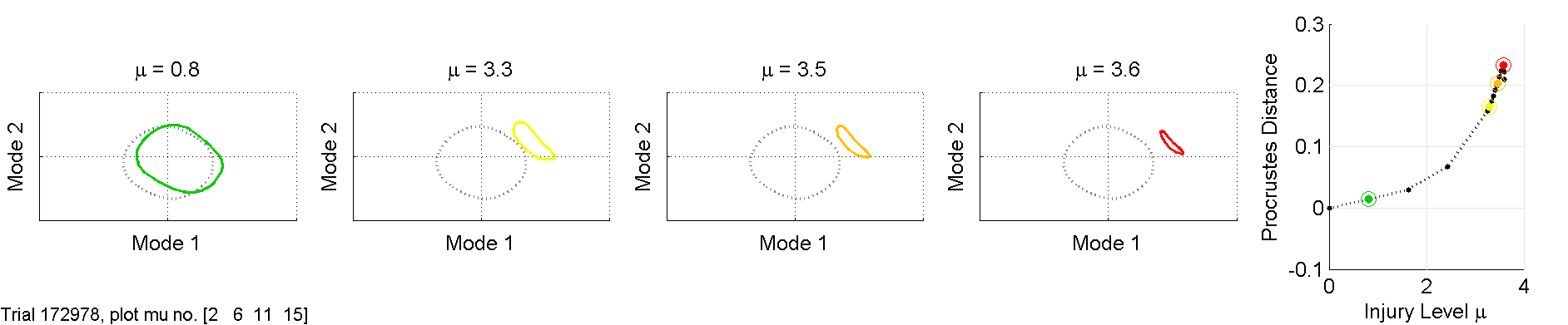

Supplement: S1 Figures — Figures similar to the rows of Fig 4, for all 1,447 trials conducted. (ZIP) [file pcbi.1005261.s002.zip › 172978.png]

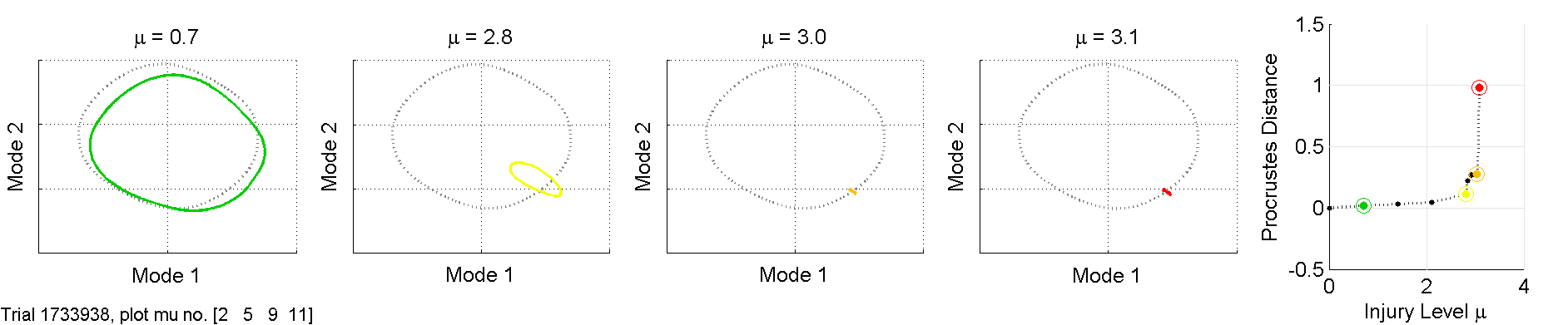

Supplement: S1 Figures — Figures similar to the rows of Fig 4, for all 1,447 trials conducted. (ZIP) [file pcbi.1005261.s002.zip › 1733938.png]

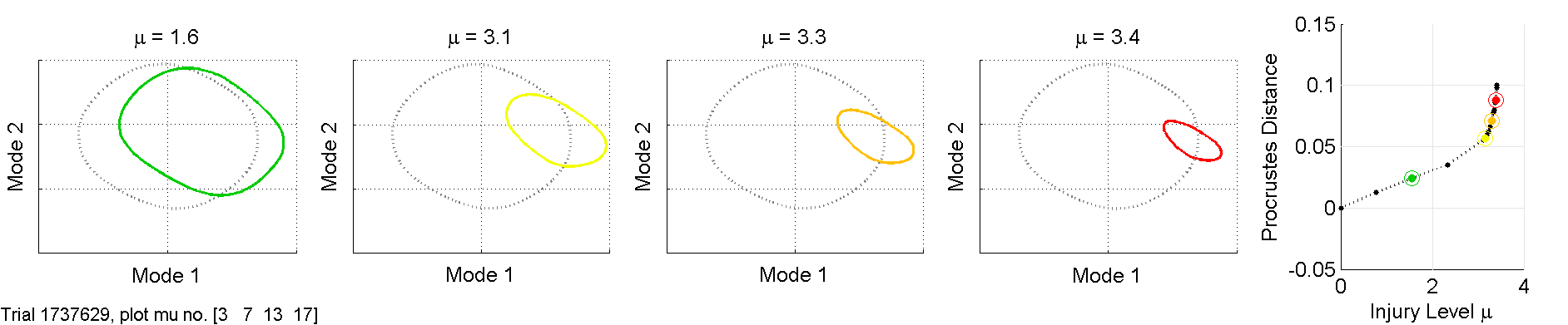

Supplement: S1 Figures — Figures similar to the rows of Fig 4, for all 1,447 trials conducted. (ZIP) [file pcbi.1005261.s002.zip › 1737629.png]

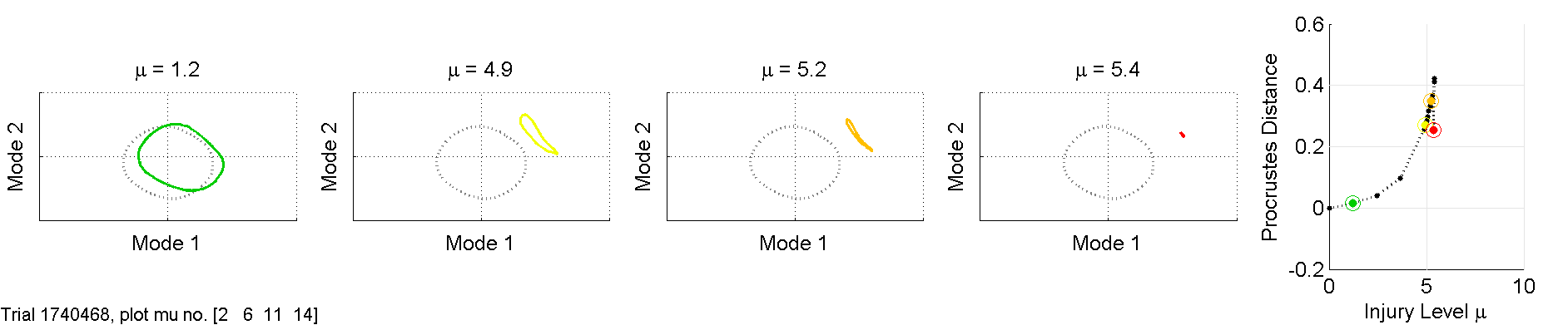

Supplement: S1 Figures — Figures similar to the rows of Fig 4, for all 1,447 trials conducted. (ZIP) [file pcbi.1005261.s002.zip › 1740468.png]

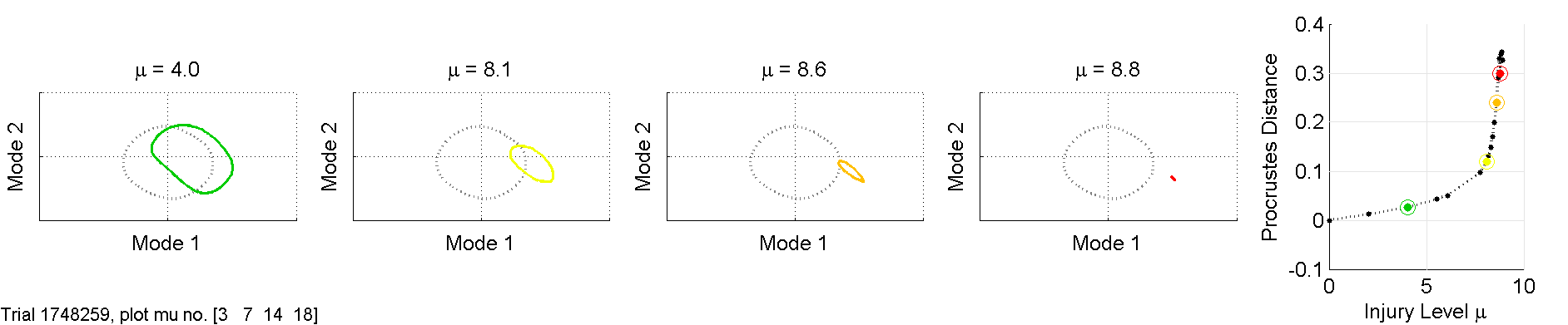

Supplement: S1 Figures — Figures similar to the rows of Fig 4, for all 1,447 trials conducted. (ZIP) [file pcbi.1005261.s002.zip › 1748259.png]

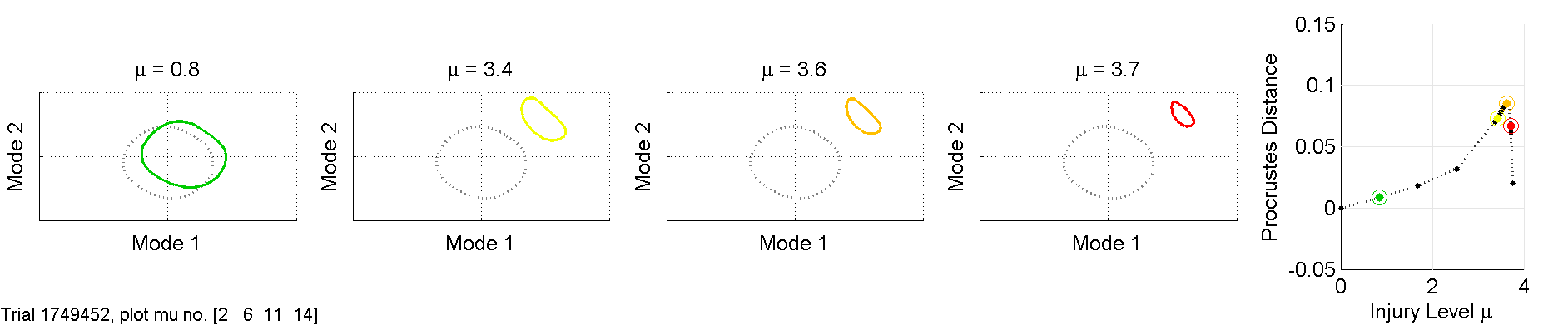

Supplement: S1 Figures — Figures similar to the rows of Fig 4, for all 1,447 trials conducted. (ZIP) [file pcbi.1005261.s002.zip › 1749452.png]

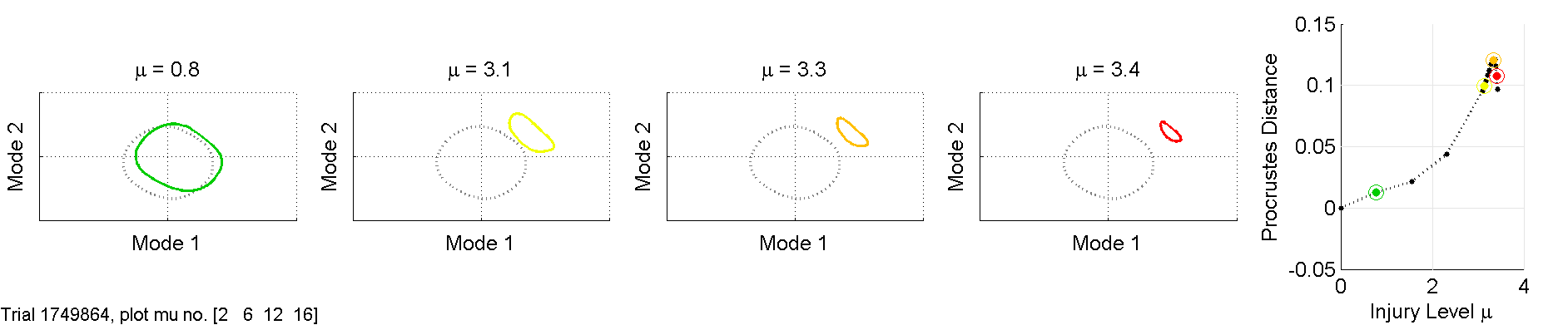

Supplement: S1 Figures — Figures similar to the rows of Fig 4, for all 1,447 trials conducted. (ZIP) [file pcbi.1005261.s002.zip › 1749864.png]

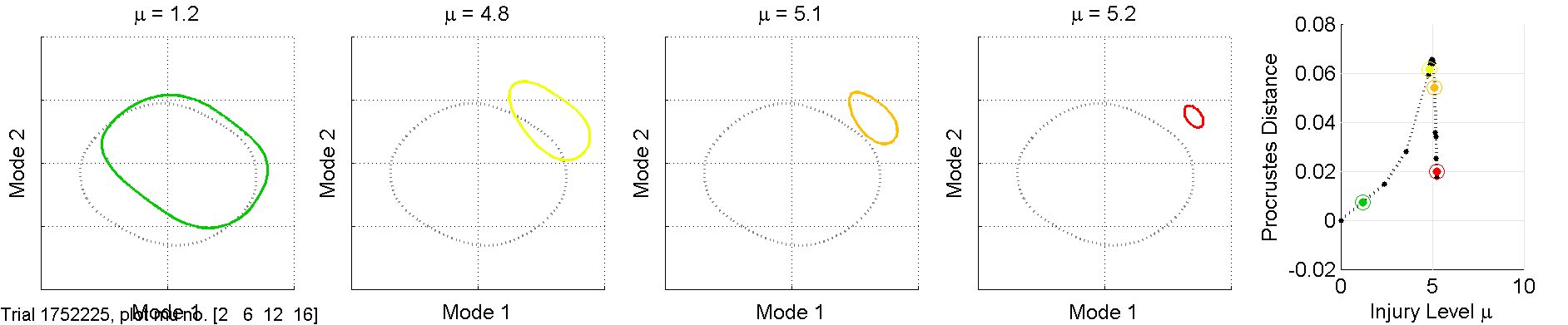

Supplement: S1 Figures — Figures similar to the rows of Fig 4, for all 1,447 trials conducted. (ZIP) [file pcbi.1005261.s002.zip › 1752225.png]

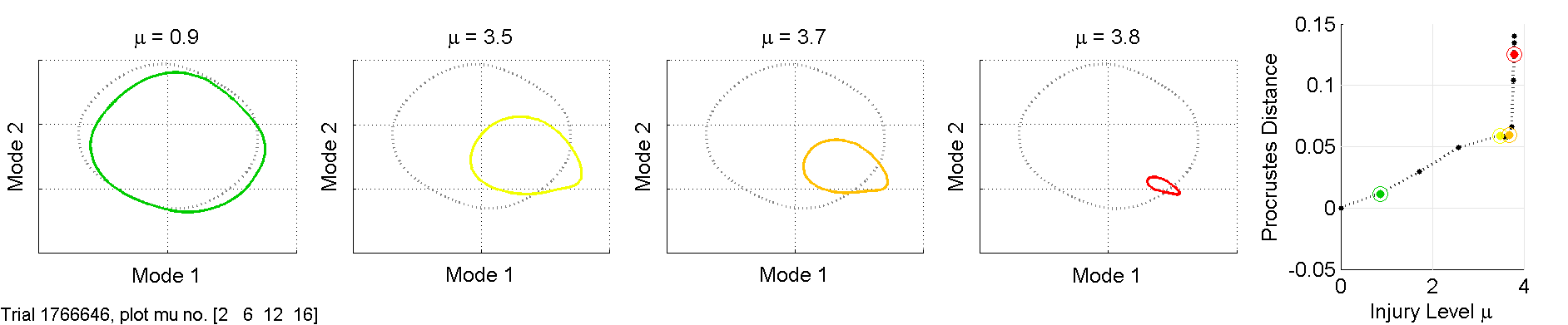

Supplement: S1 Figures — Figures similar to the rows of Fig 4, for all 1,447 trials conducted. (ZIP) [file pcbi.1005261.s002.zip › 1766646.png]

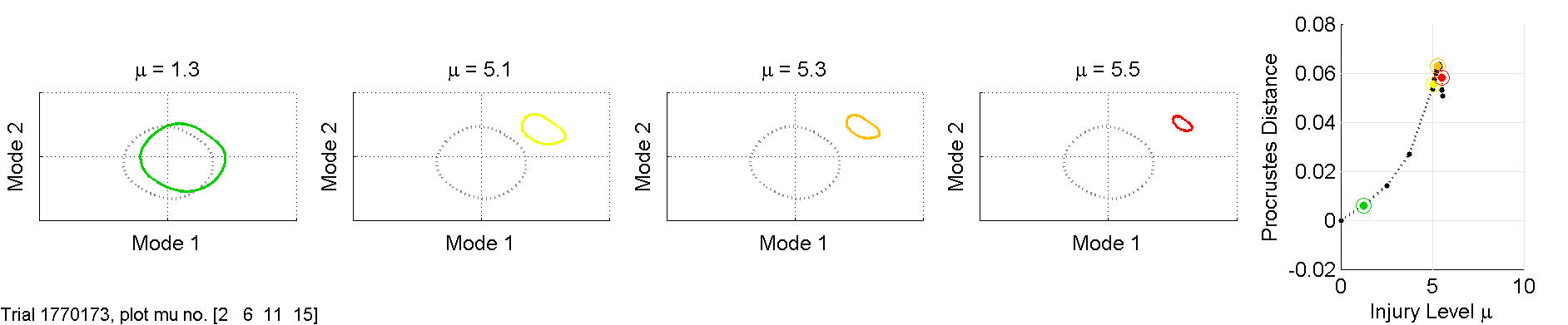

Supplement: S1 Figures — Figures similar to the rows of Fig 4, for all 1,447 trials conducted. (ZIP) [file pcbi.1005261.s002.zip › 1770173.png]

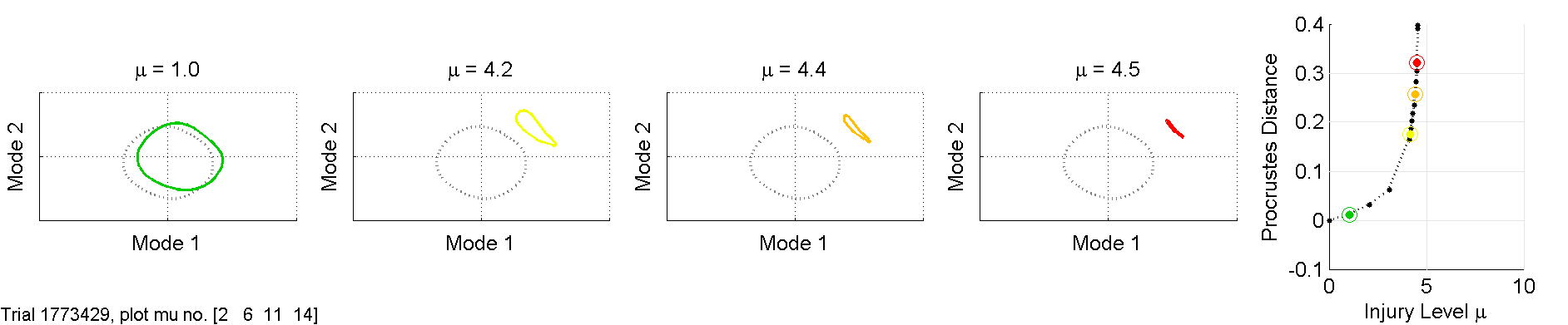

Supplement: S1 Figures — Figures similar to the rows of Fig 4, for all 1,447 trials conducted. (ZIP) [file pcbi.1005261.s002.zip › 1773429.png]

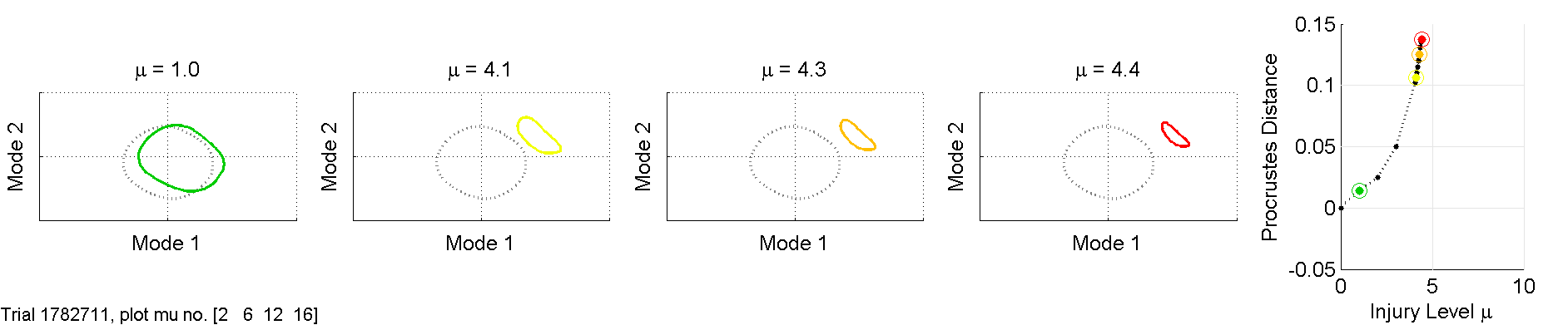

Supplement: S1 Figures — Figures similar to the rows of Fig 4, for all 1,447 trials conducted. (ZIP) [file pcbi.1005261.s002.zip › 1782711.png]

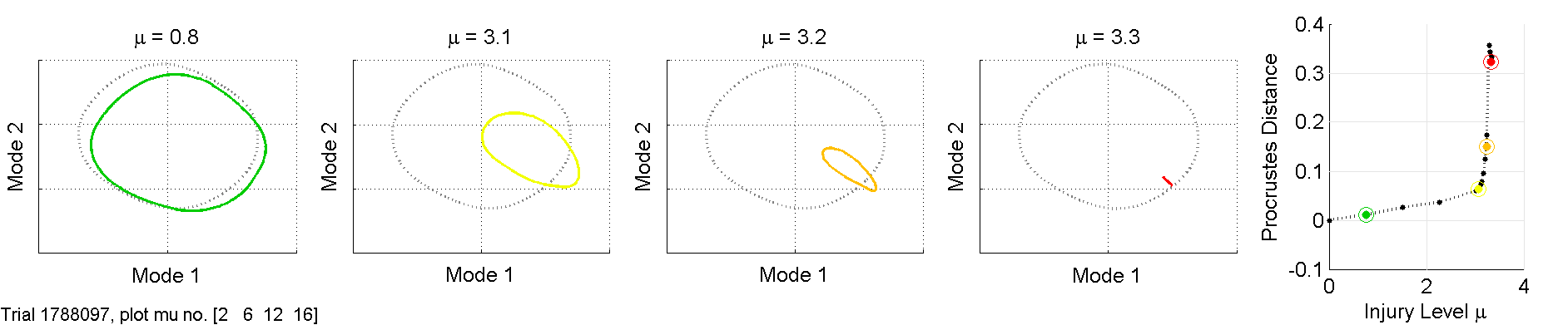

Supplement: S1 Figures — Figures similar to the rows of Fig 4, for all 1,447 trials conducted. (ZIP) [file pcbi.1005261.s002.zip › 1788097.png]

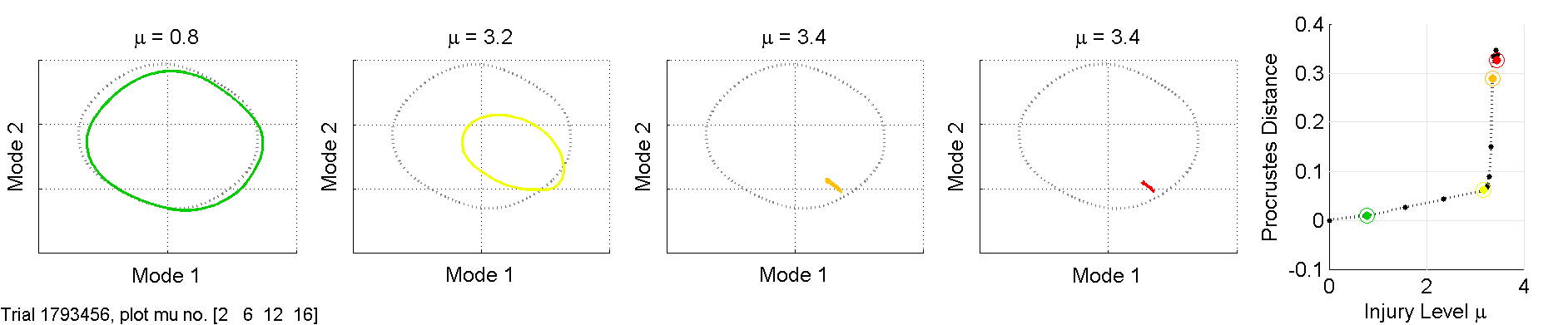

Supplement: S1 Figures — Figures similar to the rows of Fig 4, for all 1,447 trials conducted. (ZIP) [file pcbi.1005261.s002.zip › 1793456.png]

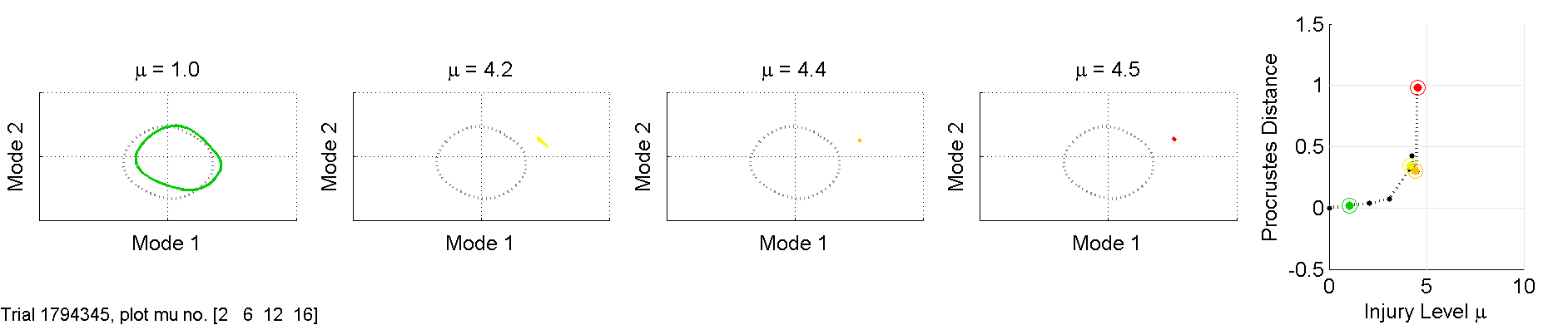

Supplement: S1 Figures — Figures similar to the rows of Fig 4, for all 1,447 trials conducted. (ZIP) [file pcbi.1005261.s002.zip › 1794345.png]

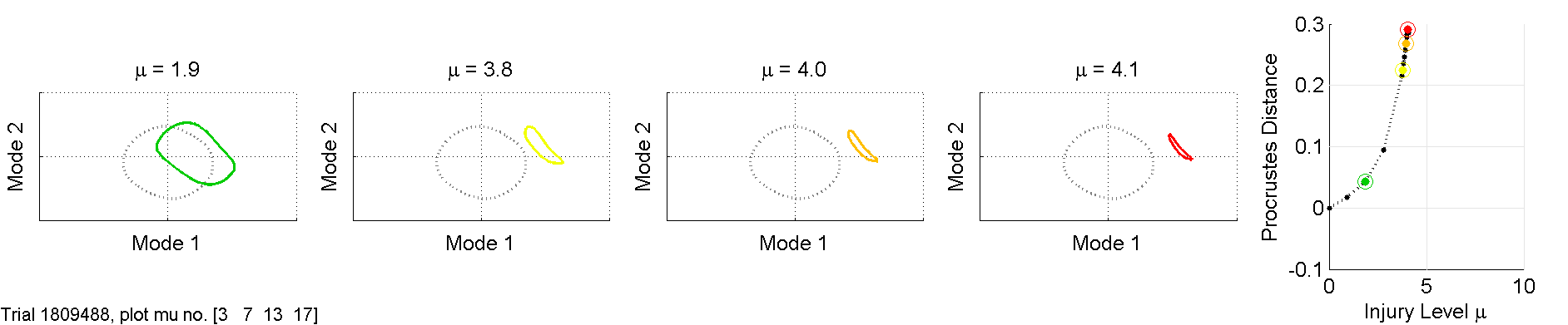

Supplement: S1 Figures — Figures similar to the rows of Fig 4, for all 1,447 trials conducted. (ZIP) [file pcbi.1005261.s002.zip › 1809488.png]

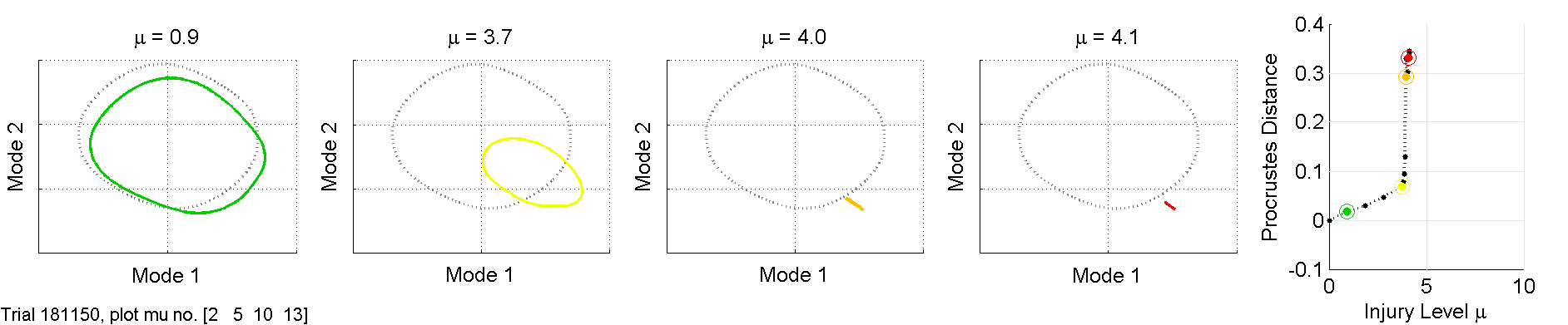

Supplement: S1 Figures — Figures similar to the rows of Fig 4, for all 1,447 trials conducted. (ZIP) [file pcbi.1005261.s002.zip › 181150.png]

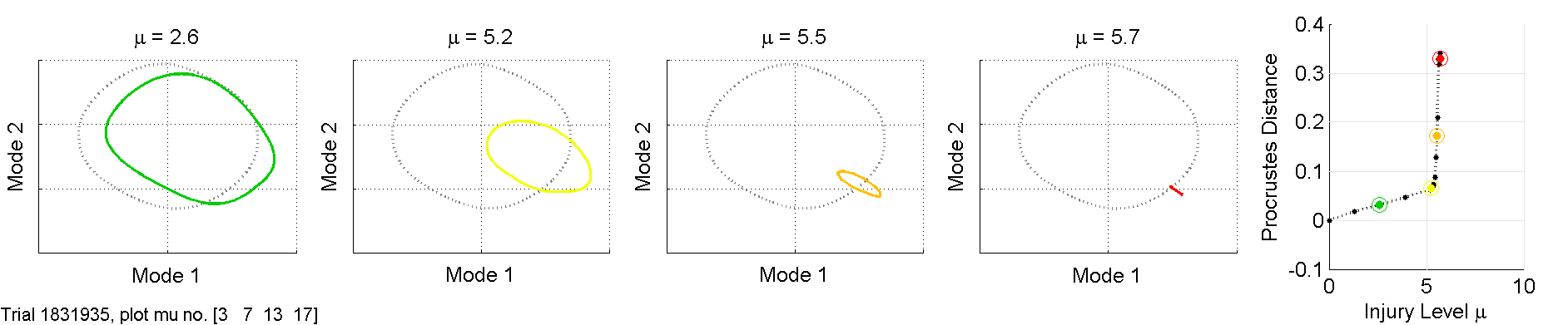

Supplement: S1 Figures — Figures similar to the rows of Fig 4, for all 1,447 trials conducted. (ZIP) [file pcbi.1005261.s002.zip › 1831935.png]

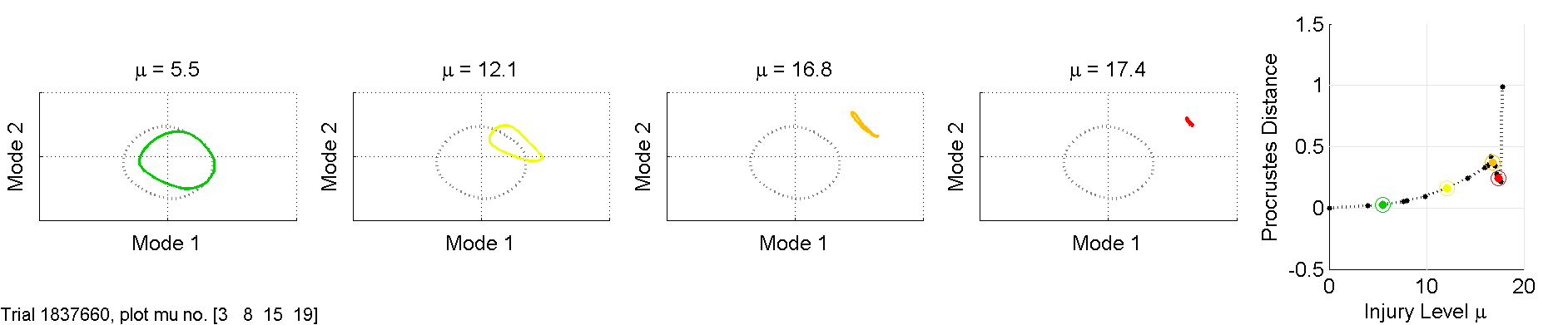

Supplement: S1 Figures — Figures similar to the rows of Fig 4, for all 1,447 trials conducted. (ZIP) [file pcbi.1005261.s002.zip › 1837660.png]

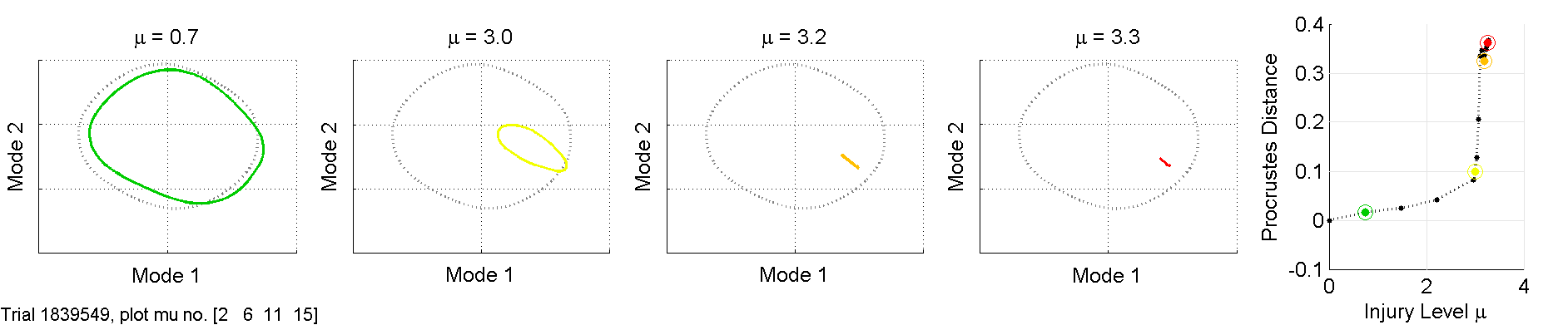

Supplement: S1 Figures — Figures similar to the rows of Fig 4, for all 1,447 trials conducted. (ZIP) [file pcbi.1005261.s002.zip › 1839549.png]

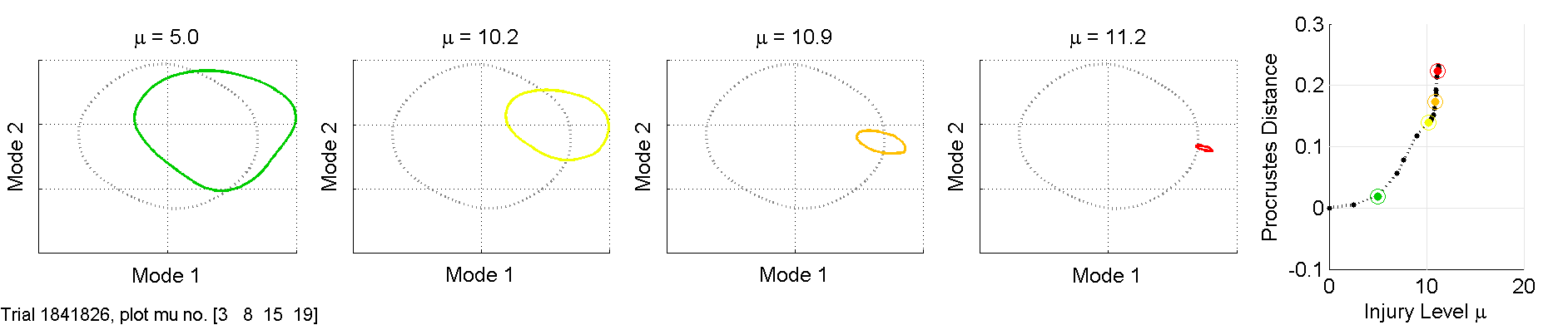

Supplement: S1 Figures — Figures similar to the rows of Fig 4, for all 1,447 trials conducted. (ZIP) [file pcbi.1005261.s002.zip › 1841826.png]

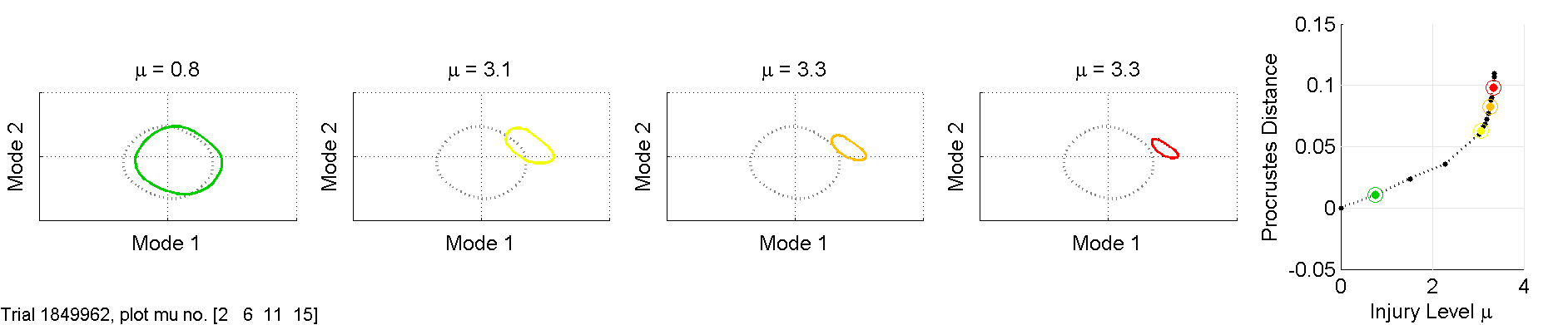

Supplement: S1 Figures — Figures similar to the rows of Fig 4, for all 1,447 trials conducted. (ZIP) [file pcbi.1005261.s002.zip › 1849962.png]

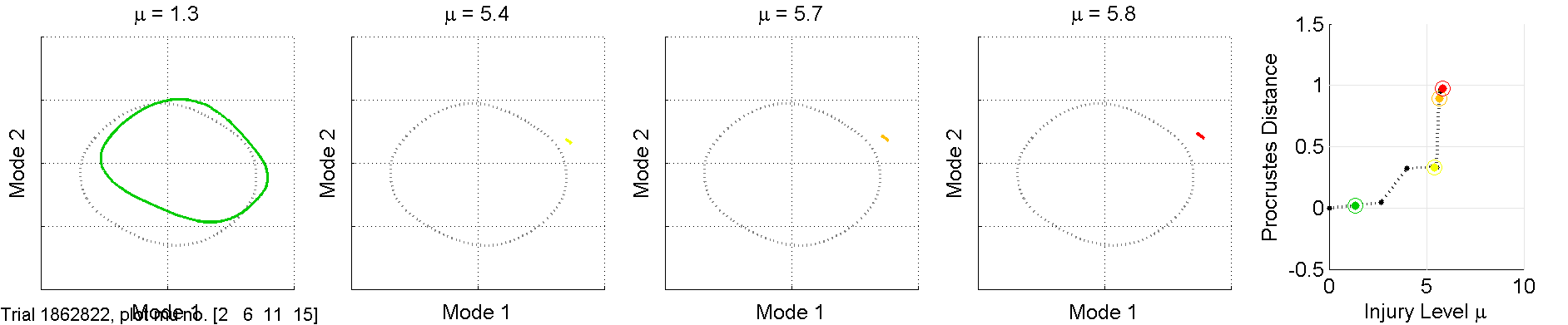

Supplement: S1 Figures — Figures similar to the rows of Fig 4, for all 1,447 trials conducted. (ZIP) [file pcbi.1005261.s002.zip › 1862822.png]

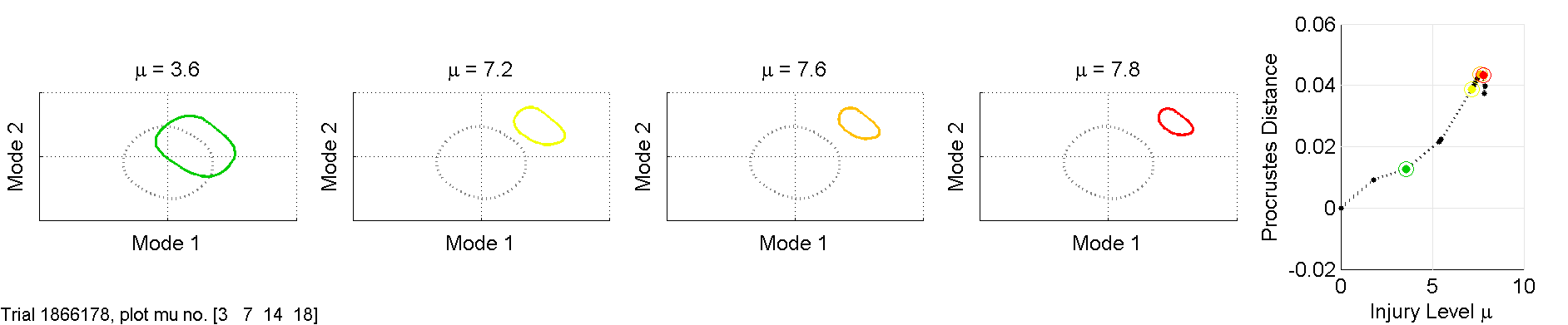

Supplement: S1 Figures — Figures similar to the rows of Fig 4, for all 1,447 trials conducted. (ZIP) [file pcbi.1005261.s002.zip › 1866178.png]

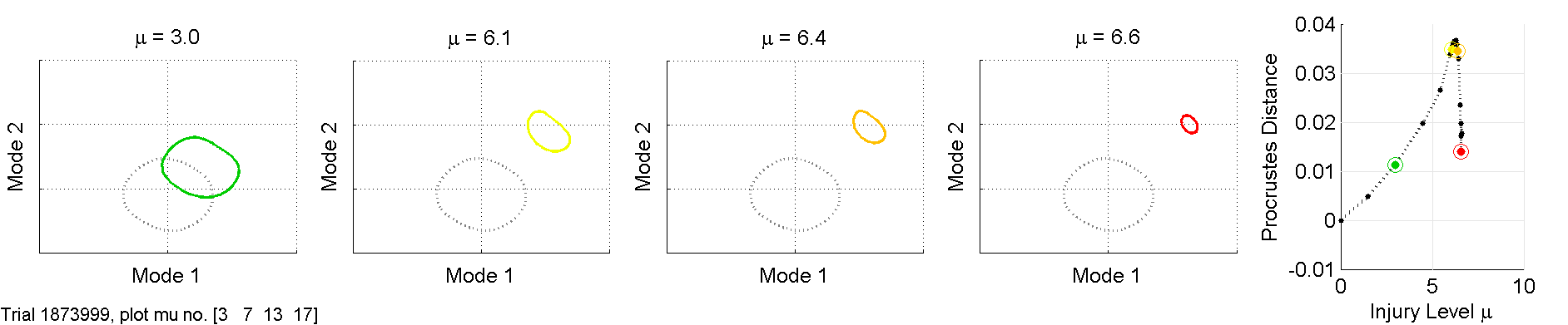

Supplement: S1 Figures — Figures similar to the rows of Fig 4, for all 1,447 trials conducted. (ZIP) [file pcbi.1005261.s002.zip › 1873999.png]

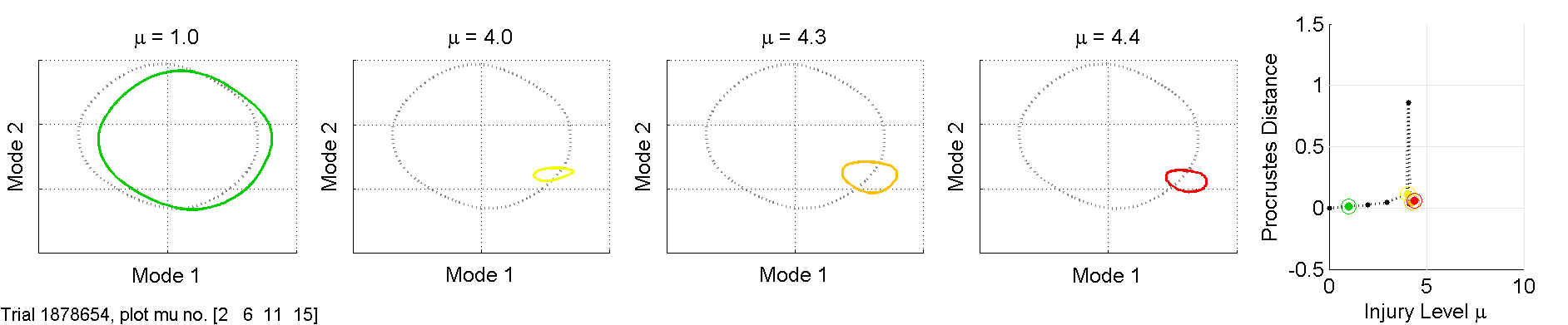

Supplement: S1 Figures — Figures similar to the rows of Fig 4, for all 1,447 trials conducted. (ZIP) [file pcbi.1005261.s002.zip › 1878654.png]

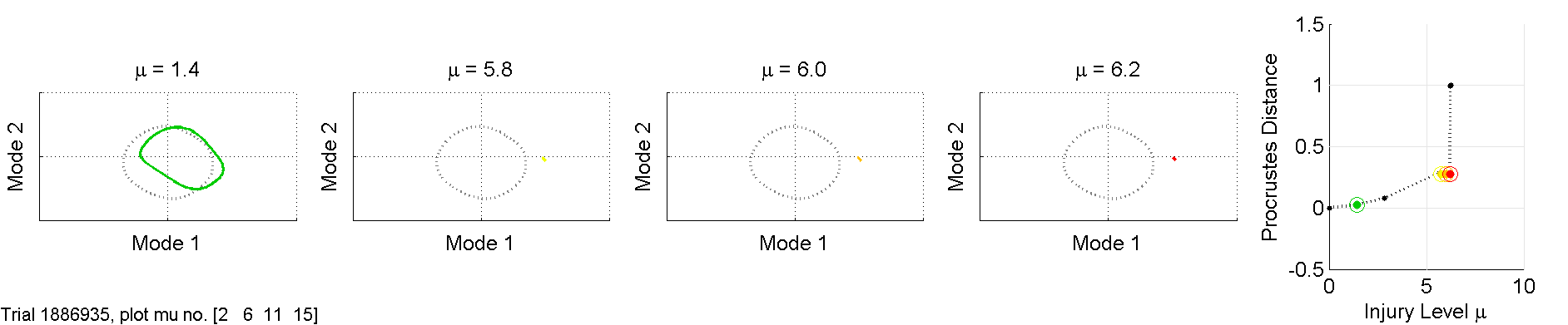

Supplement: S1 Figures — Figures similar to the rows of Fig 4, for all 1,447 trials conducted. (ZIP) [file pcbi.1005261.s002.zip › 1886935.png]

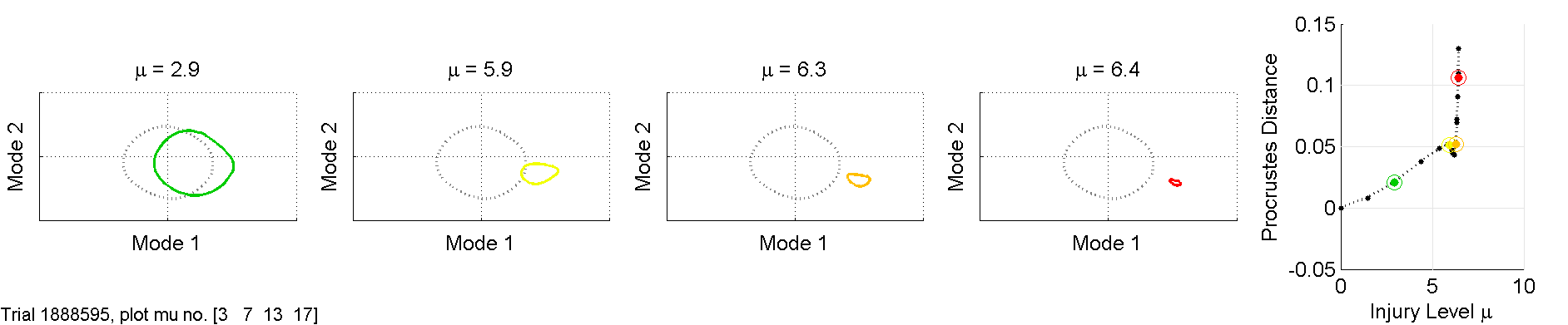

Supplement: S1 Figures — Figures similar to the rows of Fig 4, for all 1,447 trials conducted. (ZIP) [file pcbi.1005261.s002.zip › 1888595.png]

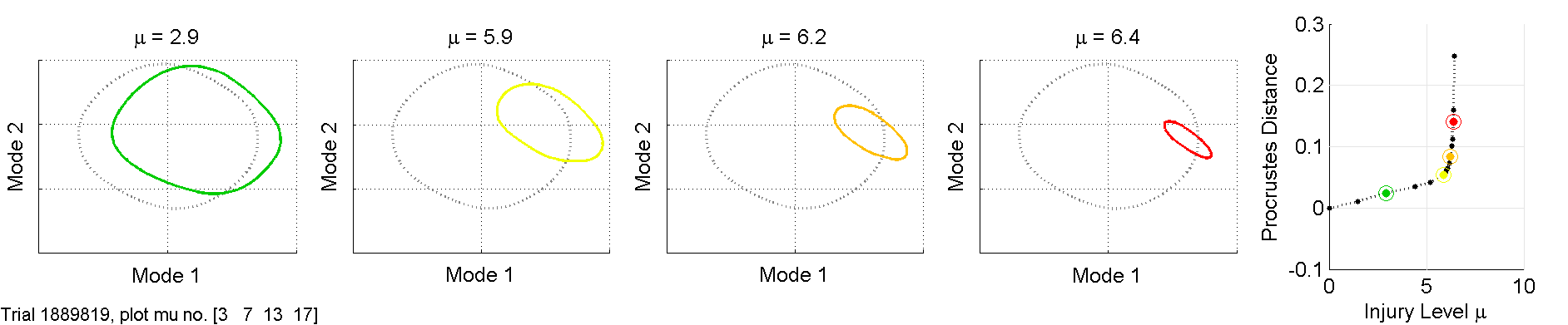

Supplement: S1 Figures — Figures similar to the rows of Fig 4, for all 1,447 trials conducted. (ZIP) [file pcbi.1005261.s002.zip › 1889819.png]

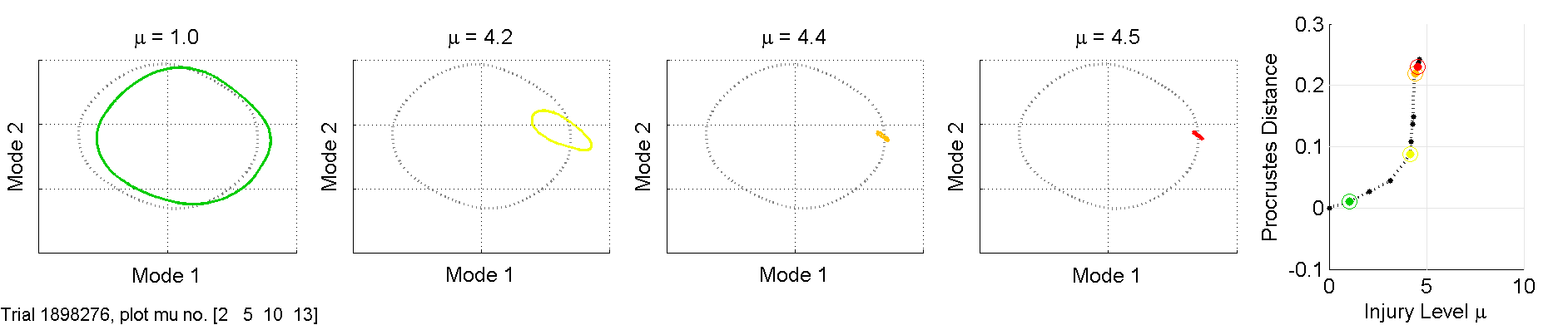

Supplement: S1 Figures — Figures similar to the rows of Fig 4, for all 1,447 trials conducted. (ZIP) [file pcbi.1005261.s002.zip › 1898276.png]

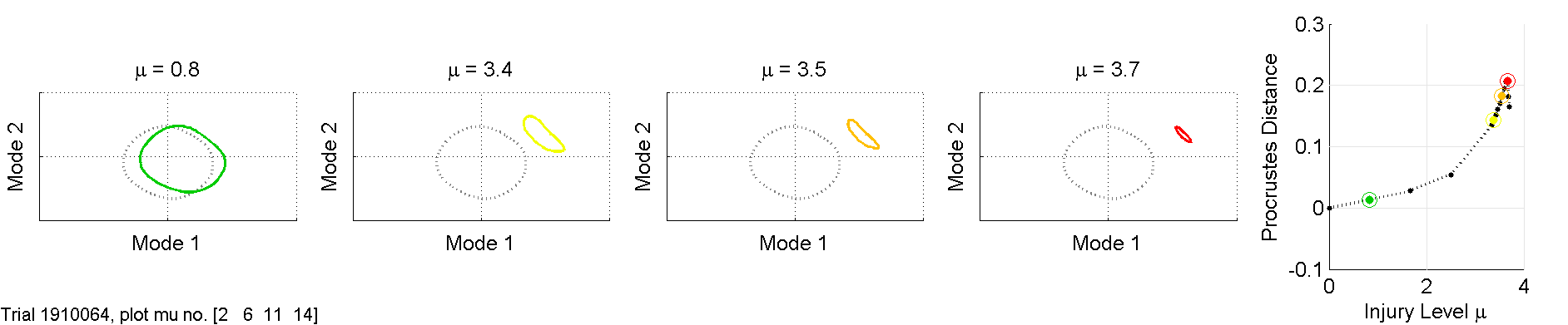

Supplement: S1 Figures — Figures similar to the rows of Fig 4, for all 1,447 trials conducted. (ZIP) [file pcbi.1005261.s002.zip › 1910064.png]

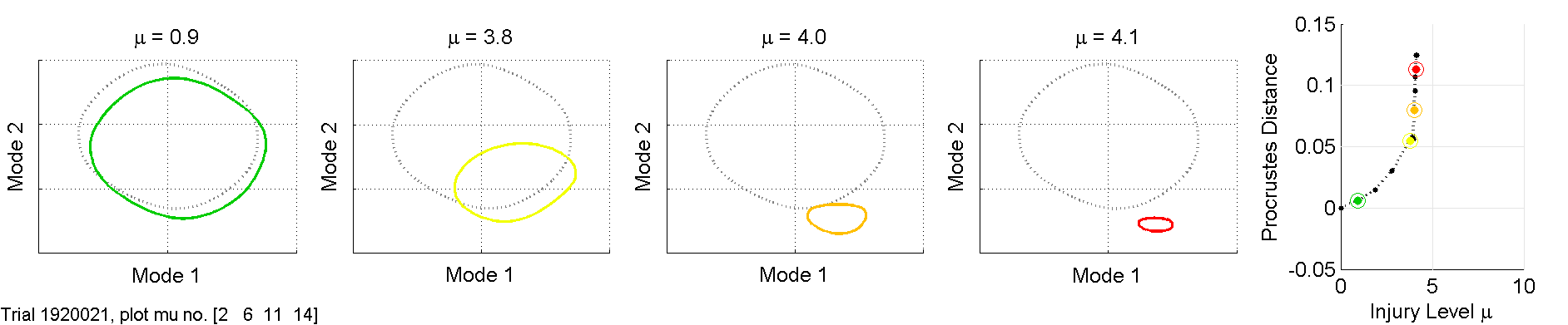

Supplement: S1 Figures — Figures similar to the rows of Fig 4, for all 1,447 trials conducted. (ZIP) [file pcbi.1005261.s002.zip › 1920021.png]

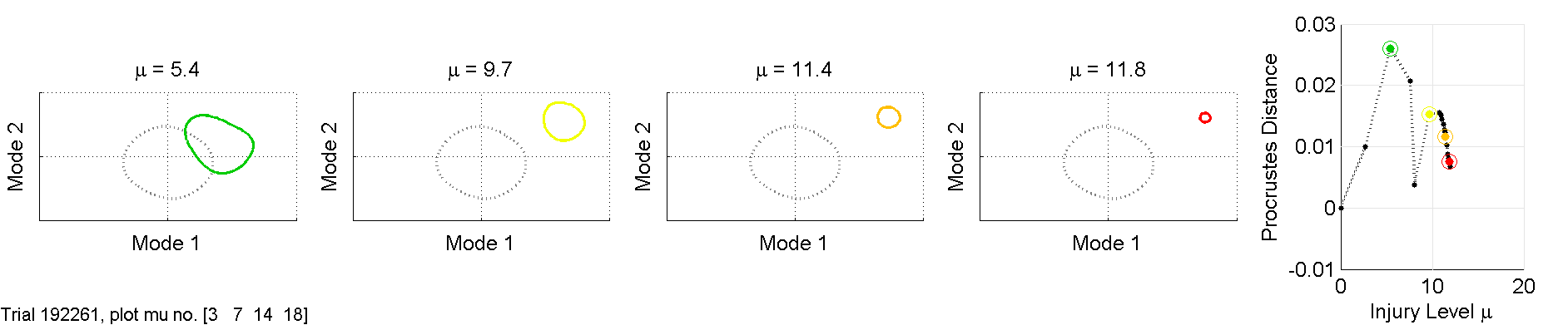

Supplement: S1 Figures — Figures similar to the rows of Fig 4, for all 1,447 trials conducted. (ZIP) [file pcbi.1005261.s002.zip › 192261.png]

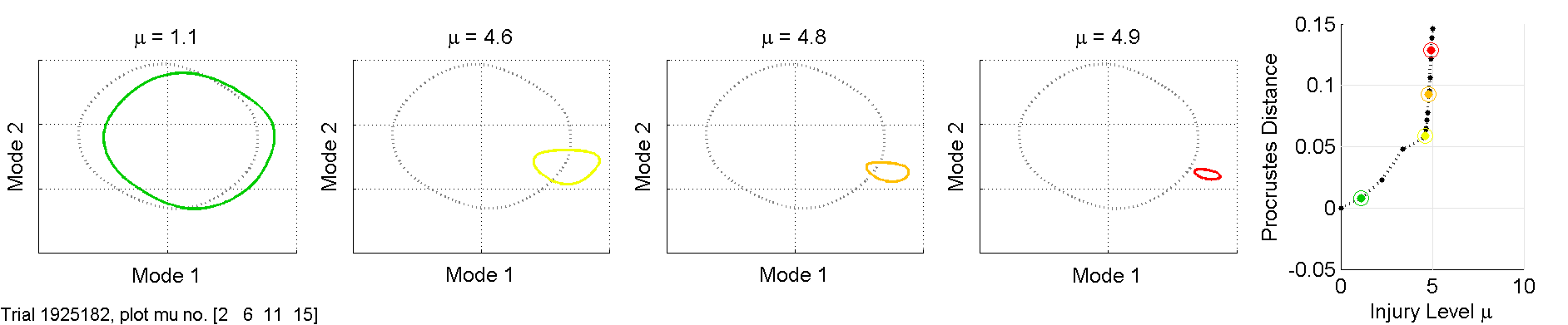

Supplement: S1 Figures — Figures similar to the rows of Fig 4, for all 1,447 trials conducted. (ZIP) [file pcbi.1005261.s002.zip › 1925182.png]

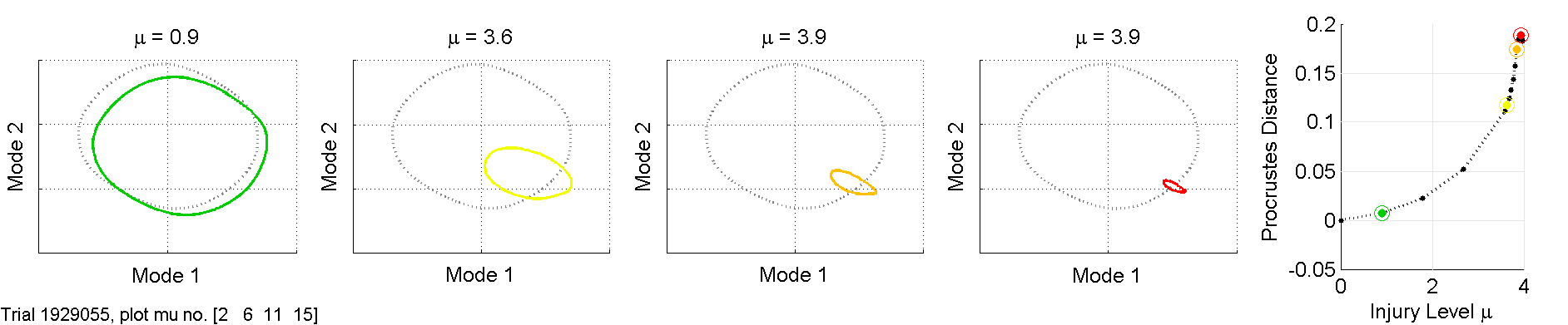

Supplement: S1 Figures — Figures similar to the rows of Fig 4, for all 1,447 trials conducted. (ZIP) [file pcbi.1005261.s002.zip › 1929055.png]

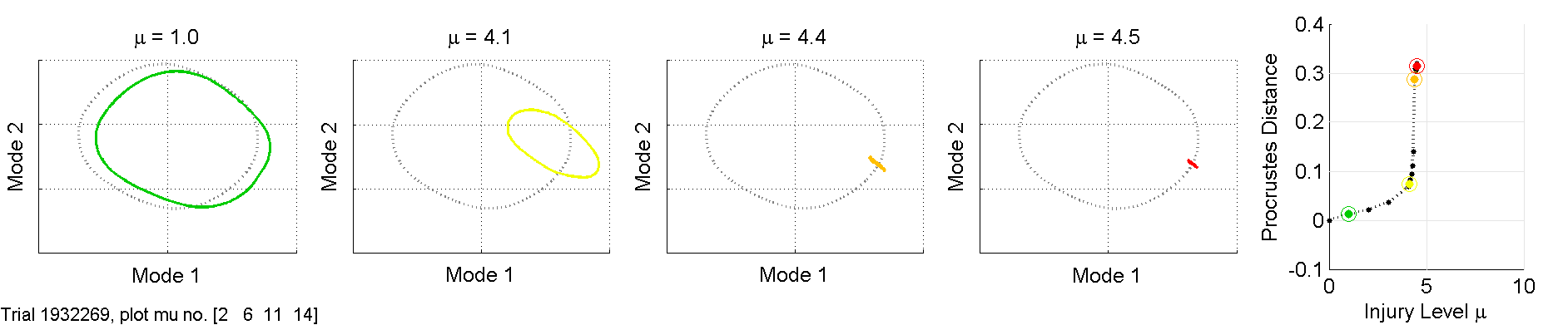

Supplement: S1 Figures — Figures similar to the rows of Fig 4, for all 1,447 trials conducted. (ZIP) [file pcbi.1005261.s002.zip › 1932269.png]

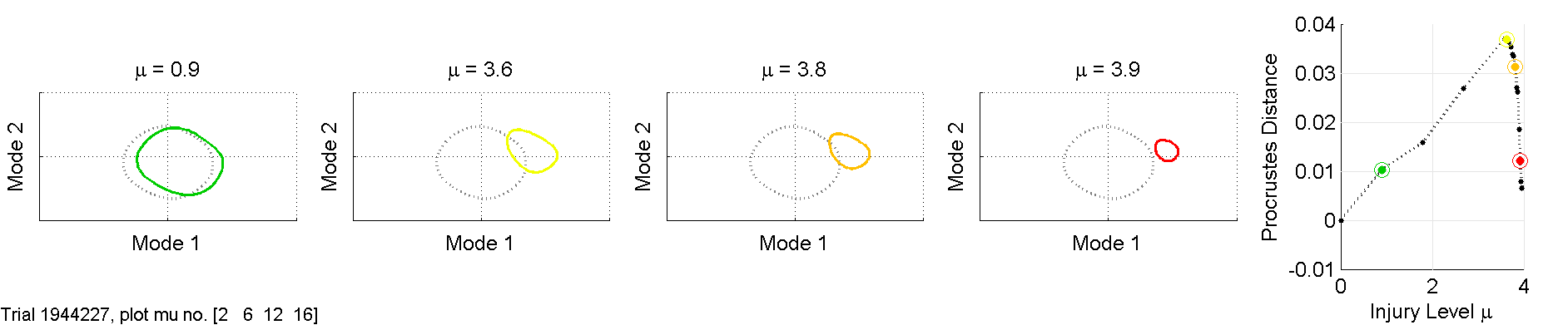

Supplement: S1 Figures — Figures similar to the rows of Fig 4, for all 1,447 trials conducted. (ZIP) [file pcbi.1005261.s002.zip › 1944227.png]

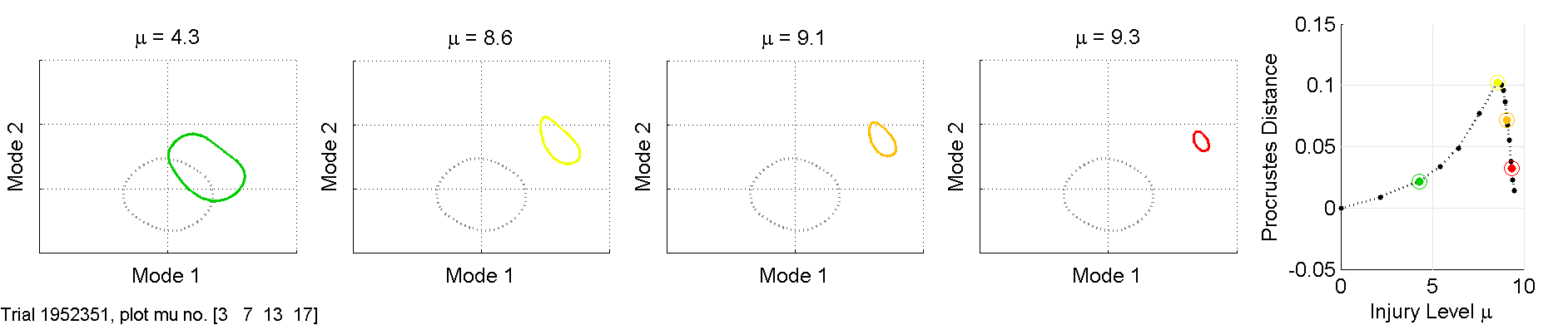

Supplement: S1 Figures — Figures similar to the rows of Fig 4, for all 1,447 trials conducted. (ZIP) [file pcbi.1005261.s002.zip › 1952351.png]

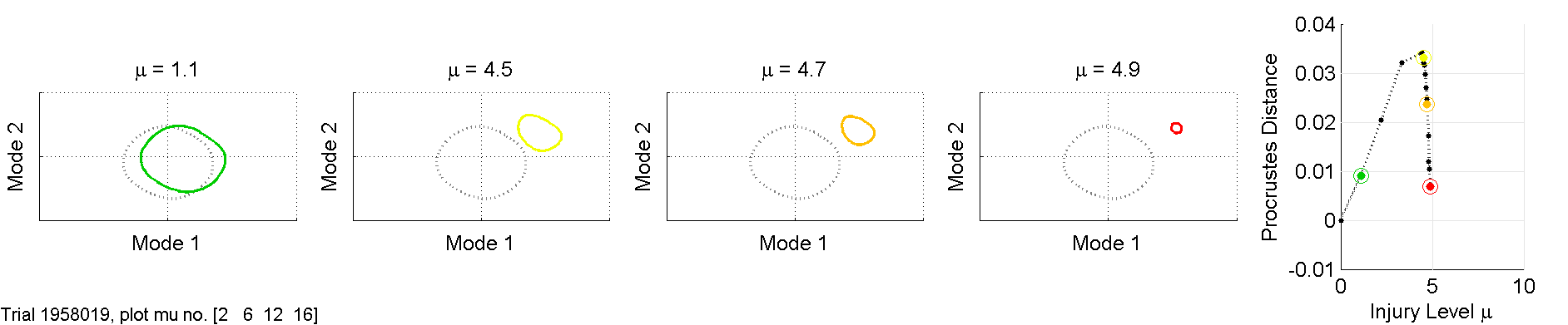

Supplement: S1 Figures — Figures similar to the rows of Fig 4, for all 1,447 trials conducted. (ZIP) [file pcbi.1005261.s002.zip › 1958019.png]

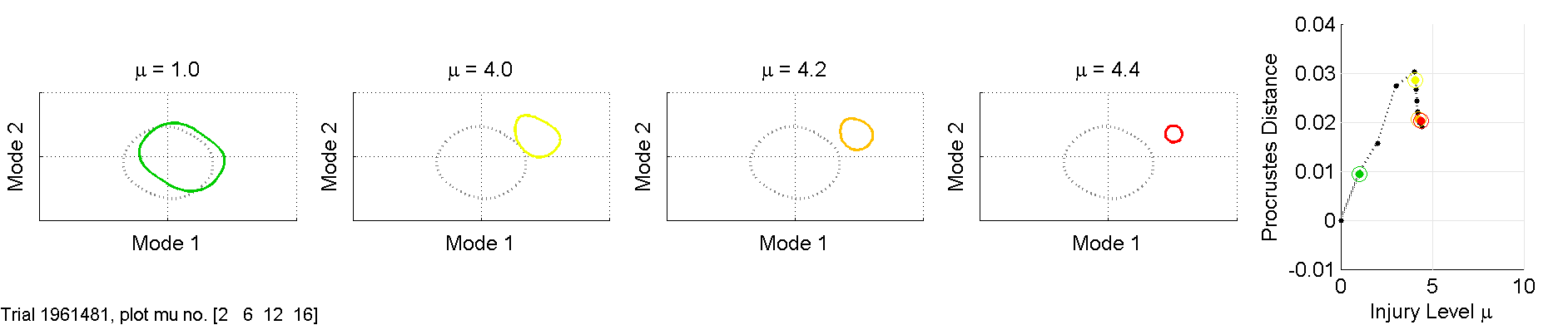

Supplement: S1 Figures — Figures similar to the rows of Fig 4, for all 1,447 trials conducted. (ZIP) [file pcbi.1005261.s002.zip › 1961481.png]

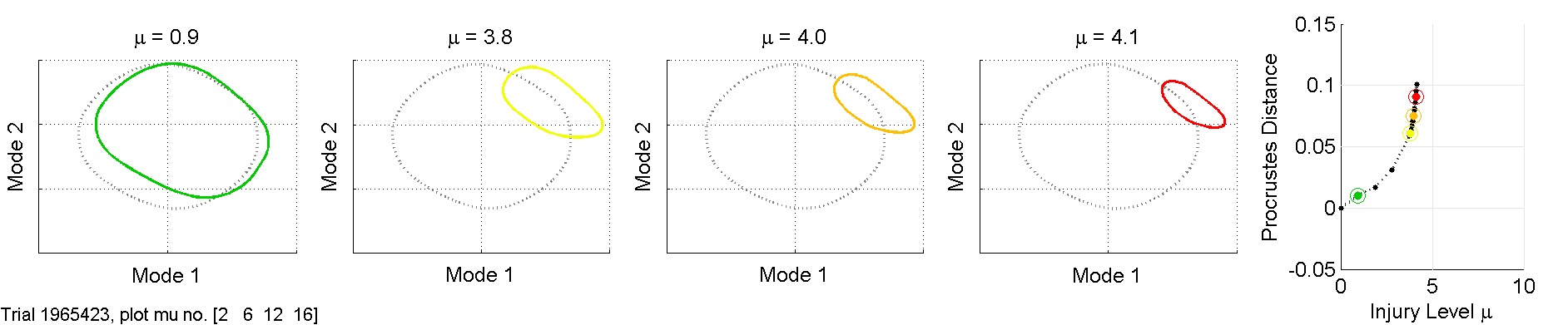

Supplement: S1 Figures — Figures similar to the rows of Fig 4, for all 1,447 trials conducted. (ZIP) [file pcbi.1005261.s002.zip › 1965423.png]

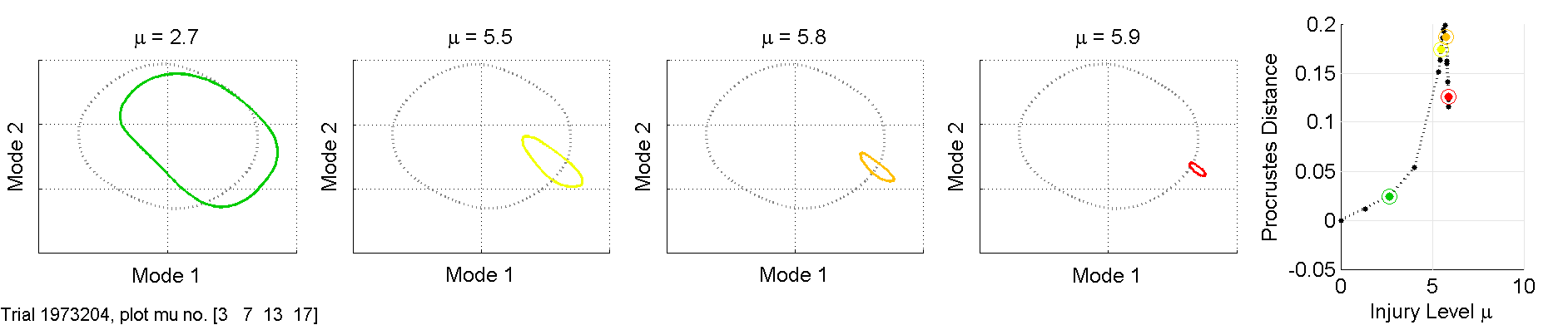

Supplement: S1 Figures — Figures similar to the rows of Fig 4, for all 1,447 trials conducted. (ZIP) [file pcbi.1005261.s002.zip › 1973204.png]

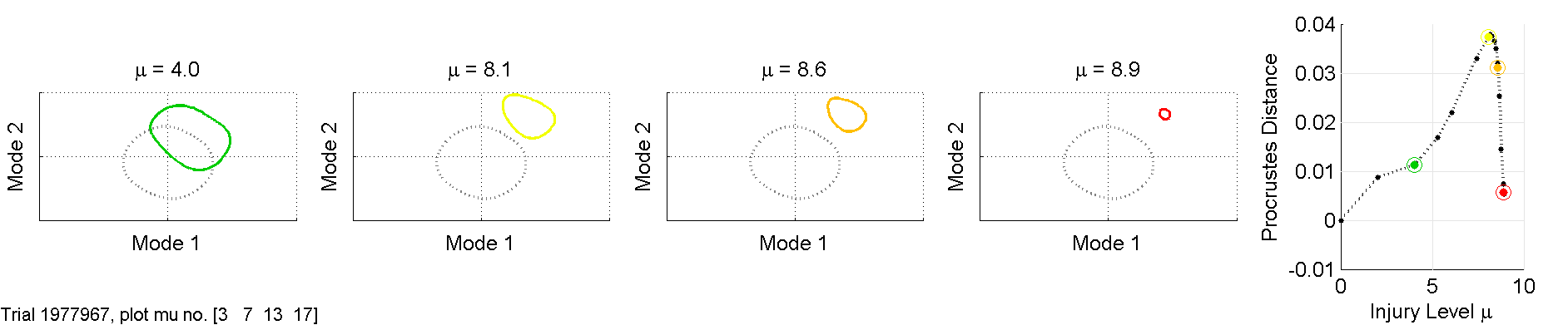

Supplement: S1 Figures — Figures similar to the rows of Fig 4, for all 1,447 trials conducted. (ZIP) [file pcbi.1005261.s002.zip › 1977967.png]

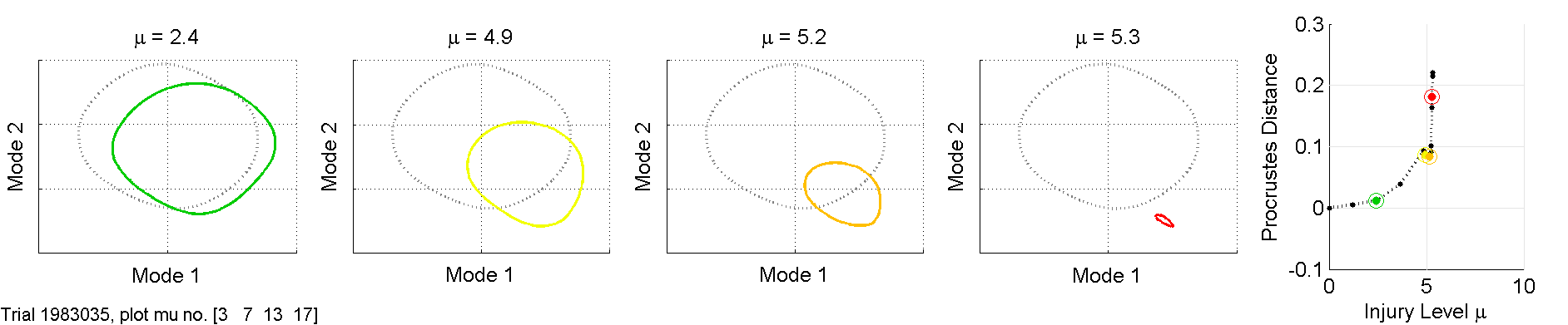

Supplement: S1 Figures — Figures similar to the rows of Fig 4, for all 1,447 trials conducted. (ZIP) [file pcbi.1005261.s002.zip › 1983035.png]

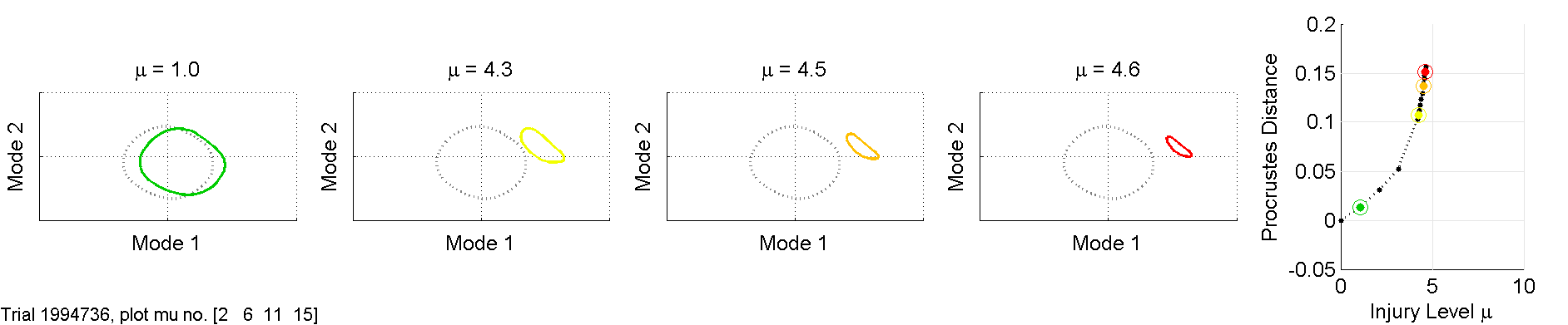

Supplement: S1 Figures — Figures similar to the rows of Fig 4, for all 1,447 trials conducted. (ZIP) [file pcbi.1005261.s002.zip › 1994736.png]

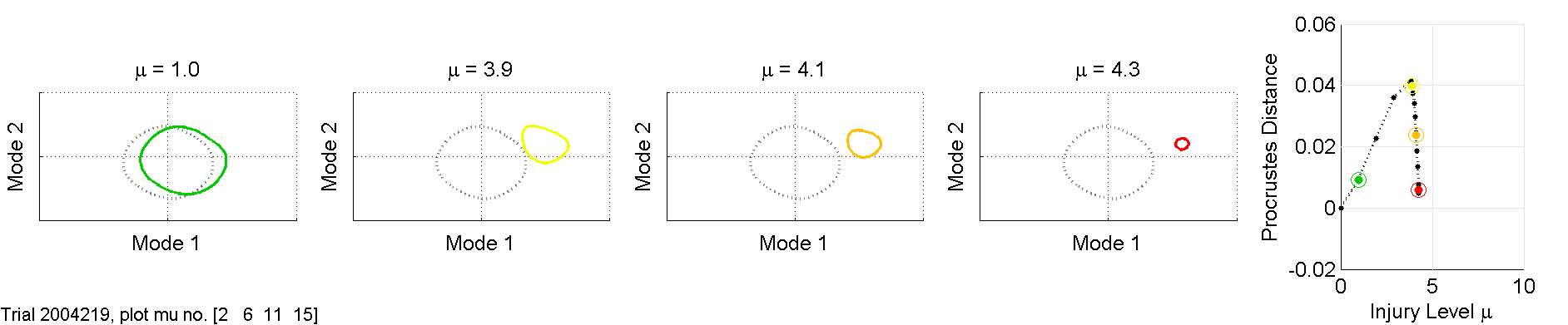

Supplement: S1 Figures — Figures similar to the rows of Fig 4, for all 1,447 trials conducted. (ZIP) [file pcbi.1005261.s002.zip › 2004219.png]

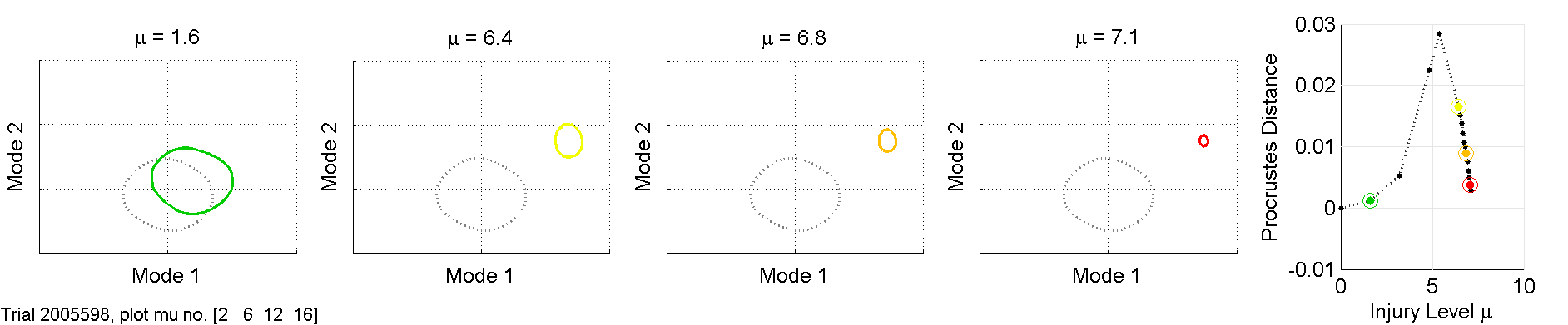

Supplement: S1 Figures — Figures similar to the rows of Fig 4, for all 1,447 trials conducted. (ZIP) [file pcbi.1005261.s002.zip › 2005598.png]

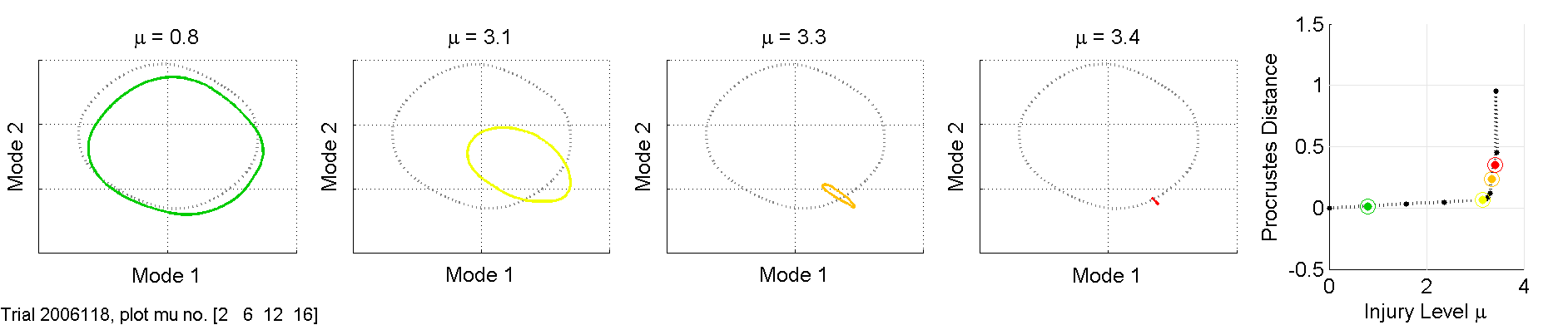

Supplement: S1 Figures — Figures similar to the rows of Fig 4, for all 1,447 trials conducted. (ZIP) [file pcbi.1005261.s002.zip › 2006118.png]

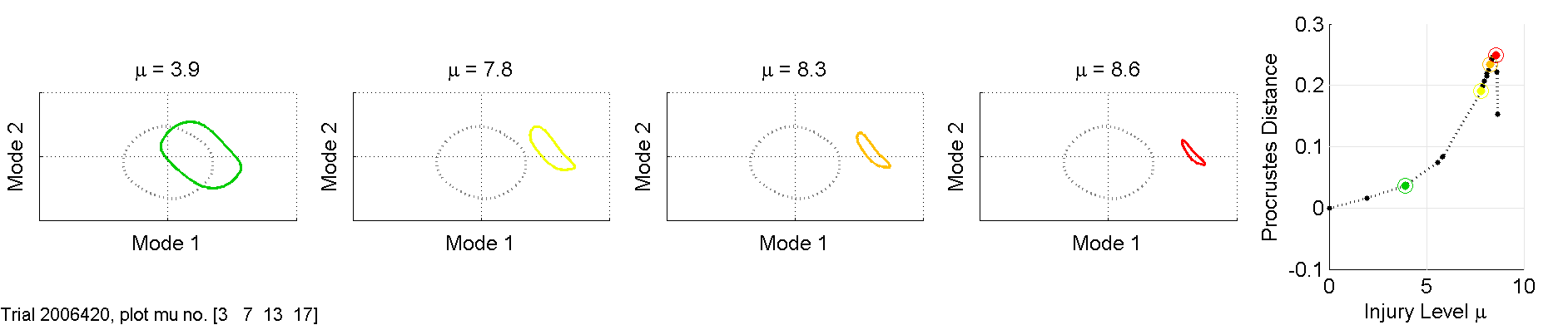

Supplement: S1 Figures — Figures similar to the rows of Fig 4, for all 1,447 trials conducted. (ZIP) [file pcbi.1005261.s002.zip › 2006420.png]

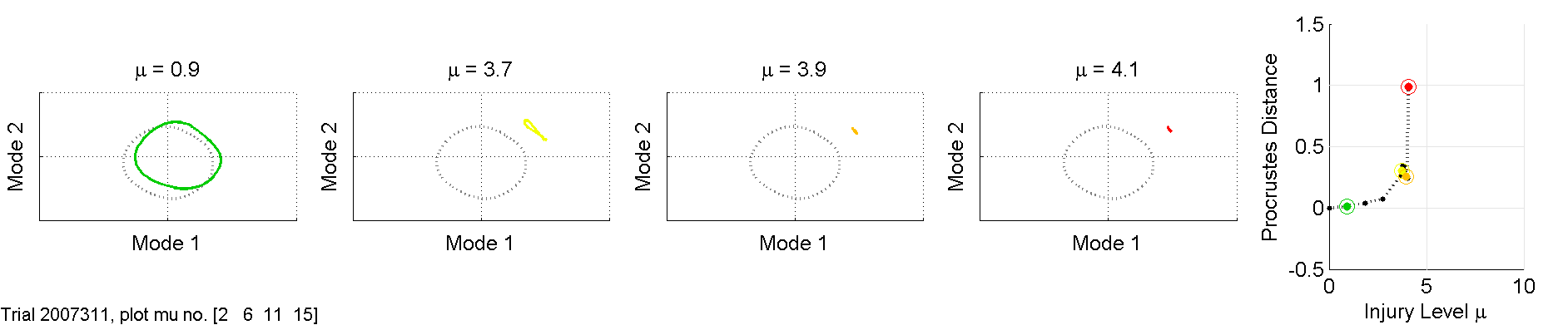

Supplement: S1 Figures — Figures similar to the rows of Fig 4, for all 1,447 trials conducted. (ZIP) [file pcbi.1005261.s002.zip › 2007311.png]

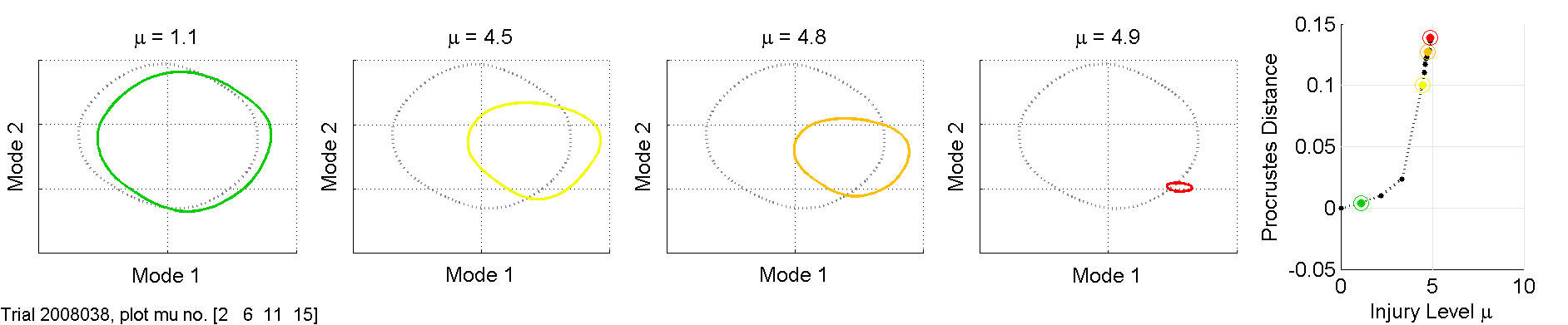

Supplement: S1 Figures — Figures similar to the rows of Fig 4, for all 1,447 trials conducted. (ZIP) [file pcbi.1005261.s002.zip › 2008038.png]

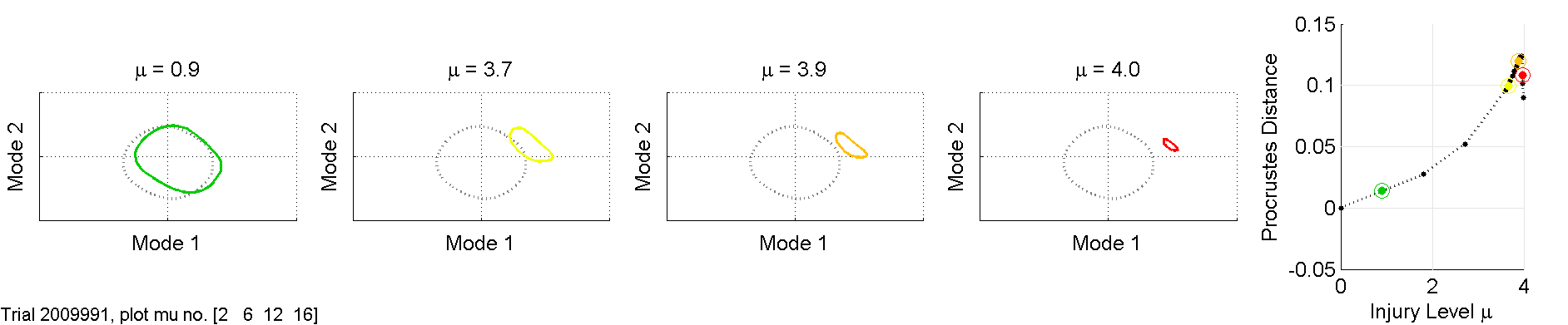

Supplement: S1 Figures — Figures similar to the rows of Fig 4, for all 1,447 trials conducted. (ZIP) [file pcbi.1005261.s002.zip › 2009991.png]

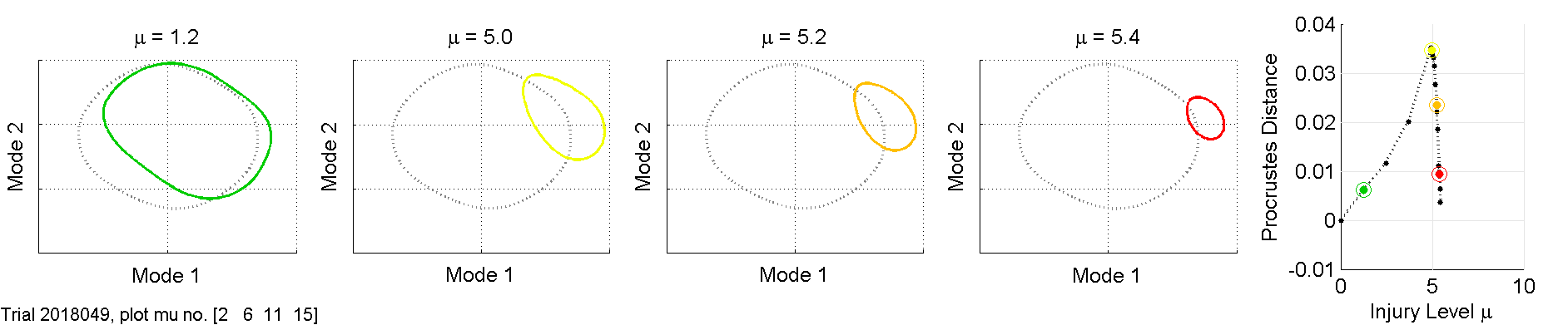

Supplement: S1 Figures — Figures similar to the rows of Fig 4, for all 1,447 trials conducted. (ZIP) [file pcbi.1005261.s002.zip › 2018049.png]

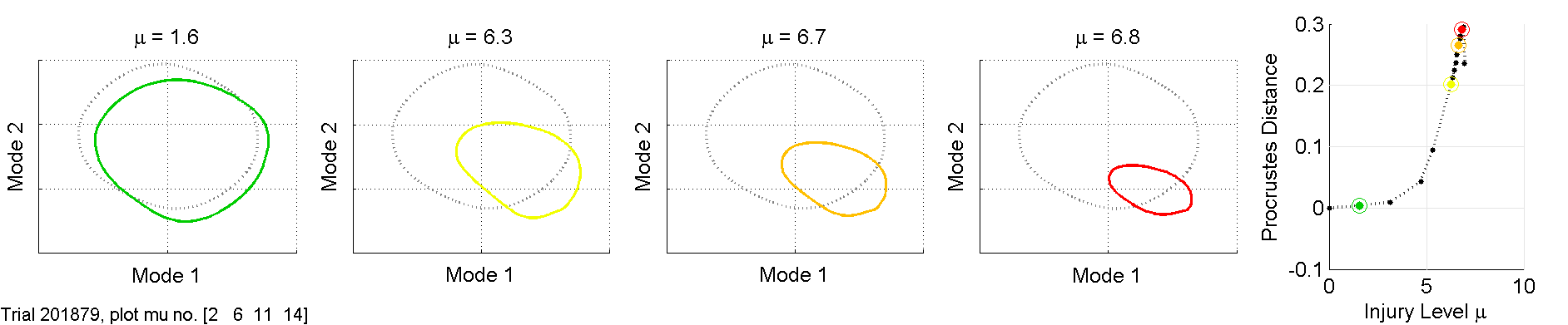

Supplement: S1 Figures — Figures similar to the rows of Fig 4, for all 1,447 trials conducted. (ZIP) [file pcbi.1005261.s002.zip › 201879.png]

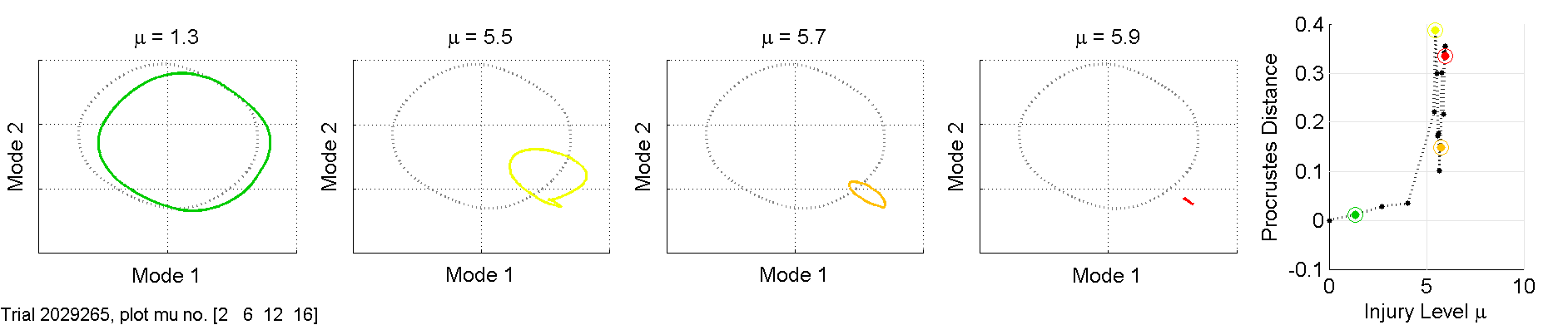

Supplement: S1 Figures — Figures similar to the rows of Fig 4, for all 1,447 trials conducted. (ZIP) [file pcbi.1005261.s002.zip › 2029265.png]

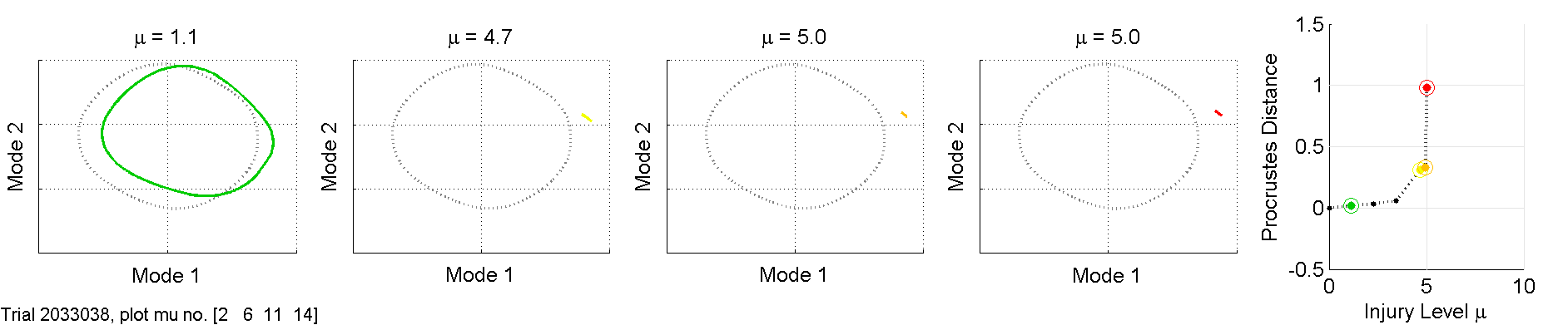

Supplement: S1 Figures — Figures similar to the rows of Fig 4, for all 1,447 trials conducted. (ZIP) [file pcbi.1005261.s002.zip › 2033038.png]

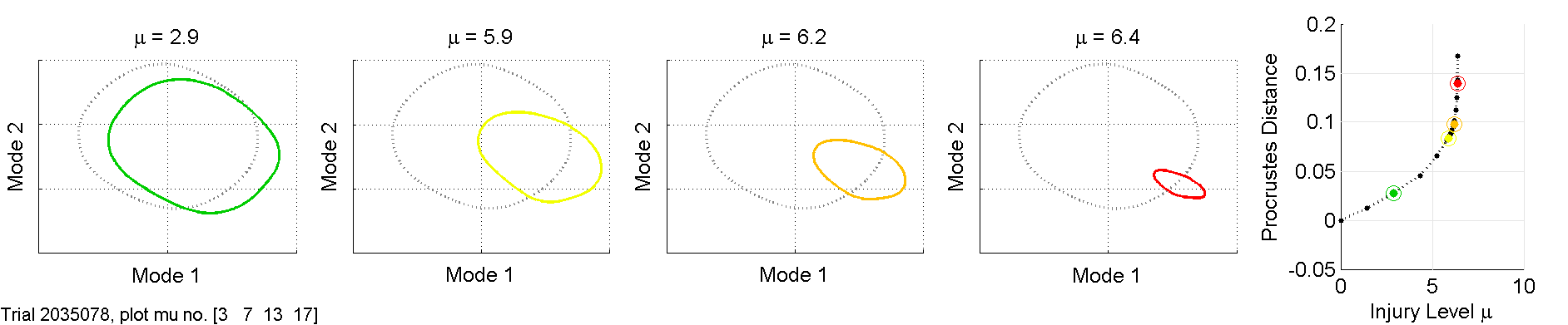

Supplement: S1 Figures — Figures similar to the rows of Fig 4, for all 1,447 trials conducted. (ZIP) [file pcbi.1005261.s002.zip › 2035078.png]

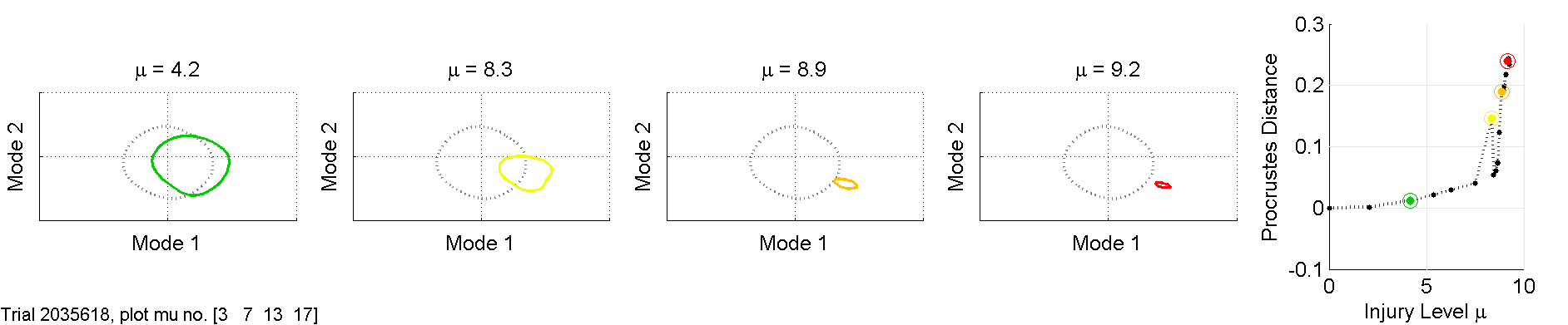

Supplement: S1 Figures — Figures similar to the rows of Fig 4, for all 1,447 trials conducted. (ZIP) [file pcbi.1005261.s002.zip › 2035618.png]

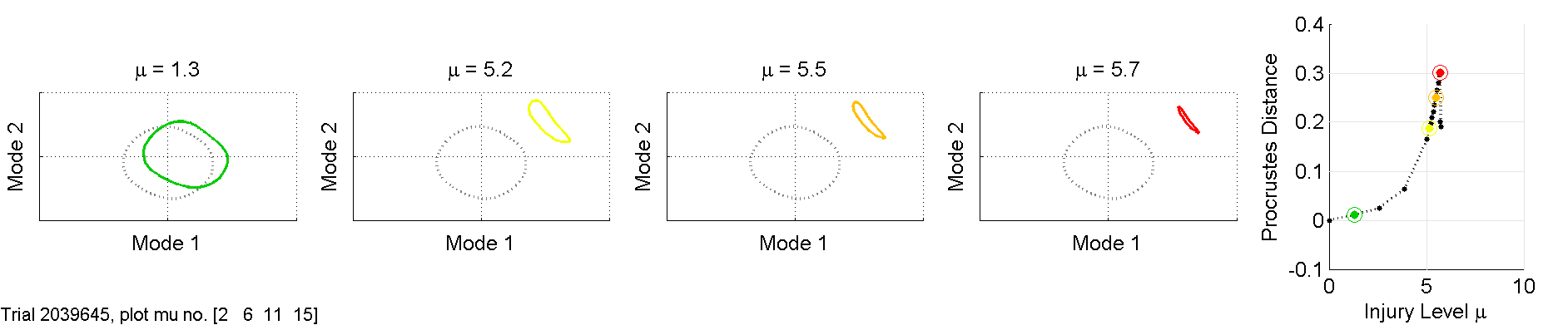

Supplement: S1 Figures — Figures similar to the rows of Fig 4, for all 1,447 trials conducted. (ZIP) [file pcbi.1005261.s002.zip › 2039645.png]

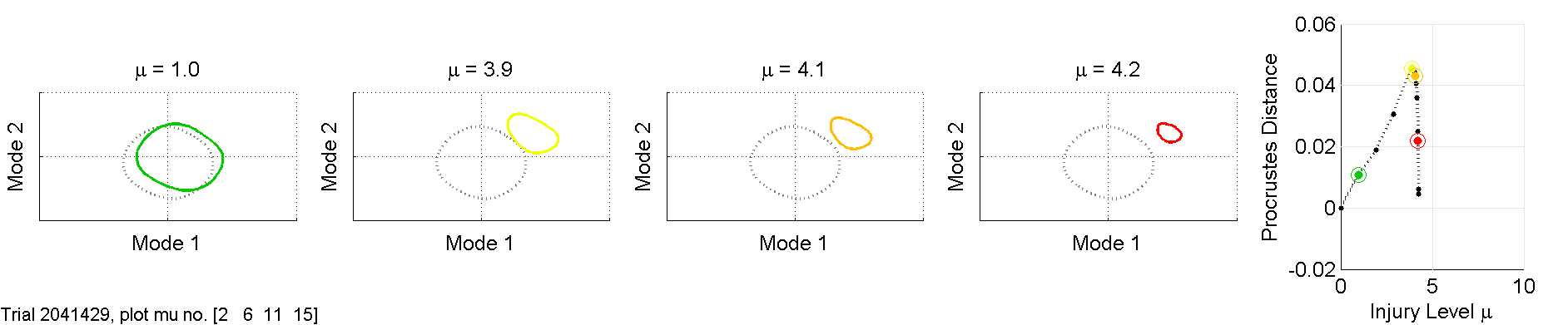

Supplement: S1 Figures — Figures similar to the rows of Fig 4, for all 1,447 trials conducted. (ZIP) [file pcbi.1005261.s002.zip › 2041429.png]

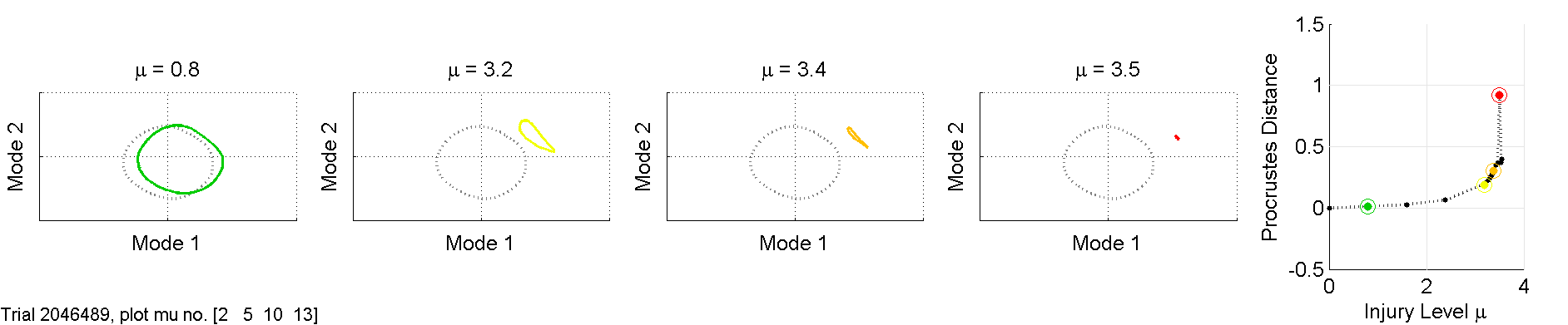

Supplement: S1 Figures — Figures similar to the rows of Fig 4, for all 1,447 trials conducted. (ZIP) [file pcbi.1005261.s002.zip › 2046489.png]

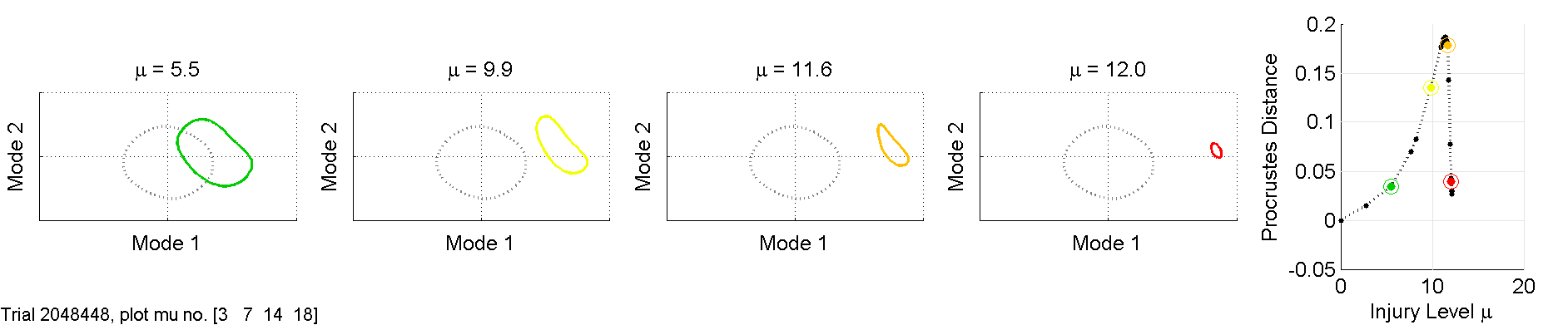

Supplement: S1 Figures — Figures similar to the rows of Fig 4, for all 1,447 trials conducted. (ZIP) [file pcbi.1005261.s002.zip › 2048448.png]

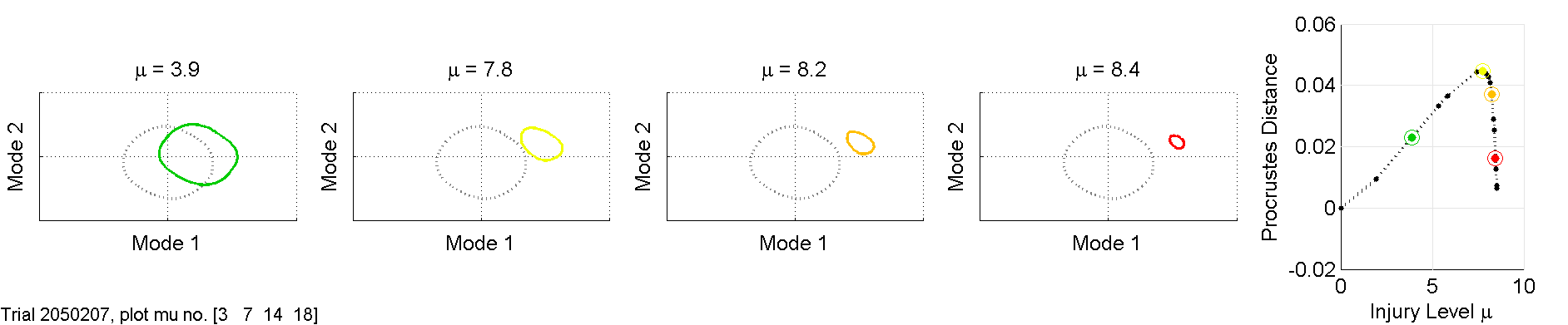

Supplement: S1 Figures — Figures similar to the rows of Fig 4, for all 1,447 trials conducted. (ZIP) [file pcbi.1005261.s002.zip › 2050207.png]

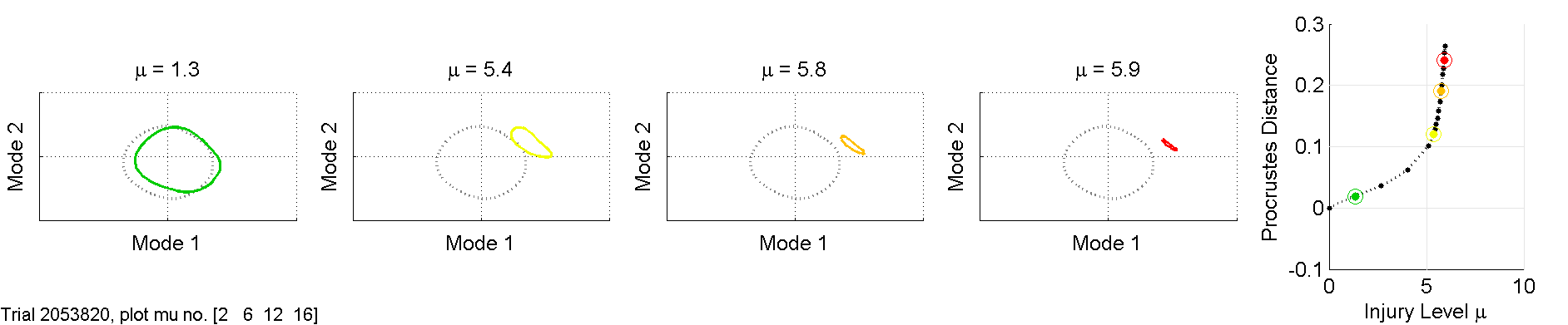

Supplement: S1 Figures — Figures similar to the rows of Fig 4, for all 1,447 trials conducted. (ZIP) [file pcbi.1005261.s002.zip › 2053820.png]

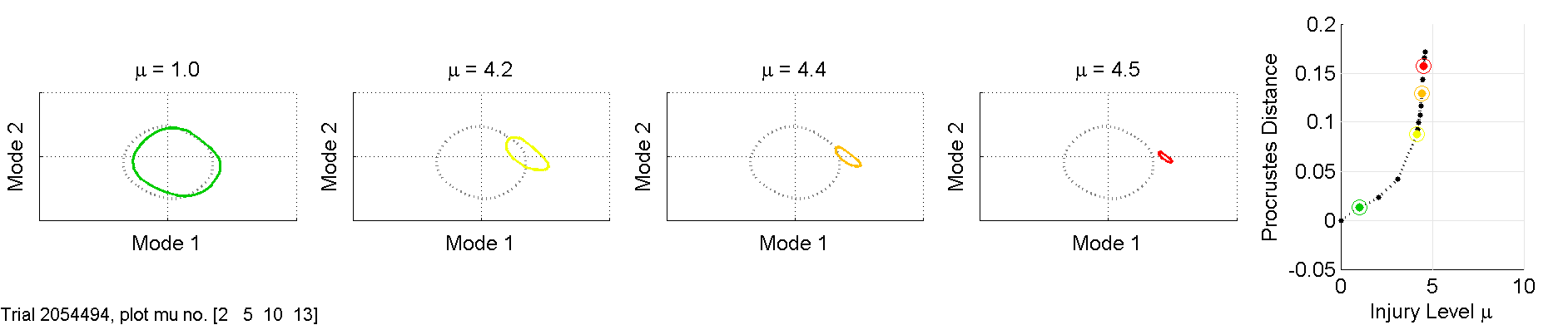

Supplement: S1 Figures — Figures similar to the rows of Fig 4, for all 1,447 trials conducted. (ZIP) [file pcbi.1005261.s002.zip › 2054494.png]

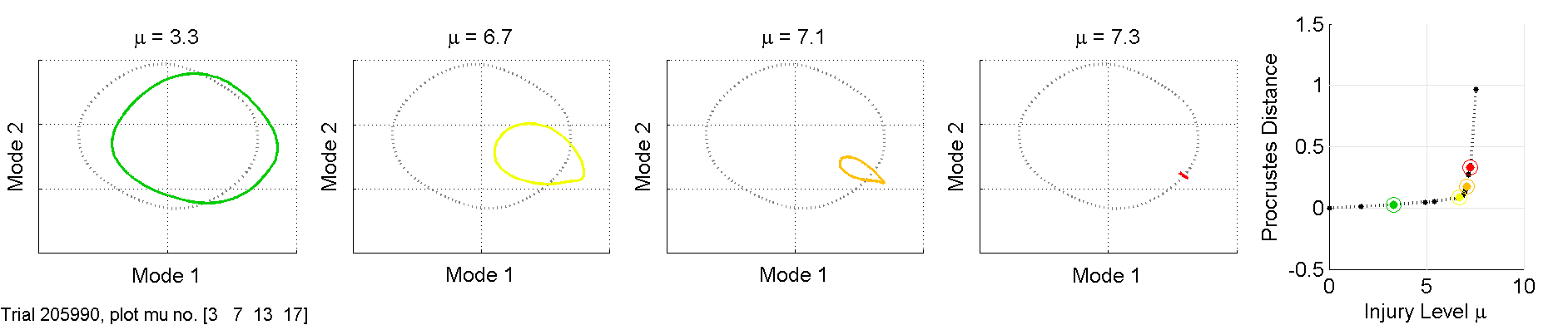

Supplement: S1 Figures — Figures similar to the rows of Fig 4, for all 1,447 trials conducted. (ZIP) [file pcbi.1005261.s002.zip › 205990.png]

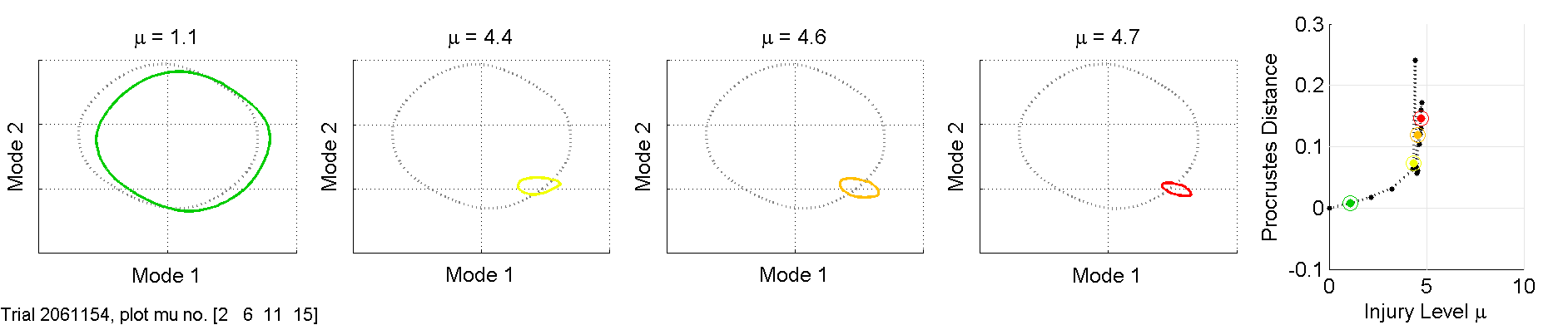

Supplement: S1 Figures — Figures similar to the rows of Fig 4, for all 1,447 trials conducted. (ZIP) [file pcbi.1005261.s002.zip › 2061154.png]

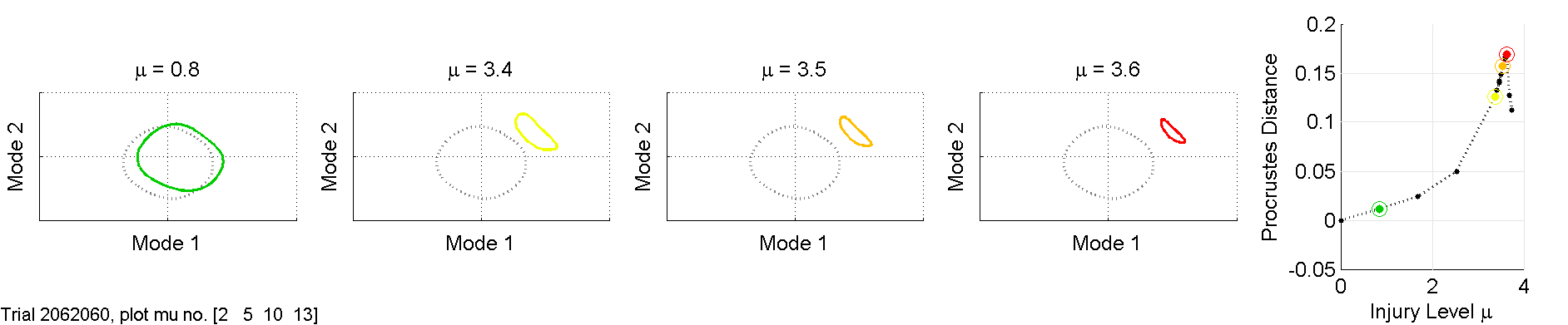

Supplement: S1 Figures — Figures similar to the rows of Fig 4, for all 1,447 trials conducted. (ZIP) [file pcbi.1005261.s002.zip › 2062060.png]

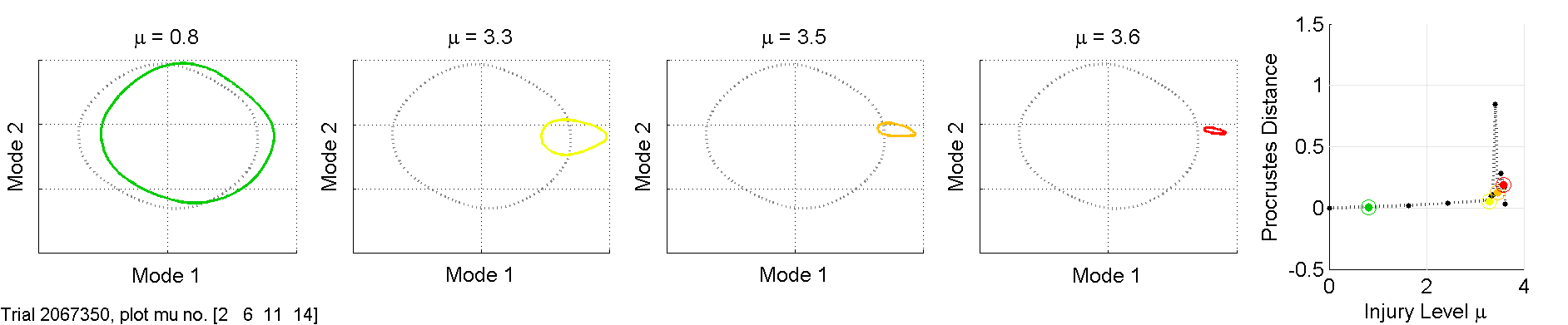

Supplement: S1 Figures — Figures similar to the rows of Fig 4, for all 1,447 trials conducted. (ZIP) [file pcbi.1005261.s002.zip › 2067350.png]

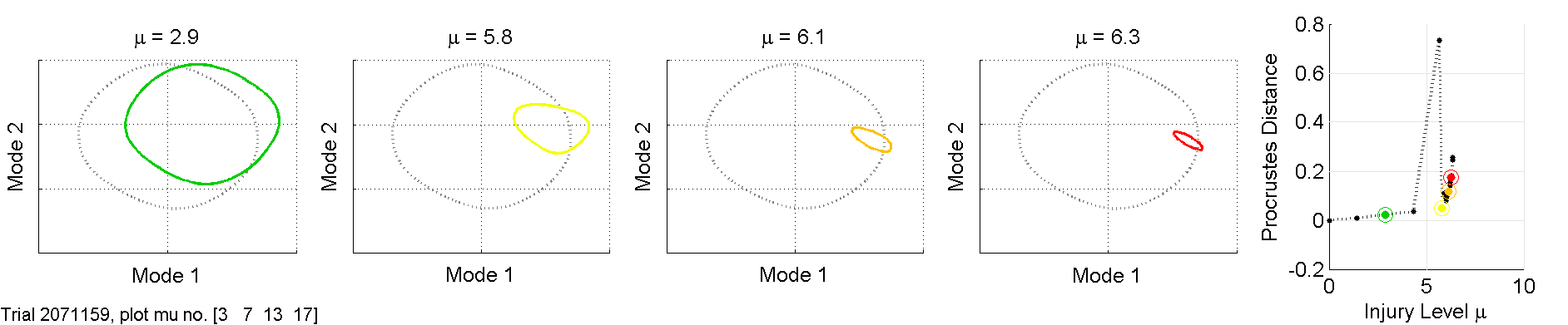

Supplement: S1 Figures — Figures similar to the rows of Fig 4, for all 1,447 trials conducted. (ZIP) [file pcbi.1005261.s002.zip › 2071159.png]

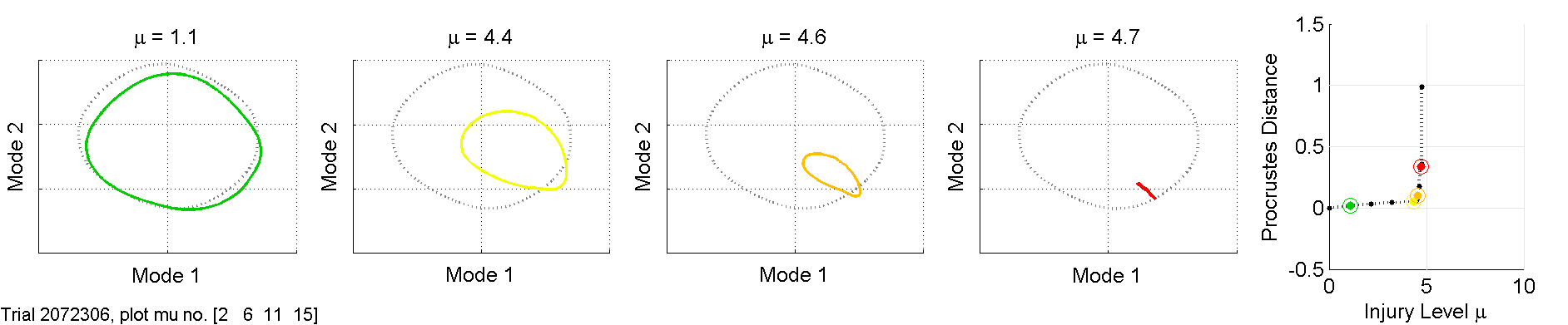

Supplement: S1 Figures — Figures similar to the rows of Fig 4, for all 1,447 trials conducted. (ZIP) [file pcbi.1005261.s002.zip › 2072306.png]

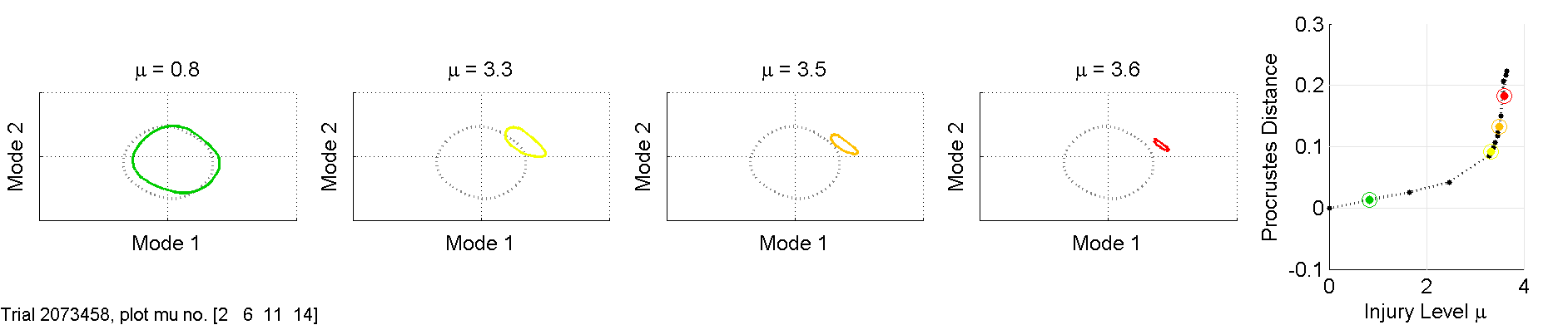

Supplement: S1 Figures — Figures similar to the rows of Fig 4, for all 1,447 trials conducted. (ZIP) [file pcbi.1005261.s002.zip › 2073458.png]

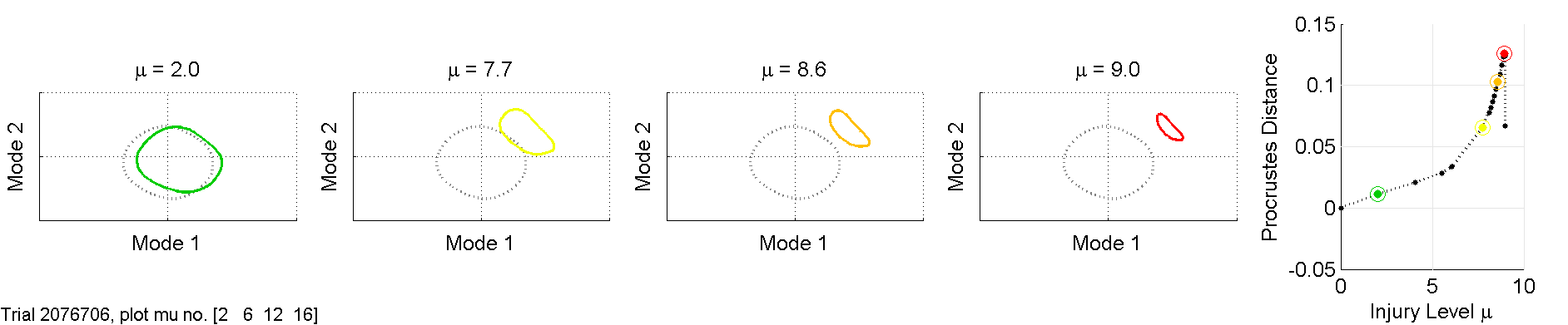

Supplement: S1 Figures — Figures similar to the rows of Fig 4, for all 1,447 trials conducted. (ZIP) [file pcbi.1005261.s002.zip › 2076706.png]

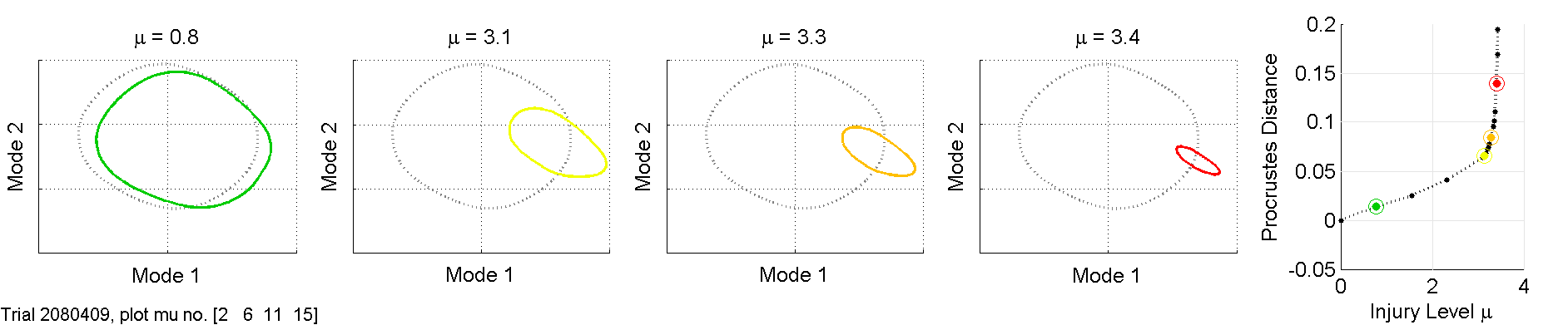

Supplement: S1 Figures — Figures similar to the rows of Fig 4, for all 1,447 trials conducted. (ZIP) [file pcbi.1005261.s002.zip › 2080409.png]

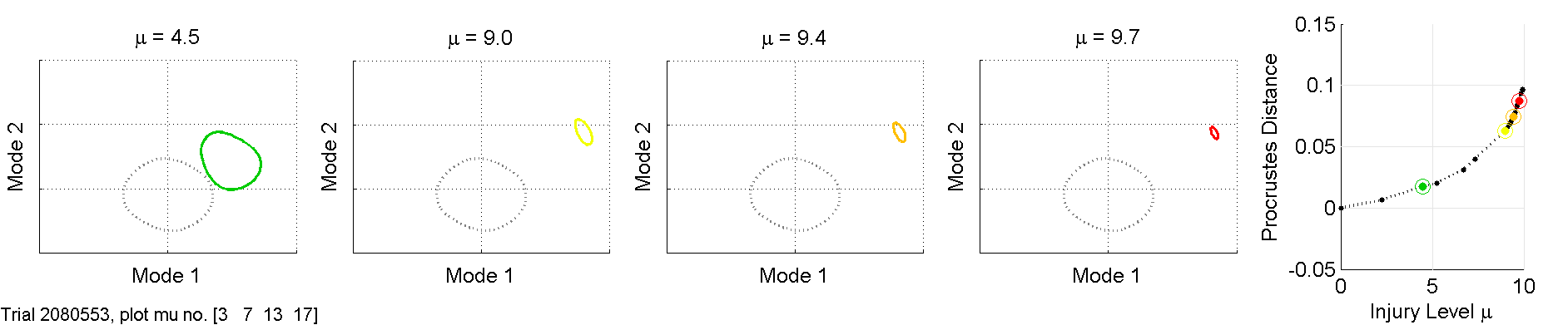

Supplement: S1 Figures — Figures similar to the rows of Fig 4, for all 1,447 trials conducted. (ZIP) [file pcbi.1005261.s002.zip › 2080553.png]

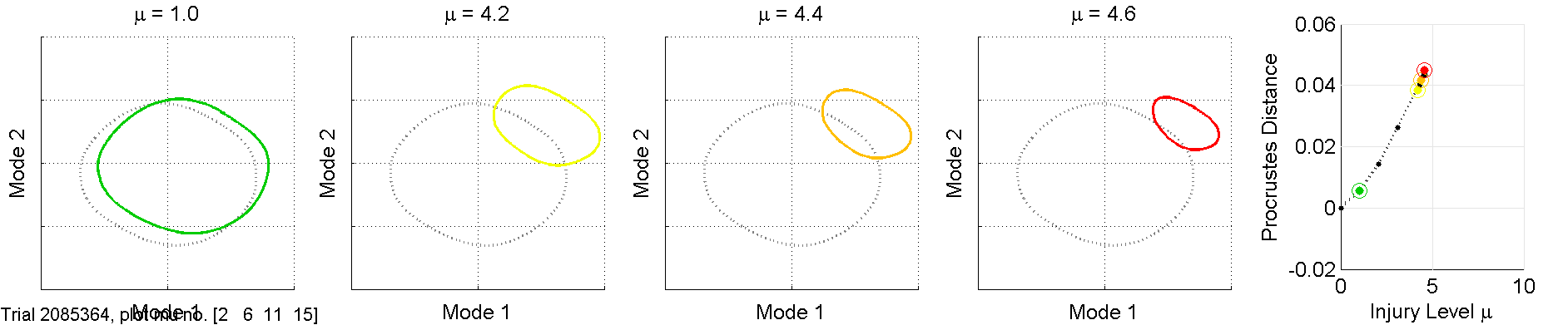

Supplement: S1 Figures — Figures similar to the rows of Fig 4, for all 1,447 trials conducted. (ZIP) [file pcbi.1005261.s002.zip › 2085364.png]

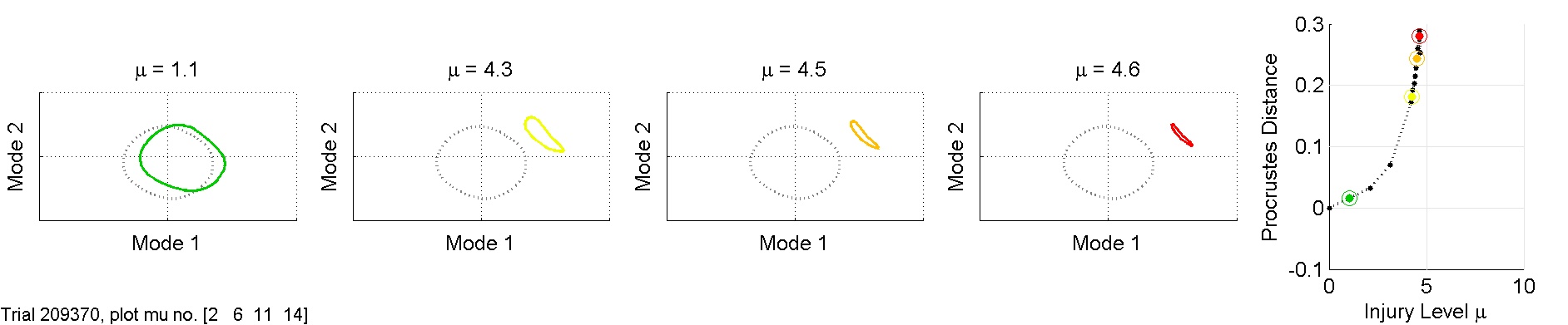

Supplement: S1 Figures — Figures similar to the rows of Fig 4, for all 1,447 trials conducted. (ZIP) [file pcbi.1005261.s002.zip › 209370.png]

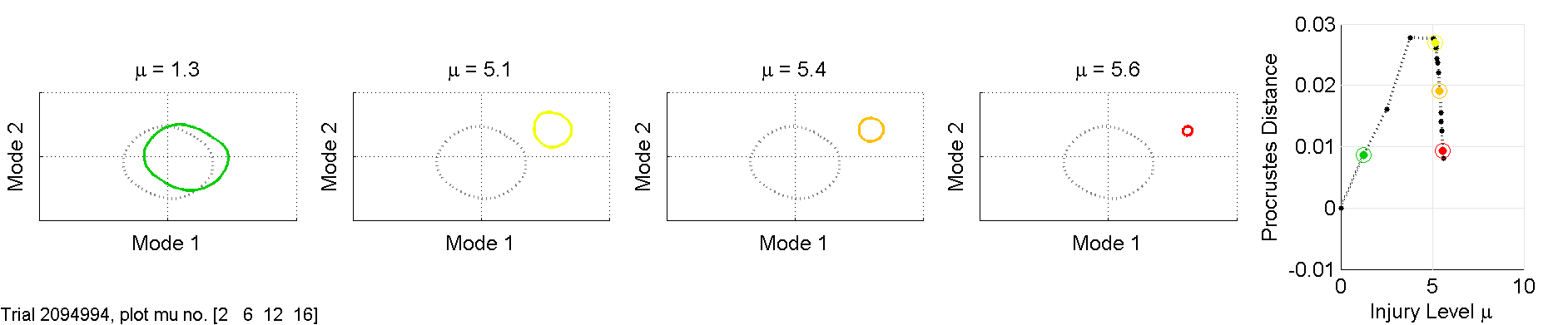

Supplement: S1 Figures — Figures similar to the rows of Fig 4, for all 1,447 trials conducted. (ZIP) [file pcbi.1005261.s002.zip › 2094994.png]

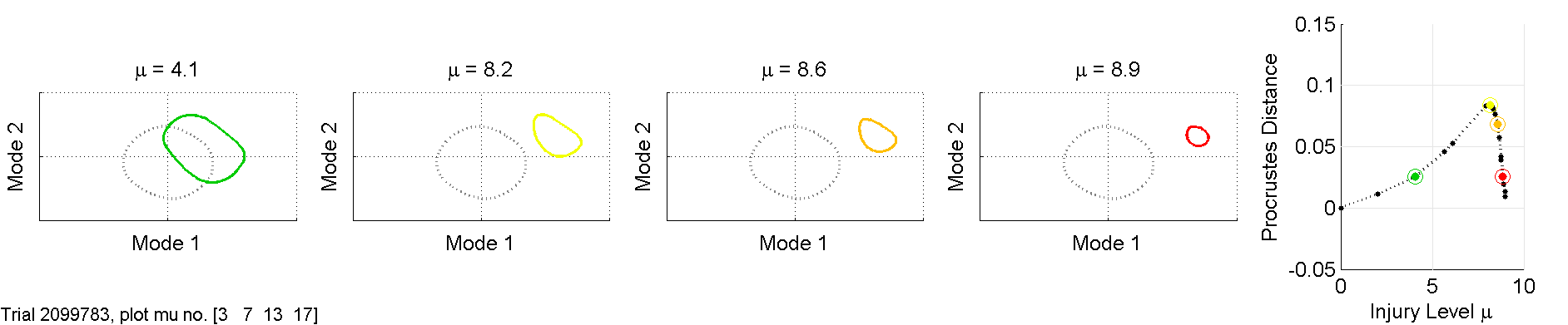

Supplement: S1 Figures — Figures similar to the rows of Fig 4, for all 1,447 trials conducted. (ZIP) [file pcbi.1005261.s002.zip › 2099783.png]

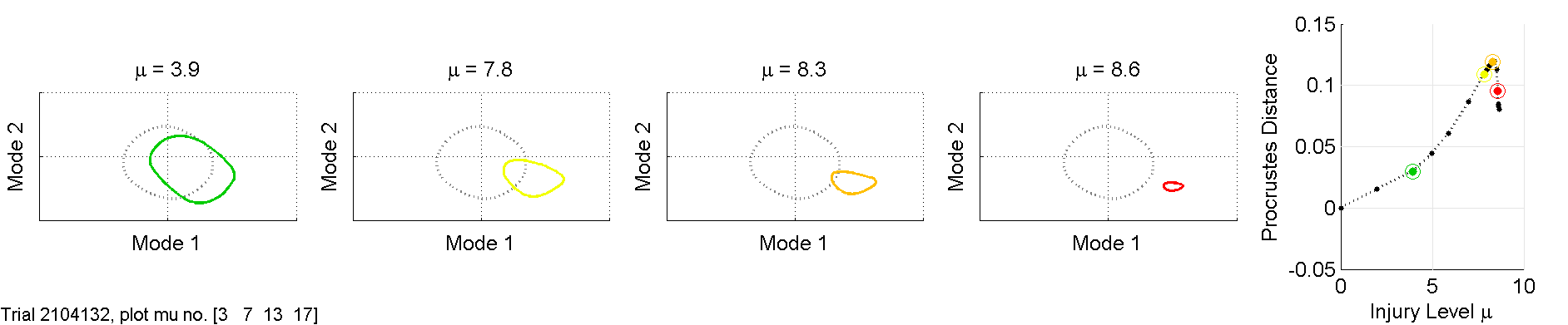

Supplement: S1 Figures — Figures similar to the rows of Fig 4, for all 1,447 trials conducted. (ZIP) [file pcbi.1005261.s002.zip › 2104132.png]

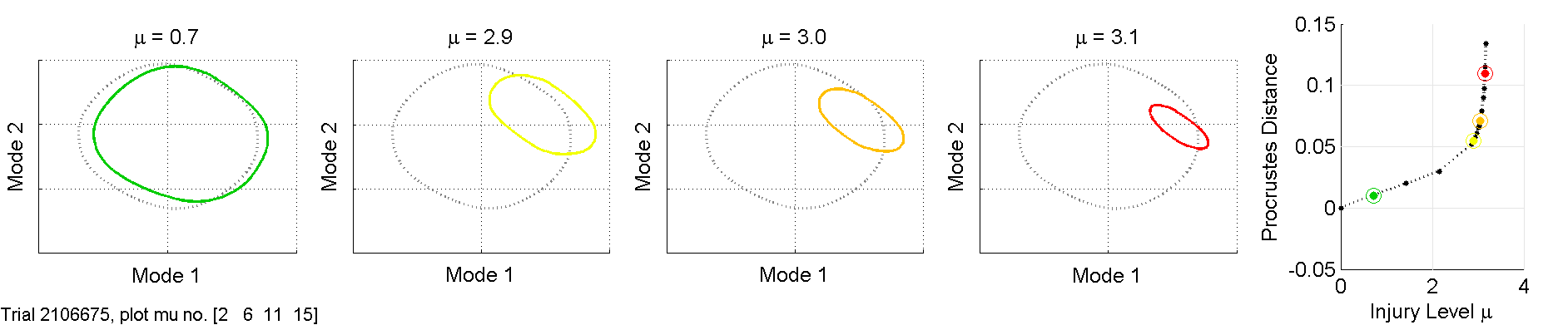

Supplement: S1 Figures — Figures similar to the rows of Fig 4, for all 1,447 trials conducted. (ZIP) [file pcbi.1005261.s002.zip › 2106675.png]

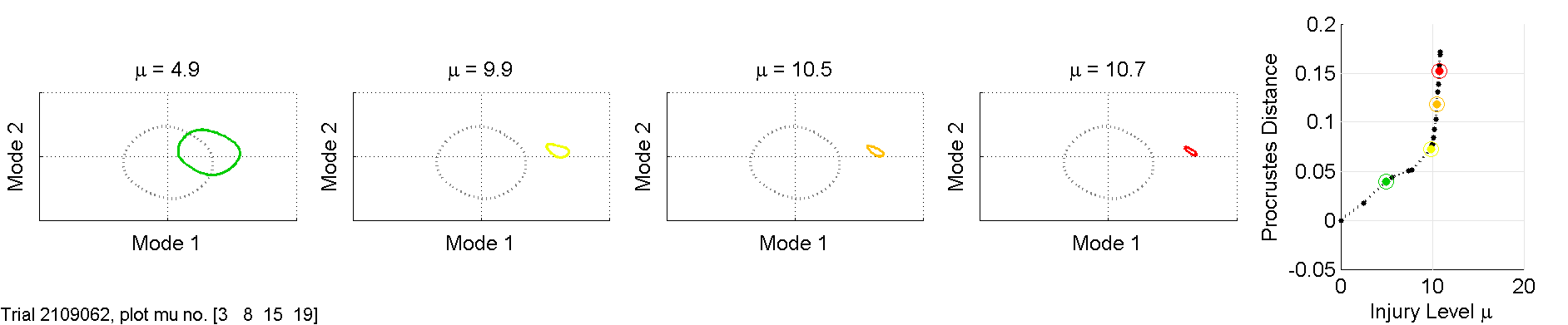

Supplement: S1 Figures — Figures similar to the rows of Fig 4, for all 1,447 trials conducted. (ZIP) [file pcbi.1005261.s002.zip › 2109062.png]

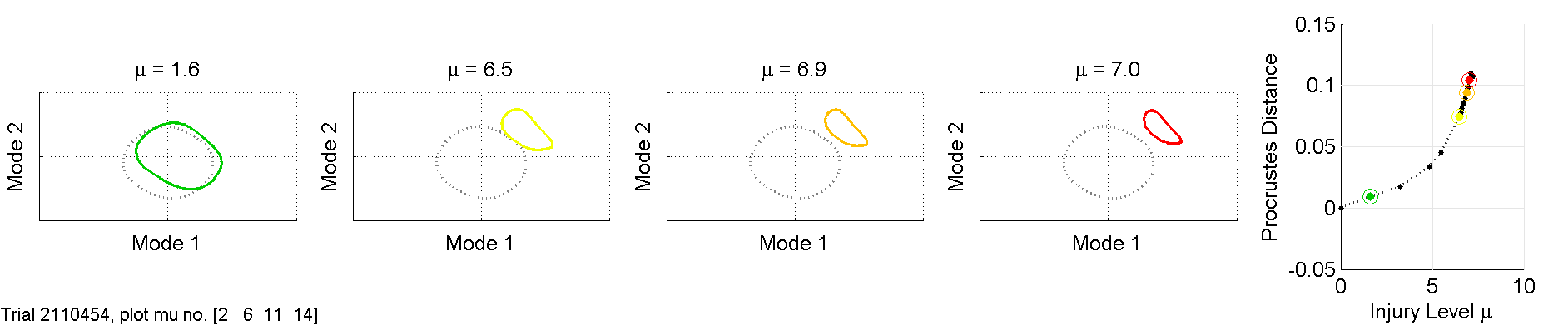

Supplement: S1 Figures — Figures similar to the rows of Fig 4, for all 1,447 trials conducted. (ZIP) [file pcbi.1005261.s002.zip › 2110454.png]

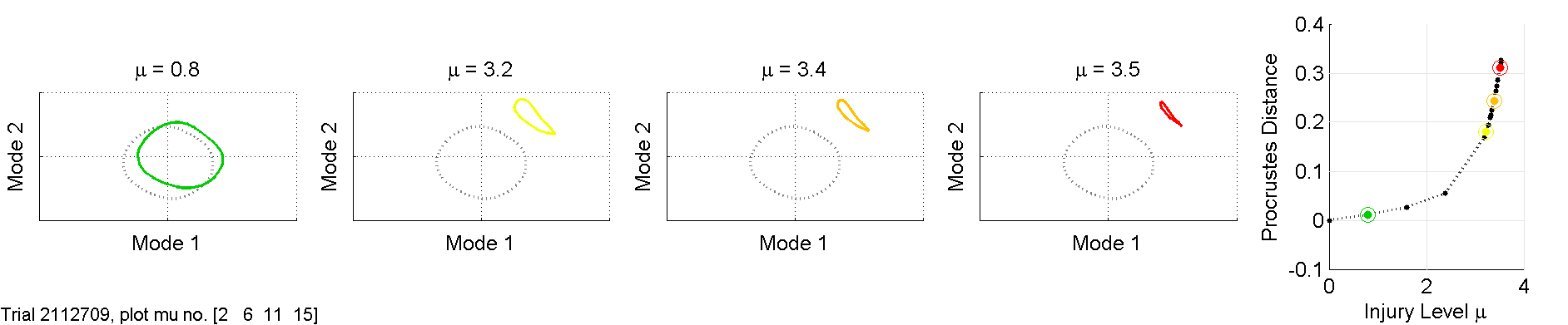

Supplement: S1 Figures — Figures similar to the rows of Fig 4, for all 1,447 trials conducted. (ZIP) [file pcbi.1005261.s002.zip › 2112709.png]
